# Supplementary material for: ClC-7 links PIKfyve inhibition to Rab-dependent LRRK2 activity at lysosomes
Source: J Cell Biol. 2026 Oct 20;225(11):e202511137. doi: 10.1083/jcb.202511137 (PMC13682606; doi:10.1083/jcb.202511137)
Supplement: Supplementary file 1 [file jcb_202511137_review_history.pdf]

JCB

# LRRK2 integrates Rab and GABARAP interactions to respond to distinct lysosomal stresses

Devin Clegg, Amanda Bentley-DeSousa, Agnes Roczniak-Ferguson, and Shawn Ferguson

*Corresponding Author(s): Shawn Ferguson, Yale University*



## Review Timeline:

| | |
|---|---|
| Submission Date: | 2025-11-19 |
| Editorial Decision: | 2025-12-18 |
| Revision Received: | 2026-07-20 |
| Editorial Decision: | 2026-08-12 |
| Revision Received: | 2026-08-27 |

*Monitoring Editor: Mark von Zastrow*

*Scientific Editor: Andrea Marat*

## Transaction Report:

(Note: With the exception of the correction of typographical or spelling errors that could be a source of ambiguity, letters and reports are not edited. The original formatting of letters and referee reports may not be reflected in this compilation.)

**DOI: https://doi.org/10.1083/jcb.202511137**

December 18, 2025

Re: JCB manuscript #202511137

Shawn Ferguson
Yale University

Dear Dr. Ferguson,

Thank you for submitting your manuscript entitled "LRRK2 integrates Rab and GABARAP interactions to sense and respond to distinct lysosomal stresses" as a JCB Report. It has now been reviewed by three external experts. All found your work interesting, and each was intrigued by your proposal that there exists a discrete PYKfyve / ClC7 pathway. However, all of the reviewers raised significant concerns and, accordingly, we are unable to accept your manuscript in its present form. However, we believe there may be a revision path if you can 1) address key critiques raised to solidify your new claims and 2) better contextualize your findings relative to previous work in the field.

If you wish to submit a revision, we'd ask you to focus on the following:

For reviewer 1, we ask you to pay particular attention to estimating the proportion of LRRK2 recovered in the lysosome fraction +/- apilomod and the question of whether the apilomod effect is specific to LRRK2-phosphorylated Rabs. Please also estimate the proportion of cellular Rabs recovered in the lysosome fraction, and discuss this in context with prior work suggesting that lysosomes are generally low in Rabs. Determining kinetics of the proposed sequential steps that you propose, as this reviewer suggests, would significantly add to the impact. However, if that isn't feasible, it would be sufficient to soften your interpretation of sequential steps to a proposal or "one possible model".

For reviewer 2, we ask you to focus on addressing the question of pRab recovery in your lysosome fraction +/- apilomod and provide additional support for your conclusion that LRRK2 is enriched there + apilomod. Imaging LRRK2 as suggested would be great but, if that is not feasible, please address whether residual non-lysosome elements may account for the increase + apilomod, as the reviewer suggests. This is a critical concern considering that the overall amount of cellular LRRK2 associated with lysosomes is probably low. One might assess this by doping the lysosome fraction with input to achieve a comparable degree of apparent contamination, but of course there are other options. Please also provide additional data or analysis to define the effect of apilomod on lysosome size, and clarify what you mean by lysosome size increase being "sufficient" to trigger the discrete pathway when compared to other manipulations that also increase lysosome size. This is important for contextualizing your results relative to what has been observed previously for manipulations known to drive CASM/GABARAP.

For reviewer 3, we ask that you to focus on determining if the apilomod-induced increase of Rabs on lysosomes requires ClC7, and whether the sucrose-induced Rab accumulation on lysosomes is dependent on ClC7 vs GABARAP. Please address or comment on the noted discrepancy of your results using apilomod with previous work using vacuolin-1. This reviewer, similar to reviewer 2, found your claim that lysosome size increase is "sufficient" to trigger the ClC7 - LRRK2 pathway unconvincing without substantial additional analysis. Please consider these critiques and modify your statements as appropriate.

All of the reviewers commented on some confusion in the text between what is new and what is confirmatory / previously shown. We therefore ask you to further clarify and highlight the key new claims that you are making, and contextualize this with previous knowledge more explicitly.

Thank you again for submitting your interesting work to JCB. While we regret being unable to accept your manuscript at present, we hope this summary is useful in planning your next steps.

While you are revising your manuscript, please also attend to the following editorial points to help expedite the publication of your manuscript. Please direct any editorial questions to the journal office.

GENERAL GUIDELINES:
Text limits: Character count for a Report is < 20,000, not including spaces. Count includes title page, abstract, introduction, the joint Results & Discussion, and acknowledgments. Count does not include materials and methods, figure legends, references, tables, or supplemental legends.

Figures: Reports may have up to 5 main text figures. To avoid delays in production, figures must be prepared according to the policies outlined in our Instructions to Authors, under Data Presentation, https://jcb.rupress.org/site/misc/ifora.xhtml. All figures in accepted manuscripts will be screened prior to publication.

***IMPORTANT: It is JCB policy that if requested, original data images must be made available. Failure to provide original images upon request will result in unavoidable delays in publication. Please ensure that you have access to all original

microscopy and blot data images before submitting your revision.***

Supplemental information: There are strict limits on the allowable amount of supplemental data. Reports may have up to 3 supplemental figures. Up to 10 supplemental videos or flash animations are allowed. A summary of all supplemental material should appear at the end of the Materials and methods section.

Please note that JCB now requires authors to submit Source Data used to generate figures containing gels and Western blots with all revised manuscripts. This Source Data consists of fully uncropped and unprocessed images for each gel/blot displayed in the main and supplemental figures. For assays performed using capillary electrophoresis and/or immunoassay-based detection, authors should instead provide the electropherogram graph(s) for each experiment, plotting fluorescence/chemiluminescence intensity vs. molecular weight/size. Please be sure to provide one Source Data file for each figure gels, blots, and/or capillary electrophoresis assays along with your revised manuscript files. File names for Source Data figures should be alphanumeric without any spaces or special characters (i.e., SourceDataF#, where F# refers to the associated main figure number or SourceDataFS# for those associated with Supplementary figures). For traditional gels and blots, the lanes of the gels/blots should be labeled as they are in the associated figure, the place where cropping was applied should be marked (with a box), and molecular weight/size standards should be labeled wherever possible. For capillary electrophoresis assays, each trace in the graph should be color-coded and labeled to indicate which protein, gene, or sample is being measured (please try to avoid red/green combinations to accommodate our color-blind readers).

Source Data files will be made available to reviewers during evaluation of revised manuscripts and, if your paper is eventually published in JCB, the files will be directly linked to specific figures in the published article.

Source Data Figures should be provided as individual PDF files (one file per figure). Authors should endeavor to retain a minimum resolution of 300 dpi or pixels per inch. Please review our instructions for export from Photoshop, Illustrator, and PowerPoint here: https://rupress.org/jcb/pages/submission-guidelines#revised

The typical timeframe for revisions is three to four months. If you anticipate any difficulties in meeting this aforementioned revision time limit, please contact us and we can work with you to find an appropriate time frame for resubmission. Please note that papers are generally considered through only one revision cycle, so any revised manuscript will likely be either accepted or rejected.

When submitting the revision, please include a cover letter addressing the reviewers' comments point by point. Please also highlight all changes in the text of the manuscript.

We hope that the comments below will prove constructive as your work progresses. We would be happy to discuss them further once you've had a chance to consider the points raised in this letter.

Thank you for this interesting contribution to Journal of Cell Biology. You can contact us at the journal office with any questions at cellbio@rockefeller.edu.

Sincerely,

Mark von Zastrow
Monitoring Editor

Andrea L. Marat
Deputy Editor

Journal of Cell Biology

--------------------------------------------------------------------------------

Reviewer #1 (Comments to the Authors (Required)):

Activating mutations in LRRK2 kinase are linked to Parkinson's, so there is great interest in understanding how LRRK2 is activated and regulated. In previous work, these authors showed that in macrophages, LIR domains on LRRK2 mediated its recruitment to LLOME-damaged lysosomes via binding to lipidated GABARAP generated by the CASM pathway. Here they explore a different mode of LRRK2 activation that is seen upon inhibition of PIKfyve kinase that generates PI3,5P2 on late endosomes and lysosomes. This latter process involves swelling of lysosomes, and the authors could recapitulate the phenotype using sucrose. They show that swelling leads to the recruitment of Rabs to lysosome surfaces where they recruit and appear to activate LRRK2, independent of CASM. The paper is carried out to a high standard, thus the story deserves to be presented in JCB, after consideration of the following points.

1. Page 2, line 2. "LRRK2 localizes to lysosomal membranes". Actually, only a small fraction of LRRK2 is on lysosomal

membranes in RAW macrophages, even under stress (~10%?). Please change the text in line 2 to something like, "LRRK2 is recruited to stressed or damaged lysosomes." Also, please provide a value for how much of the total pool is recruited to lysosomes under conditions of apilimod and/or sucrose. This is important and can be added with description of Fig. 1H-I.
2. Rabs are not usually on lysosomes (except Rab32 for example which is on lysosome-related organelles; macrophages may have more than other cell types). For at least some of the Rabs in Fig. 4F, what fraction of the total are on isolated lysosomes in these cells? Why/how are these Rabs re-localized to lysosomes upon swelling? Please discuss in text. Is it because they are all phosphorylated and thus trapped on lysosomes because GDI cannot bind? Please include a non-LRRK2 substrate (non-lysosomal) Rab to see if this is a general phenomenon or due to LRRK2 modification and entrapment. (See point 4)
3. Page 5. The pRab8 antibody is not specific for pRab8 so please clarify this in the text on first mention.
4. Fig. S3B. The authors would like to conclude that Rabs are recruited before LRRK2. This conclusion should likely be bolstered. Apilimod and MLi2 seem to be added together. Are the Rabs recruited to lysosomes in apilimod-treated LRRK2 KO cells or with overnight MLi-2? Also note that RAW cells have other Rab12 kinases that are insensitive to MLi-2. I don't know about Rab8, but it might be worth blotting for pRab12. Yet why is there very little recruitment of Rab10? I don't know how fast before you see the apilimod phenotype, but please soften the statement that Rabs are onto lysosomes first without determining the kinetics of Rab and LRRK2 recruitment. The actual kinetics are important here and would be a nice addition.
5. Page 8. "LRRK2 is a coincidence detector (sensing damage)" Not really if Rabs are recruited first-LRRK2 is just binding its partners. Accumulation might be seen because pRabs cannot be retrieved by GDI. Also, LRRK2 recruitment is somewhat later than ESCRT recruitment which is a real damage detector, at least after LLOME treatment. Because the authors have seen a new phenomenon of apparent Rab recruitment to swollen lysosomes, they should document the specificity of the Rabs involved (including non-LRRK2 substrate Rabs) and propose and discuss a pathway to explain this unusual behavior. Is there general membrane fusion? Other organelle markers are not seen, or? Is it faster than that seen in LLOME? Kinetics would add a lot.
6. (Minor) The figures use many different formats (bold, non bold, lines of different weight) and could use some graphic improvement. Otherwise, well done overall.

Reviewer #2 (Comments to the Authors (Required)):

Clegg and colleagues explore how osmotic stress of the endolysosomal system regulates LRRK2 kinase activity. They show that conditions that induce lysosomal enlargement, such as PIKfyve inhibition or sucrose treatment, lead to increased LRRK2-dependent phosphorylation of its Rab substrates and do so in a manner independent of the previously described pathway dependent on GABARAP. The authors propose that LRRK2 acts as a "coincidence detector" of lysosomal stress to integrate multiple different types of lysosomal dysfunction to activate LRRK2.

This work adds to a growing body of work suggesting that various manipulations that cause lysosomal dysfunction can drive LRRK2 hyperactivation and provide some new mechanistic understanding by implicating CLC-7 as a key mediator. While the study aims to better understand the mechanisms by which different facets of lysosomal dysfunction can ultimately lead to LRRK2 activation, the degree of novelty and the robustness of the evidence separating lysosomal swelling from lysosomal damage remain unclear.

Additional work is needed to further strengthen and clarify the authors' claims to warrant publication in JCB.

Main points of the manuscript and associated concerns:
1)PIKFYVE inhibition triggers the recruitment of LRRK2 to the lysosome and leads to increased LRRK2 activity:

a)The authors assess LRRK2 levels in lysosomes isolated using the SPION-based method following apilimod treatment and observe an increase in LRRK2 levels in their SPION-isolated fractions. However, their western blot analysis also shows increased contamination with markers of the Golgi and ER following apilimod treatment, complicating the interpretation that the increased levels of LRRK2 detected are truly lysosomal. Orthogonal assessments of LRRK2 localization to lysosomes (for example, through imaging-based approaches) are needed to add confidence that apilimod treatment increases the localization of LRRK2 to lysosomes.

b)The authors suggest that apilimod treatment leads to increased LRRK2 localization to lysosomes and show increased Rab10 phosphorylation. The interpretation is that the increase in Rab10 phosphorylation is occurring on lysosomes (in the abstract the authors state that "osmotic swelling leads to the accumulation of multiple Rabs on lysosomes"); however, no data is provided to establish that Rab levels or Rab phosphorylation are increased at the lysosome. Additional studies are needed (either using SPION-isolated lysosomes or imaging-based approaches) to demonstrate that apilimod leads to an increase in Rab10 phosphorylation on lysosomes.

c)The authors show that apilimod treatment leads to enlarged lysosomes as well as increased pRab10 and LRRK2 recruitment to purified lysosomes (Fig. 1). These observations are consistent with prior reports demonstrating that apilimod treatment can promote LRRK2 activation (PMID: 38299383), and that perturbations that cause endosomal lysosomal enlargement (such as VPS34 inhibition) can also trigger LRRK2 activation (PMID: 37141099). The manuscript should more explicitly contextualize these findings within the existing literature and clearly articulate the novel insights from the present study (e.g., the requirement

for ClC-7 and independence on GABARAP).

2)ClC-7 is required for LRRK2 activation downstream of PIKfyve inhibition:

a)Although the authors claim that CLC-7 KO cells have reduced lysosomal swelling, the representative images still show what looks like many enlarged lysosomes in the CLC-7 KO cells following apilimod treatment. Quantification of lysosome size is needed to add confidence that CLC-7 deletion is able to rescue the apilimod-dependent enlarged lysosome phenotype in this cell model.

b)The authors include nice data showing that the CLC-7 KO cells have reduced Rab10 phosphorylation following apilimod treatment, supporting their claims that apilimod-mediated LRRK2 activation requires CLC-7. However, a non-significant trend of increased pRab10 persists (as seen in their western blot images), suggesting that other mechanisms may contribute to this. The authors should add additional evidence that CLC-7 has been deleted in these cells (via western blot analysis) and soften their language on this being an absolute requirement.

c)The authors often normalize to one condition for their quantification (e.g. Apilimod treated cells in Fig. 2F), which is helpful for understanding the magnitude of effect, but this is done so in a way that means one condition in every experiment lacks error bars. The data should reanalyze their data in a way that maintains the variance within each condition, including the condition used for normalization across experiments. This should be done for Fig. 1C, E, G, I; Fig. 2D, F, and H; Fig. 3C, E, and H, Fig. 4C, E, and H, and Fig. 5B, D, F, and H.

3)Osmotic swelling is sufficient to activate LRRK2:

a)The authors try and establish that lysosomal swelling, and not other aspects of lysosomal dysfunction, are driving LRRK2 activation following apilimod treatment. They suggest that BafA1 and phloretin treatment abolish the enlarged endolysosomal phenotype caused by apilimod. However, low-resolution brightfield images are included in Figure 3A to establish this point. To enable comparison across studies, confocal images with LAMP staining (as done in Figures 1 and 2) and accompanying quantification of lysosome size are needed to confirm that BafA1 and phloretin reduce endolysosomal size. Similarly, confocal imaging and quantification of lysosome size should also be done for sucrose treatment analysis currently shown in Fig. 3F.

b)While sucrose treatment clearly leads to increased Rab10 phosphorylation, it is unclear whether it does so solely through its effects on endolysosomal size. High dose sucrose treatment can also induce lysosomal damage. To strengthen the authors' claims that this effect is due to endolysosomal swelling, assessment of lysosomal damage (e.g. imaging of galectin-3 levels on lysosomes following sucrose treatment) would be helpful to add confidence that this effect on Rab phosphorylation is likely due to changes in endolysosomal size rather than membrane integrity issues. In general, additional data is needed under the conditions used to add confidence that the effects observed are due to changes in lysosome swelling and not increased LMP.

4)Physiological relevance and scientific advance:
a)The authors propose an elegant mechanism by which LRRK2 can be activated in response to several different types of endolysosomal dysfunction. However, the physiological relevance of PIKFYVE inhibition and osmotic swelling with respect to PD remains unclear. Additional text should be added to the discussion that explores potential reasons why LRRK2 activation might be helpful in sensing and responding to endolysosomal swelling. While the potential benefits for repairing or removing damaged lysosomes is clear, it is not as clear why LRRK2 might be activated in response to endolysosomal swelling and what the downstream consequences of LRRK2 activation on addressing/correcting endolysosomal enlargement might be.
b)The novelty of the work should be further articulated in the discussion, as Rab-dependent recruitment of LRRK2 to dysfunctional lysosomes has already been established under many conditions (already well-cited in the text) and apilimod has been shown previously to lead to LRRK2 activation.

Reviewer #3 (Comments to the Authors (Required)):

Summary:

In the manuscript "LRRK2 integrates Rab and GABARAP interactions to sense and respond to distinct lysosomal stresses", Clegg et al., find a role for the Cl-/H+ exchanger, ClC7, in LRRK2 activation upon pharmacological inhibition of PIKfyve activity. With this finding, they distinguish ClC7-mediated LRRK2 activation from LRRK2 activity mediated by CASM-related GABARAP lipidation induced by various stimuli. While the role of ClC7 and the distinct requirements of ClC7 vs GABARAP in LRRK2 activation with different stimuli is novel and interesting, these data are far too preliminary and require

further interrogation into the mechanisms of how inhibiting PIKfyve activity and ClC7 induce LRRK2 activation. The remaining half of the manuscript that focuses on Rab-dependent activation of LRRK2 confirms prior literature (Dhekne et al., 2023, Vides et al., 2022, Zhu et al., 2023, Wang et al, 2023), but does not add to our understanding of the mechanism of Rab-mediated LRRK2 activation, putting the novelty of this data into question. Lastly, the claims of LRRK2 activation downstream of osmotic "swelling" and the distinction of deacidification in terms of CASM-related GABARAP lipidation are confusing, inconsistent with the literature, and need major revision.

Major Comments:

1. It's not clear how LRRK2 integrates multiple upstream cues through "parallel interactions" with Rab GTPases and GABARAP - GABARAP is required for LRRK2 activation with certain stimuli per the authors' prior publication (Bentley-DeSousa et al., 2025), but is GABARAP required for Rab recruitment? Are Rabs required for GABARAP lipidation? Experimentally answering these questions is necessary to determine whether their interactions with LRRK2 are "parallel", otherwise claims need to be revised.

2. The data regarding Rab recruitment is intriguing - more analysis of what is required for Rab recruitment, with support of imaging data, would strengthen our understanding of this upstream step of LRRK2 activation - Is ClC7 required for Rab recruitment? Does phloretin and BafA1 block Rab recruitment?

3. Eguchi et al., 2018 found that LRRK2 is not recruited to lysosomes upon Vacuolin-1 treatment, can the authors address this discrepancy with imaging of LRRK2 recruitment with Apilimod treatment?

4. It is not clear how "lysosomal swelling is sufficient to activate LRRK2" based on the data presented. Lysosomal swelling/enlargement is regulated by solute and water carriers (PMID: 36219209), as well as lipid transfer proteins (Yang et al, 2025), and various other mechanisms. Any of these molecular events could be required for LRRK2 activation independently or in correlation with swelling. It would be technically difficult to dissect these events and their contributions to LRRK2 activation in a complex cellular system and would require a simplified in vitro system to rigorously test sufficiency. The authors should revise the claims regarding swelling to acknowledge these limitations.

The authors appear to come to this conclusion as they show sucrose-mediated lysosomal swelling and LRRK2 activation does not require ClC7 (Fig. 3F-H), however an alternative explanation could be that the mechanism of swelling and/or LRRK2 activation with sucrose is just not regulated by ClC7. The authors show that ClC7 is not required for LRRK2 activation by other stimuli that induce lysosomal swelling (Nigericin PMID: 40372734 and LLOMe), which raises the question of whether CASM-mediated GABARAP is induced by sucrose stress and required for LRRK2 activation, rather than ClC7? Additionally, lysosome enlargement induced by Apilimod is reduced in the ClC7KO (Fig. 2B), but it does not appear to be completely blocked in the representative image, which would be consistent with data in Leray et al., 2022. Quantification of lysosome size would help determine whether and to what extent swelling occurs in the ClC7KO. If there still is some lysosomal swelling in the ClC7KO, in which LRRK2 activation does not occur, then lysosomal swelling would not be sufficient.

5. Similarly, the distinction that is made between manipulations that induce lysosome enlargement/swelling and deacidification, as if CASM-mediated GABARAP lipidation is only related to deacidification, is confusing and inconsistent with the literature. Stimuli that induce CASM-mediated ATG8 lipidation, such as LLOMe, Nigericin, and Monensin, also induce enlargement/swelling of lysosomes, and TRPML1 agonists that do not deacidify lysosomes, but rather hyperacidify (PMID: 39433126, PMID: 32858116), like PIKfyve inhibition (Leray et al., 2022) induce CASM and related LRRK2 activation, as the authors have previously shown (Bentley-DeSousa et al., 2025). Rather, it appears from the data that stimuli causing ionic/osmotic imbalances and lysosomal swelling also induce LRRK2 activation through different mechanisms, requiring either ClC7 or GABARAP lipidation. Please revise and clarify the claims around this distinction.

With these considerations, I am wondering whether PIKfyve inhibition induces CASM-mediated GABARAP lipidation? And, whether ClC7 regulates swelling with Nigericin and LLOMe? Answering these questions would help further dissect the differences of these 2 pathways mediating LRRK2 activation.

6. The V-ATPase is also reported to be regulated by PIKfyve/Fab1 (PMID: 27335171, PMID: 30730983, PMID: 29791245, PMID: 32656214). The authors demonstrate that swelling and LRRK2 activation induced by Apilimod are blocked by BafA1 (Fig. 3A-C) - is the V-ATPase contributing to swelling and LRRK2 activation by PIKfyve inhibition or does BafA1 just deplete the available protons for ClC7 activity?

7. Clcn7 KO validation (Fig. S2A) is insufficient - it is not clear how the PCR analysis used can distinguish between a frame shifting or in-frame deletion. The authors need to show protein level KO of ClC7, or at least genetic and transcript level analysis to validate the KO if there is not a good commercially available antibody. Please also include the guide RNA sequence, exon target information, and primer sequences for PCR validation used for KO cell lines in the methods for proper reporting.

8. The discussion reads more like a general review of the literature and requires more discussion of the conclusions that can be

drawn from the results of the manuscript within that context.

Minor Comments:

1. Authors claim to test "biophysical" approaches, however it is unclear what biophysical approaches were used. If the authors mean to suggest the sucrose-mediated experiments are biophysical, sucrose-mediated swelling is not purely biophysical, and is also driven by osmotic regulation by aquaporins (Yang et al., 2025), and likely other solute carriers. The authors should revise this language to not confuse the reader.

2. Figure legends for Fig S4 do not match the panel labels in the figure. Please revise and ensure consistency in other figure legends.

3. The plotted data in figures appears to be normalized, but normalization methods are not reported in the methods or figure legends. Please add whether and how normalization methods were used, and whether the statistical analyses were done on normalized or raw values.

4. Please use standard deviation or confidence intervals for error bars, rather than SEM, to show variability in the data set transparently (PMID: 31145670).

5. What is Pan-pRab on the figure 5H Y axis? Does not match figure legend.

6. It is stated in the significance statement that "Osmotic swelling drives strong Rab-dependent activation, whereas deacidification requires CASM-mediated GABARAP lipidation as a scaffold for LRRK2 activation at lysosomes" makes it seem like Rabs are not required in conditions of deacidification, however the authors show that LRRK2-Rab interactions are required with Nigericin and LLOMe in Fig 5G-H. Later it is correctly stated in the introduction that "LRRK2-Rab interactions are essential for activation by both swelling and CASM-dependent stimuli, identifying Rabs as a unifying hub through which diverse lysosomal stress pathways converge on LRRK2". Please revise for consistency.

7. Rab accumulation does not appear to be tested in the section titled "Lysosomal swelling drives Rab accumulation and LRRK2 activation". Please revise.

**Response to Reviewers**

*Thank you for submitting your manuscript entitled "LRRK2 integrates Rab and GABARAP interactions to sense and respond to distinct lysosomal stresses" as a JCB Report. It has now been reviewed by three external experts. All found your work interesting, and each was intrigued by your proposal that there exists a discrete PYKfyve / ClC7 pathway. However, all of the reviewers raised significant concerns and, accordingly, we are unable to accept your manuscript in its present form. However, we believe there may be a revision path if you can 1) address key critiques raised to solidify your new claims and 2) better contextualize your findings relative to previous work in the field.*

Thank-you for the constructive feedback. In our revised manuscript have performed new experiments and have updated the text to address each of the concerns that were raised.

*If you wish to submit a revision, we'd ask you to focus on the following:*

*For reviewer 1, we ask you to pay particular attention to estimating the proportion of LRRK2 recovered in the lysosome fraction +/- apilomod and the question of whether the apilomod effect is specific to LRRK2-phosphorylated Rabs. Please also estimate the proportion of cellular Rabs recovered in the lysosome fraction, and discuss this in context with prior work suggesting that lysosomes are generally low in Rabs. Determining kinetics of the proposed sequential steps that you propose, as this reviewer suggests, would significantly add to the impact. However, if that isn't feasible, it would be sufficient to soften your interpretation of sequential steps to a proposal or "one possible model".*

As outlined in more detail below, we have both generated new data and have modified our claims to address these concerns.

*For reviewer 2, we ask you to focus on addressing the question of pRab recovery in your lysosome fraction +/- apilomod and provide additional support for your conclusion that LRRK2 is enriched there + apilomod. Imaging LRRK2 as suggested would be great but, if that is not feasible, please address whether residual non-lysosome elements may account for the increase + apilomod, as the reviewer suggests. This is a critical concern considering that the overall amount of cellular LRRK2 associated with lysosomes is probably low. One might assess this by doping the lysosome fraction with input to achieve a comparable degree of apparent contamination, but of course there are other options. Please also provide additional data or analysis to define the effect of apilomod on lysosome size, and clarify what you mean by lysosome size increase being "sufficient" to trigger the discrete pathway when compared to other manipulations that also increase lysosome size. This is important for contextualizing your results relative to what has been observed previously for manipulations known to drive CASM/GABARAP.*

In our responses to reviewer 2, we help to address the question of LRRK2 site-of-action with immunofluorescence experiments in both Raw264.7 cells and human-iPSC-derived microglia showing the lysosomal localization of phospho-Rab10 following PIKfyve inhibition (new Figure 1 L and M). Additionally, these experiments did not reveal major phospho-Rab10 localization outside of lysosomes. We have also moderated our interpretations of the impacts of lysosome size.

*For reviewer 3, we ask that you to focus on determining if the apilomod-induced increase of Rabs on lysosomes requires ClC7, and whether the sucrose-induced Rab accumulation on lysosomes is dependent on ClC7 vs GABARAP. Please address or comment on the noted discrepancy of your results using apilomod with previous work using vacuolin-1. This reviewer, similar to reviewer 2, found your claim that lysosome size increase is "sufficient" to trigger the ClC7 - LRRK2 pathway unconvincing without substantial additional analysis. Please consider these critiques and modify your statements as appropriate.*

We have now addressed both the relationship between ClC-7 and apilimod-induced Rab accumulations at lysosomes as well as between sucrose and GABARAP. Given that the interpretation of the sucrosome experiments turned out to be more complex than initially anticipated and not central to the main message, we have removed these experiments and have moderated our claims accordingly. Likewise, we acknowledge the challenges in drawing direct conclusions about the relationship between lysosome size and LRRK2 activity and have been more careful to not over-reach in our claims.

*All of the reviewers commented on some confusion in the text between what is new and what is confirmatory / previously shown. We therefore ask you to further clarify and highlight the key new claims that you are making, and contextualize this with previous knowledge more explicitly.*

In the process of updating the manuscript with new data and its interpretation, we have also focusing on making our new findings more evident. Briefly, previous work independently showed that PIKfyve inhibition can increase LRRK2 activity (only minimally shown and without mechanistic insights), that CASM-lipidated GABARAP recruits LRRK2 following lysosome damage or deacidification, and that several Rabs bind and regulate LRRK2. The overall relationship between regulation of LRRK2 by GABRAP and Rabs was not well defined. **New findings:** We identified ClC-7 as a previously unrecognized intermediary between PIKfyve inhibition and LRRK2 signaling from lysosomes. We further show that PIKfyve inhibition broadly increases lysosomal abundance of multiple Rabs independently of LRRK2 kinase activity, while ClC-7 is required for the accumulation of a subset of these Rabs, including Rab12 and Rab32. We found that this ClC-7- and Rab-dependent pathway for LRRK2 regualtion does not require CASM or GABARAP, whereas CASM-inducing stresses require Rab–LRRK2 interactions in addition to GABARAP. Together, these new findings connect lysosomal ion homeostasis and Rab organization to LRRK2 regulation, advancing lysosome cell biology while revealing mechanisms through which lysosomal dysfunction may contribute to aberrant LRRK2 signaling in Parkinson’s disease.

-----------------------------------------------------------------------------

*Reviewer #1 (Comments to the Authors (Required)):*

*Activating mutations in LRRK2 kinase are linked to Parkinson's, so there is great interest in understanding how LRRK2 is activated and regulated. In previous work, these authors showed that in macrophages, LIR domains on LRRK2 mediated its recruitment to LLOME-damaged lysosomes via binding to lipidated GABARAP generated by the CASM pathway. Here they explore a different mode of LRRK2 activation that is seen upon inhibition of PIKfyve kinase that generates PI3,5P2 on late endosomes and lysosomes. This latter process involves swelling of lysosomes, and the authors could recapitulate the phenotype using sucrose. They show that swelling leads to the recruitment of Rabs to lysosome surfaces where they recruit and appear to activate LRRK2, independent of CASM. The paper is carried out to a high standard, thus the story deserves to be presented in JCB, after consideration of the following points.*

We appreciate this positive evaluation.

*1. Page 2, line 2. "LRRK2 localizes to lysosomal membranes". Actually, only a small fraction of LRRK2 is on lysosomal membranes in RAW macrophages, even under stress (~10%?). Please change the text in line 2 to something like, "LRRK2 is recruited to stressed or damaged lysosomes." Also, please provide a value for how much of the total pool is recruited to lysosomes under conditions of apilimod and/or sucrose. This is important and can be added with description of Fig. 1H-I.*

We have revised the Abstract as suggested to state that LRRK2 is recruited to stressed or damaged lysosomes.

We agree that only a small fraction of total cellular LRRK2 is expected to associate with lysosomes, even under stress conditions. However, we do not think that the lysosome purification data provides a reliable basis for calculating an absolute percentage of total LRRK2 on lysosomes. Such a calculation would require quantitative recovery of LRRK2 across all purification fractions, an accurate measure of lysosome capture efficiency, and assumptions about how efficiently homogenization disrupts cells while preserving intact lysosomes.

To address the reviewer's underlying concern about the localization and relevance of the lysosome-associated LRRK2 pool, we added new immunofluorescence experiments using phospho-Rab10 as a readout of sites of LRRK2 activity, as previously established by Dhekne et al (eLife, 2022). In these experiments, we found that phospho-Rab10 was strongly enriched on a subset of enlarged LAMP1-positive lysosomes following apilimod treatment in both RAW264.7 cells and human iPSC-derived microglia. These data indicate that although the lysosome-associated pool of LRRK2 is likely to represent only a minor fraction of total cellular LRRK2, it corresponds to a major site of stimulus-induced LRRK2 signaling under these conditions.

We have updated the Results section describing Fig. 1 to include these new data and to clarify that our lysosome purification experiments support increased lysosomal recruitment of LRRK2 after apilimod treatment, whereas the phospho-Rab10 immunofluorescence identifies swollen lysosomes as sites of LRRK2-dependent kinase activity.

*2. Rabs are not usually on lysosomes (except Rab32 for example which is on lysosome-related organelles; macrophages may have more than other cell types). For at least some of the Rabs in Fig. 4F, what fraction of the total are on isolated lysosomes in these cells? Why/how are these Rabs re-localized to lysosomes upon swelling? Please discuss in text. Is it because they are all phosphorylated and thus trapped on lysosomes because GDI cannot bind? Please include a non-LRRK2 substrate (non-lysosomal) Rab to see if this is a general phenomenon or due to LRRK2 modification and entrapment. (See point 4)*

We were surprised to see that the abundance of so many different Rabs increased on lysosomes following apilimod treatment of cells (and also in response to LLOME and nigericin). However, in contrast to LAMP1, the enrichment of Rabs on purified lysosomes was much more modest. This relocalization of Rabs in response to these perturbations represents an unexpected response to these lysosomal stresses. Analysis of more Rabs, including non-LRRK2 substrates such as Rab5, 7, 9 and 32 shows that even they respond to these stimuli. The underlying mechanism is not known but given that they do not all share common GAPs or GEFs, it seems like a loss of extraction by GDI could be involved. In addition to the new data, we have modified the discussion to better explain these ideas.

*3. Page 5. The pRab8 antibody is not specific for pRab8 so please clarify this in the text on first mention.*
Good point. We have clarified this now.

*4. Fig. S3B. The authors would like to conclude that Rabs are recruited before LRRK2. This conclusion should likely be bolstered. Apilimod and MLi2 seem to be added together. Are the Rabs recruited to lysosomes in apilimod-treated LRRK2 KO cells or with overnight MLi-2? Also note that RAW cells have other Rab12 kinases that are insensitive to MLi-2. I don't know about Rab8, but it might be worth blotting for pRab12. Yet why is there very little recruitment of Rab10? I don't know how fast before you see the apilimod phenotype, but please soften the statement that Rabs are onto lysosomes first without determining the kinetics of Rab and LRRK2 recruitment. The actual kinetics are important here and would be a nice addition.*

In Figure S4C, we now show that while MLI2 is very effective at inhibiting LRRK2, as judged by loss of the phospho-Rab10 signal, the lysosomal accumulation of total amounts of Rabs 8, 10 and 12 were not affected. However, given that this is not absolutely central to our main conclusions, we have softened statements about the order of recruitment.

*5. Page 8. "LRRK2 is a coincidence detector (sensing damage)" Not really if Rabs are recruited first-LRRK2 is just binding its partners. Accumulation might be seen because pRabs cannot be retrieved by GDI. Also, LRRK2 recruitment is somewhat later than ESCRT recruitment which is a real damage detector, at least after LLOME treatment. Because the authors have seen a new phenomenon of apparent Rab recruitment to swollen lysosomes, they should document the specificity of the Rabs involved (including non-LRRK2 substrate Rabs) and propose and discuss a pathway to explain this unusual behavior. Is there general membrane fusion? Other organelle markers are not seen, or? Is it faster than that seen in LLOME? Kinetics would add a lot.*

In editing the manuscript, we have now tried to emphasize that LRRK2 is integrating a response to lysosome stresses through its Rab and GABARAP interactions rather than acting directly as a sensor. We removed "sensing" from the title to reflect this. Also, as outlined above, we now show that this regulation of Rab abundance at lysosomes extends beyond the Rabs that are directly implicated in the LRRK2 pathway (either as recruiters or as substrates). The underlying reasons for this Rab regulation remain unknown. We acknowledge this in the discussion. Although such mechanisms of Rab regulation are of potential interest, we have focused this manuscript on the regulation of LRRK2.

*6. (Minor) The figures use many different formats (bold, non bold, lines of different weight) and could use some graphic improvement. Otherwise, well done overall.*

Thanks for catching this. We have addressed it in the revised manuscript.

*Reviewer #2 (Comments to the Authors (Required)):*

*Clegg and colleagues explore how osmotic stress of the endolysosomal system regulates LRRK2 kinase activity. They show that conditions that induce lysosomal enlargement, such as PIKfyve inhibition or sucrose treatment, lead to increased LRRK2-dependent phosphorylation of its Rab substrates and do so in a manner independent of the previously described pathway dependent on GABARAP. The authors propose that LRRK2 acts as a "coincidence detector" of lysosomal stress to integrate multiple different types of lysosomal dysfunction to activate LRRK2.*

*This work adds to a growing body of work suggesting that various manipulations that cause lysosomal dysfunction can drive LRRK2 hyperactivation and provide some new mechanistic understanding by implicating CLC-7 as a key mediator. While the study aims to better understand the mechanisms by which different facets of lysosomal dysfunction can ultimately lead to LRRK2 activation, the degree of novelty and the robustness of the evidence separating lysosomal swelling from lysosomal damage remain unclear.*

*Additional work is needed to further strengthen and clarify the authors' claims to warrant publication in JCB.*

Through our efforts to address the reviewer comments we have strengthened the support for our main message related to the way in which Rab and GABARAP abundance at lysosomes is integrated by LRRK2 to signal downstream of multiple stresses. The strength of our manuscript is this integration rather than any of the single observations.

*Main points of the manuscript and associated concerns:*
*1)PIKFYVE inhibition triggers the recruitment of LRRK2 to the lysosome and leads to increased LRRK2 activity:*

*a)The authors assess LRRK2 levels in lysosomes isolated using the SPION-based method following apilimod treatment and observe an increase in LRRK2 levels in their SPION-isolated fractions. However, their western blot analysis also shows increased contamination with markers of the Golgi and ER following apilimod treatment, complicating the interpretation that the increased levels of LRRK2 detected are truly lysosomal. Orthogonal assessments of LRRK2 localization to lysosomes (for example, through imaging-based approaches) are needed to add confidence that apilimod treatment increases the localization of LRRK2 to lysosomes.*
We now show via immunofluorescence that lysosomes are the major site of LRRK2-dependent Rab10 phosphorylation in response to PIKfyve inhibition with apilimod in both Raw264.7 cells and human iPSC-microglia (Fig. 1L and M). This complements the immunoblot analysis of LRRK2 abundance on purified lysosomes (Fig. 1G-H and elsewhere). With respect to Golgi contamination, we acknowledge that we sometimes see more GM130 in the lysosome fraction after apilimod treatment. However, there is a clear depletion compared to the starting material and the amounts are much more minimal compared to lysosome proteins or the Rabs that we are studying. To help the reader better able to judge this for themselves, we also now explain in our figure legends that "Images for the lysate and lysosome fractions come from the same exposure of the same blot that was loaded with 5μg lysate and 0.5 μg of lysosome protein.".

*b)The authors suggest that apilimod treatment leads to increased LRRK2 localization to lysosomes and show increased Rab10 phosphorylation. The interpretation is that the increase in Rab10 phosphorylation is occurring on lysosomes (in the abstract the authors state that "osmotic swelling leads to the accumulation of multiple Rabs on lysosomes"); however, no data is provided to establish that Rab levels or Rab phosphorylation are increased at the lysosome. Additional studies are needed (either using SPION-isolated lysosomes or imaging-based approaches) to demonstrate that apilimod leads to an increase in Rab10 phosphorylation on lysosomes.*
Evidence for Rab10 phosphorylation on lysosomes is now supported by both immunoblotting and immunofluorescence experiments (Figs. 1G-I, L-M).

*c)The authors show that apilimod treatment leads to enlarged lysosomes as well as increased pRab10 and LRRK2 recruitment to purified lysosomes (Fig. 1). These observations are consistent with prior reports demonstrating that apilimod treatment can*

*promote LRRK2 activation (PMID: 38299383), and that perturbations that cause endosomal lysosomal enlargement (such as VPS34 inhibition) can also trigger LRRK2 activation (PMID: 37141099). The manuscript should more explicitly contextualize these findings within the existing literature and clearly articulate the novel insights from the present study (e.g., the requirement for ClC-7 and independence on GABARAP).*

We have tried to better highlight the importance of the requirement for ClC-7. However, more importantly, through our comparison of the requirements for Rabs and GABARAP for LRRK2 activation by different lysosomes stresses, we have arrived at a model that helps to integrate and extend the findings of previous studies that have separately reported on roles for Rabs and GABARAP in LRRK2 regulation but did not provide insight into how they collectively contribute. With respect to VPS34 inhibition with SAR405, we did not observe a significant effect on activity of endogenously expressed LRRK2 in Raw264.7 cells (Bentley-DeSousa et al, JCB, 2025). This could reflect a difference between cell types and experimental models. Note that the previous report of VPS34-dependent (also using SAR405) regulation of LRRK2 came from analysis of over-expressed LRRK2 in HEK293 cells (Rinaldi et al, Cell Reports, 2023).

*2)ClC-7 is required for LRRK2 activation downstream of PIKfyve inhibition:*

*a)Although the authors claim that CLC-7 KO cells have reduced lysosomal swelling, the representative images still show what looks like many enlarged lysosomes in the CLC-7 KO cells following apilimod treatment. Quantification of lysosome size is needed to add confidence that CLC-7 deletion is able to rescue the apilimod-dependent enlarged lysosome phenotype in this cell model.*

Figure 2B of the revised manuscript now shows a quantification of the impacts of apilimod and ClC-7 KO on lysosome volume. While ClC-7 KO does not fully block the ability of apilimod to enlarge lysosomes, the effect is significant.

*b)The authors include nice data showing that the CLC-7 KO cells have reduced Rab10 phosphorylation following apilimod treatment, supporting their claims that apilimod-mediated LRRK2 activation requires CLC-7. However, a non-significant trend of increased pRab10 persists (as seen in their western blot images), suggesting that other mechanisms may contribute to this. The authors should add additional evidence that CLC-7 has been deleted in these cells (via western blot analysis) and soften their language on this being an absolute requirement.*

After reading the literature and consulting with a ClC-7 expert (Joe Mindell, NIH) we were unable to find a suitable ClC-7 antibody for immunoblotting in mouse cells. However, our PCR analysis of ClC-7 KO clones clearly shows a large deletion of the target locus (Fig. S2B). We have furthermore analyzed the sequence of these deletions and have confirmed that they result in deletions of exon 5 that are predicted to fully disrupt the coding sequence of ClC-7. We have updated our methods section to provide this key information regarding validation of the ClC-7 KO. In light of the fact that the

ClC-7 KO cells retain a small amount of Rab10 phosphorylation (Fig. 2C and D) we have also been more careful to moderate our conclusions accordingly.

*c)The authors often normalize to one condition for their quantification (e.g. Apilimod treated cells in Fig. 2F), which is helpful for understanding the magnitude of effect, but this is done so in a way that means one condition in every experiment lacks error bars. The data should reanalyze their data in a way that maintains the variance within each condition, including the condition used for normalization across experiments. This should be done for Fig. 1C, E, G, I; Fig. 2D, F, and H; Fig. 3C, E, and H, Fig. 4C, E, and H, and Fig. 5B, D, F, and H.*

We appreciate the reviewer's comment regarding normalization and the display of variance for reference conditions. We reanalyzed all our data using the raw (non-normalized) values and have updated figures with the outcomes of these new analyses. These changes help with the presentation of our results but did not change major conclusions.

*3)Osmotic swelling is sufficient to activate LRRK2:*

*a)The authors try and establish that lysosomal swelling, and not other aspects of lysosomal dysfunction, are driving LRRK2 activation following apilimod treatment. They suggest that BafA1 and phloretin treatment abolish the enlarged endolysosomal phenotype caused by apilimod. However, low-resolution brightfield images are included in Figure 3A to establish this point. To enable comparison across studies, confocal images with LAMP staining (as done in Figures 1 and 2) and accompanying quantification of lysosome size are needed to confirm that BafA1 and phloretin reduce endolysosomal size. Similarly, confocal imaging and quantification of lysosome size should also be done for sucrose treatment analysis currently shown in Fig. 3F.*
We now provide confocal immunofluorescence analysis of LAMP1 staining to show the impacts of BafA1 and phloretin on lysosome size (Fig. S3A). Follow up analysis of the effects of sucrose revealed that it is acting through a GABARAP-dependent mechanism that we had not anticipated in our original submission. Given that it is complicated to interpret and not necessary for support of the main claims of the revised manuscript, we no longer include this experiment in our manuscript.

*b)While sucrose treatment clearly leads to increased Rab10 phosphorylation, it is unclear whether it does so solely through its effects on endolysosomal size. High dose sucrose treatment can also induce lysosomal damage. To strengthen the authors' claims that this effect is due to endolysosomal swelling, assessment of lysosomal damage (e.g. imaging of galectin-3 levels on lysosomes following sucrose treatment) would be helpful to add confidence that this effect on Rab phosphorylation is likely due to changes in endolysosomal size rather than membrane integrity issues. In general, additional data is needed under the conditions used to add confidence that the effects observed are due to changes in lysosome swelling and not increased LMP.*
We appreciate these suggestions as our follow up revealed that the situation is more complex than we had originally suspected. As a result of the new data showing that the

effects of sucrose are GABARAP-dependent and an overall difficulty in fully pinning down the contribution of lysosome swelling, we no longer include this interpretation.

*4)Physiological relevance and scientific advance:*
*a)The authors propose an elegant mechanism by which LRRK2 can be activated in response to several different types of endolysosomal dysfunction. However, the physiological relevance of PIKFYVE inhibition and osmotic swelling with respect to PD remains unclear. Additional text should be added to the discussion that explores potential reasons why LRRK2 activation might be helpful in sensing and responding to endolysosomal swelling. While the potential benefits for repairing or removing damaged lysosomes is clear, it is not as clear why LRRK2 might be activated in response to endolysosomal swelling and what the downstream consequences of LRRK2 activation on addressing/correcting endolysosomal enlargement might be.*

In revising the manuscript, we have focused on defining the mechanism of LRRK2 activation at lysosomes rather than the downstream consequences of LRRK2 activity. We used acute pharmacological perturbations, including LLOME, apilimod, and nigericin, as tools to robustly induce distinct lysosomal stresses and determine how LRRK2 is recruited and activated at stressed lysosomes.

We agree that the physiological relevance of PIKfyve inhibition and osmotic swelling should be discussed more clearly. We have therefore revised the Discussion to clarify that apilimod is used here as a tool to model endolysosomal swelling, ion imbalance, and altered membrane homeostasis, rather than as a direct physiological PD trigger. We now discuss how the accumulation of multiple Rabs on stressed lysosomes may reflect broader remodeling of the lysosomal surface and recruit effectors involved in lysosome repair, remodeling, or damage signaling. We further propose that transient LRRK2 activation at stressed lysosomes may support adaptive membrane remodeling and restoration of lysosome function, whereas chronic perturbations of lysosomal Rab abundance, ionic or osmotic homeostasis, or membrane integrity could promote persistent LRRK2 activation in Parkinson’s disease-relevant settings.

*b)The novelty of the work should be further articulated in the discussion, as Rab-dependent recruitment of LRRK2 to dysfunctional lysosomes has already been established under many conditions (already well-cited in the text) and apilimod has been shown previously to lead to LRRK2 activation.*

We have updated the discussion to more clearly articulate that an important advance of this study is to place previously described LRRK2 recruitment mechanisms into a stress-specific framework. Prior studies established that Rab GTPases can interact with and activate LRRK2 and that apilimod increases LRRK2 pathway activity. Our findings integrate and extend this work by defining how different lysosomal perturbations engage distinct upstream mechanisms that converge on LRRK2. LRRK2 does not simply respond to a generic “dysfunctional lysosome” signal. Instead, its activation reflects integration of stress-specific Rab and GABARAP inputs that report on distinct aspects of

lysosome physiology. This is important both for understanding lysosome cell biology and potential contributions of lysosome dysfunction to Parkinson’s disease via LRRK2 dysregulation.

*Reviewer #3 (Comments to the Authors (Required)):*

*Summary:*

*In the manuscript "LRRK2 integrates Rab and GABARAP interactions to sense and respond to*
*distinct lysosomal stresses", Clegg et al., find a role for the Cl-/H+ exchanger, ClC7, in LRRK2 activation upon pharmacological inhibition of PIKfyve activity. With this finding, they distinguish ClC7-mediated LRRK2 activation from LRRK2 activity mediated by CASM-related GABARAP lipidation induced by various stimuli. While the role of ClC7 and the distinct requirements of ClC7 vs GABARAP in LRRK2 activation with different stimuli is novel and interesting, these data are far too preliminary and require further interrogation into the mechanisms of how inhibiting PIKfyve activity and ClC7 induce LRRK2 activation. The remaining half of the manuscript that focuses on Rab-dependent activation of LRRK2 confirms prior literature (Dhekne et al., 2023, Vides et al., 2022, Zhu et al., 2023, Wang et al, 2023), but does not add to our understanding of the mechanism of Rab-mediated LRRK2 activation, putting the novelty of this data into question. Lastly, the claims of LRRK2 activation downstream of osmotic "swelling" and the distinction of deacidification in terms of CASM-related GABARAP lipidation are confusing, inconsistent with the literature, and need major revision.*

In addressing the specific comments, we have included new data and have edited the manuscript to better communicate that the novelty and utility lies in demonstrating how responses to lysosome stresses are integrated by LRRK2 via its interactions with Rabs and GABARAP. Previous studies focused on either individual Rabs or GABARAP or responses to lysosome stresses as factors that contribute to LRRK2 activity but left open questions about how they are all integrated upstream of LRRK2. We have sought to make this point more clearly in the revised manuscript.

*Major Comments:*

*1. It's not clear how LRRK2 integrates multiple upstream cues through "parallel interactions" with Rab GTPases and GABARAP - GABARAP is required for LRRK2 activation with certain stimuli per the authors' prior publication (Bentley-DeSousa et al., 2025), but is GABARAP required for Rab recruitment? Are Rabs required for GABARAP lipidation? Experimentally answering these questions is necessary to determine whether their interactions with LRRK2 are "parallel", otherwise claims need to be revised.*

We appreciate the reviewer’s concern and agree that the term “parallel interactions” could imply a more complete epistasis analysis than we performed. In the revised manuscript, we show that perturbations that block CASM–GABARAP-dependent

LRRK2 activation (such as ATG16L1 KO, SopF expression, and GABARAP KO) do not robustly block LRRK2 activation by apilimod (Fig. 4C-H). In contrast, mutation of three established Rab-binding sites in LRRK2 blocks activation by multiple stimuli (Fig. 3A-C, 4I-L). Together with the lack of detectable GABARAP lipidation after apilimod treatment (Fig. 5C-D), these data support a model in which Rab interactions are broadly required for LRRK2 activation, whereas GABARAP is selectively required for LRRK2 responses CASM-inducing lysosomal stresses.

We have revised the disucssion to describe Rab and GABARAP as stimulus-selective inputs that converge on LRRK2 activation, rather than as strictly parallel pathways.

*2. The data regarding Rab recruitment is intriguing - more analysis of what is required for Rab recruitment, with support of imaging data, would strengthen our understanding of this upstream step of LRRK2 activation - Is ClC7 required for Rab recruitment? Does phloretin and BafA1 block Rab recruitment?*

In Figure 5 A and B, we provide new data showing that ClC-7 is only required for the apilimod-dependent recruitment of specific Rabs (5, 9, 12 and 32). Of these, Rabs 12 and 32 are well established binding partners, and activators, of LRRK2. Rab32 is of particular interest due to recent studies showing that it is a familial Parkinon's disease gene. We have updated the discussion to better highlight the relevance of these points. The new versions of Figures 3 F and G also provide additional Rab analysis and quantification.

*3. Eguchi et al., 2018 found that LRRK2 is not recruited to lysosomes upon Vacuolin-1 treatment, can the authors address this discrepancy with imaging of LRRK2 recruitment with Apilimod treatment?*

We appreciate the reviewer's point. Eguchi et al. reported that LRRK2 was not detectably recruited to lysosomes after vacuolin-1 treatment, whereas our data support lysosomal recruitment and signaling by LRRK2 following PIKfyve inhibition with apilimod, a more potent and robust PIKfyve inhibitor and LRRK2 activator (Fig. S1C-D). Direct immunofluorescent detection of endogenous LRRK2 at lysosomes is technically challenging, likely due to limitations of available antibodies and the relatively modest abundance of LRRK2. We therefore used complementary approaches to assess the site of LRRK2 action.

First, immunofluorescence analysis of phospho-Rab10, a direct LRRK2 substrate, shows enrichment at a subset of lysosomes after apilimod treatment, without obvious enrichment on other organelles (Fig. 1L and M). Because one LRRK2 molecule can phosphorylate many Rab molecules, and because the phospho-Rab10 antibody provides more robust detection than endogenous LRRK2 antibodies, phospho-Rab10 provides a sensitive readout of where LRRK2 has been active. Second, immunoblot analysis of purified lysosomes shows increased LRRK2 abundance on lysosomes after apilimod treatment (Fig. 1G-H; Fig. 2E-F; Figure 3D-E). Together, these imaging and biochemical data support lysosomes as a major site of LRRK2 activation following

PIKfyve inhibition.

*4. It is not clear how "lysosomal swelling is sufficient to activate LRRK2" based on the data presented. Lysosomal swelling/enlargement is regulated by solute and water carriers (PMID: 36219209), as well as lipid transfer proteins (Yang et al, 2025), and various other mechanisms. Any of these molecular events could be required for LRRK2 activation independently or in correlation with swelling. It would be technically difficult to dissect these events and their contributions to LRRK2 activation in a complex cellular system and would require a simplified in vitro system to rigorously test sufficiency. The authors should revise the claims regarding swelling to acknowledge these limitations.*

*The authors appear to come to this conclusion as they show sucrose-mediated lysosomal swelling and LRRK2 activation does not require ClC7 (Fig. 3F-H), however an alternative explanation could be that the mechanism of swelling and/or LRRK2 activation with sucrose is just not regulated by ClC7. The authors show that ClC7 is not required for LRRK2 activation by other stimuli that induce lysosomal swelling (Nigericin PMID: 40372734 and LLOMe), which raises the question of whether CASM-mediated GABARAP is induced by sucrose stress and required for LRRK2 activation, rather than ClC7? Additionally, lysosome enlargement induced by Apilimod is reduced in the ClC7KO (Fig. 2B), but it does not appear to be completely blocked in the representative image, which would be consistent with data in Leray et al., 2022. Quantification of lysosome size would help determine whether and to what extent swelling occurs in the ClC7KO. If there still is some lysosomal swelling in the ClC7KO, in which LRRK2 activation does not occur, then lysosomal swelling would not be sufficient.*

Upon careful consideration of these factors and new data that we have included in the revised manuscript (including quantification of lysosome size in ClC-7 KO +/- apilimod, Figure 2 A-B), we have decided to reduce emphasis on lysosome swelling as a direct activator of LRRK2 and have instead focused more on the roles of GABARAP and Rabs as integrators of lysosome stress upstream of LRRK2.

We agree with the reviewer that our original wording overstated the conclusion that lysosomal swelling is sufficient to activate LRRK2. Lysosome enlargement is a complex phenotype that can reflect changes in ion flux, solute accumulation, water transport, membrane trafficking, and lipid transfer. We therefore cannot conclude from cell-based experiments alone that swelling itself is the direct activating signal.

In the revised manuscript, we have tempered this claim and now describe lysosome swelling/enlargement as a stress-associated state linked to LRRK2 activation. We have also added quantification of lysosome size in ClC-7 KO cells with and without apilimod (Fig. 2A-B). Finally, we have shifted the emphasis toward our better-supported conclusion that Rab and GABARAP interactions allow LRRK2 to integrate distinct lysosomal stress states.

*5. Similarly, the distinction that is made between manipulations that induce lysosome enlargement/swelling and deacidification, as if CASM-mediated GABARAP lipidation is*

*only related to deacidification, is confusing and inconsistent with the literature. Stimuli that induce CASM-mediated ATG8 lipidation, such as LLOMe, Nigericin, and Monensin, also induce enlargement/swelling of lysosomes, and TRPML1 agonists that do not deacidify lysosomes, but rather hyperacidify (PMID: 39433126, PMID: 32858116), like PIKfyve inhibition (Leray et al., 2022) induce CASM and related LRRK2 activation, as the authors have previously shown (Bentley-DeSousa et al., 2025). Rather, it appears from the data that stimuli causing ionic/osmotic imbalances and lysosomal swelling also induce LRRK2 activation through different mechanisms, requiring either ClC7 or GABARAP lipidation. Please revise and clarify the claims around this distinction.*

*With these considerations, I am wondering whether PIKfyve inhibition induces CASM-mediated GABARAP lipidation? And, whether ClC7 regulates swelling with Nigericin and LLOMe? Answering these questions would help further dissect the differences of these 2 pathways mediating LRRK2 activation.*

We appreciate the reviewer's concern and agree that our original framing created an overly sharp distinction between lysosome swelling/enlargement and deacidification. These are overlapping consequences of many lysosomal perturbations, and CASM-inducing stimuli can also cause lysosome enlargement. We have therefore revised the manuscript to avoid presenting swelling and deacidification as mutually exclusive upstream mechanisms.

Instead, we now frame the key distinction in terms of CASM–GABARAP dependence. Under our experimental conditions, LLOME, nigericin, and related CASM-inducing stresses require GABARAP for robust LRRK2 activation, whereas PIKfyve inhibition activates LRRK2 through a ClC-7- and Rab-dependent pathway that is not robustly impaired by ATG16L1 KO, SopF expression, or GABARAP KO. Consistent with this distinction, we do not detect substantial GABARAP lipidation after apilimod treatment compared with LLOME or nigericin (Fig, 5C-D).

We agree that further dissecting how ClC-7, lysosome size, ion imbalance, and CASM activation intersect across different stimuli would be valuable. However, a complete analysis of the upstream mechanisms that trigger CASM or regulate lysosome swelling is beyond the scope of this study. We have revised the text to make clear that our main conclusion is that distinct lysosomal stresses converge on LRRK2 through different requirements for Rab and GABARAP interactions, rather than through a simple swelling-versus-deacidification dichotomy.

*6. The V-ATPase is also reported to be regulated by PIKfyve/Fab1 (PMID: 27335171, PMID: 30730983, PMID: 29791245, PMID: 32656214). The authors demonstrate that swelling and LRRK2 activation induced by Apilimod are blocked by BafA1 (Fig. 3A-C) - is the V-ATPase contributing to swelling and LRRK2 activation by PIKfyve inhibition or does BafA1 just deplete the available protons for ClC7 activity?*

We agree that BafA1 does not distinguish whether V-ATPase activity contributes directly to apilimod-induced lysosome swelling and LRRK2 activation, or indirectly by maintaining the proton gradient required for ClC-7-mediated chloride/proton exchange.

Given the strong requirement for ClC-7 in our experiments, the dependence of ClC-7 function on the lysosomal proton gradient but also the role for Cl- as a counterion that supports lysosome acidification, we cannot cleanly separate these possibilities in the current study. We have therefore revised the manuscript to interpret the BafA1 experiment more cautiously. We now state that V-ATPase activity is required for apilimod-induced lysosome enlargement and LRRK2 activation, consistent with a ClC-7- and proton-gradient-dependent lysosomal stress pathway, while noting that additional V-ATPase-dependent effects cannot be excluded.

A detailed dissection of how PIKfyve, V-ATPase, and ClC-7 are coupled is beyond the scope of this study and would distract from our main focus on LRRK2 regulatory mechanisms.

*7. Clcn7 KO validation (Fig. S2A) is insufficient - it is not clear how the PCR analysis used can distinguish between a frame shifting or in-frame deletion. The authors need to show protein level KO of ClC7, or at least genetic and transcript level analysis to validate the KO if there is not a good commercially available antibody. Please also include the guide RNA sequence, exon target information, and primer sequences for PCR validation used for KO cell lines in the methods for proper reporting.*

We were unable to identify a validated antibody suitable for reliable detection of endogenous mouse ClC-7 by immunoblotting despite searching the literature and consulting with a colleague (Joe Mindell, NIH) with expertise in ClC-7 biology. We therefore relied on sequence-level genetic validation. Our KO strategy used co-transfection of three Cas9 RNPs targeting sites near exon 5 of Clcn7, with the goal of generating large deletions rather than small indels. Sequence analysis of the two independent clones used in the study confirmed disruptive deletions at the Clcn7 locus. Clone 1 contained 129 bp and 134 bp deletions; these deletions extended from within exon 5 into the downstream intron and removed 62 bp of coding sequence. Clone 2 contained a 133 bp deletion that removed all 71 bp of exon 5 together with adjacent flanking intronic sequence. These deletions all disrupt the Clcn7 coding region and support the use of these clones as Clcn7 KO lines.
We have updated our explanation for the validation of the ClC-7 KO clones and updated the Methods and Key Resources Table to include the guide RNA sequences, exon target information, genotyping primer sequences, and details of the PCR/sequencing strategy.

*8. The discussion reads more like a general review of the literature and requires more discussion of the conclusions that can be drawn from the results of the manuscript within that context.*

We have updated the discussion to better highlight our key findings.

*Minor Comments:*

*1. Authors claim to test "biophysical" approaches, however it is unclear what biophysical approaches were used. If the authors mean to suggest the sucrose-mediated experiments are biophysical, sucrose-mediated swelling is not purely biophysical, and is also driven by osmotic regulation by aquaporins (Yang et al., 2025), and likely other solute carriers. The authors should revise this language to not confuse the reader.*

We have updated the text to limit the use of the term “biophysical”.

*2. Figure legends for Fig S4 do not match the panel labels in the figure. Please revise and ensure consistency in other figure legends.*

Thanks for catching this. We have corrected the revised manuscript.

*3. The plotted data in figures appears to be normalized, but normalization methods are not reported in the methods or figure legends. Please add whether and how normalization methods were used, and whether the statistical analyses were done on normalized or raw values.*

We have provided this information in the updated methods section.

*4. Please use standard deviation or confidence intervals for error bars, rather than SEM, to show variability in the data set transparently (PMID: 31145670).*

We appreciate the reviewer’s concern regarding transparent display of variability. In line with JCB requirements, we show individual data points from independent experiments, which directly display the spread of the measurements. We use SEM only to indicate the precision of the estimated mean. We have also ensured that each legend reports the number of independent experiments and statistical test. These data presentation choices provide the reader with essential information for evaluating our data and are in line with standards in the field.

*5. What is Pan-pRab on the figure 5H Y axis? Does not match figure legend.*

We apologize for the confusion. The commercially available phospho-Rab8a antibody that we used (abcam:ab_230260) has been characterized to cross-react with other phospho-Rab substrates of LRRK2 (Lis et al, 2018). We have updated the manuscript to better explain this.

*6. It is stated in the significance statement that "Osmotic swelling drives strong Rab-dependent activation, whereas deacidification requires CASM-mediated GABARAP lipidation as a scaffold for LRRK2 activation at lysosomes" makes it seem like Rabs are not required in conditions of deacidification, however the authors show that LRRK2-Rab*

*interactions are required with Nigericin and LLOMe in Fig 5G-H. Later it is correctly stated in the introduction that "LRRK2-Rab interactions are essential for activation by both swelling and CASM-dependent stimuli, identifying Rabs as a unifying hub through which diverse lysosomal stress pathways converge on LRRK2". Please revise for consistency.*

We see how the original wording in the Significance Statement could incorrectly imply that Rab interactions are dispensable during CASM-inducing stresses. We have revised the Significance Statement to clarify that Rab interactions are broadly required for LRRK2 activation across the stimuli tested, whereas GABARAP provides an additional scaffold specifically under CASM-inducing conditions. This revised wording is now consistent with the Introduction and with our data showing that LRRK2–Rab interactions are required for activation by LLOME and nigericin.

*7. Rab accumulation does not appear to be tested in the section titled "Lysosomal swelling drives Rab accumulation and LRRK2 activation". Please revise.*

Thanks for catching this. We have addressed this issue in the revised manuscript.

August 12, 2026

RE: JCB Manuscript #202511137R

Shawn Ferguson
Yale University

Dear Dr. Ferguson; Dear Shawn,

Thank you for submitting your revised manuscript entitled "LRRK2 integrates Rab and GABARAP interactions to respond to distinct lysosomal stresses". Your study has been reassessed by the original reviewers who appreciate your experimental revisions and are supportive of publication pending some final revisions to temper and clarify your conclusions. Therefore, we would be happy to publish your paper in JCB pending final revisions necessary to meet our formatting guidelines (see details below) along with completely addressing reviewer 1 and 3's final comments.

To avoid unnecessary delays in the acceptance and publication of your paper, please read the following information carefully.

A. MANUSCRIPT ORGANIZATION AND FORMATTING:

Full guidelines are available on our Instructions for Authors page, https://jcb.rupress.org/submission-guidelines#revised.
**Submission of a paper that does not conform to JCB guidelines will delay the acceptance of your manuscript.**

1) Text limits: Character count for Reports is < 20,000, not including spaces. Count includes abstract, introduction, * combined results and discussion, and acknowledgments. Count does not include title page, figure legends, materials and methods, references, tables, or supplemental legends.

2) Figures limits: Reports may have up to 5 main text figures.

3) Figure formatting: Scale bars must be present on all microscopy images, including inset magnifications. Molecular weight or nucleic acid size markers must be included on all gel electrophoresis. Aspect ratios of images may not be altered.

4) Statistical analysis: Error bars on graphic representations of numerical data must be clearly described in the figure legend. The number of independent data points (n) represented in a graph must be indicated in the legend. Statistical methods should be explained in full in the materials and methods. For figures presenting pooled data the statistical measure should be defined in the figure legends. Please also be sure to indicate the statistical tests used in each of your experiments (either in the figure legend itself or in a separate methods section) as well as the parameters of the test (for example, if you ran a t-test, please indicate if it was one- or two-sided, etc.). Also, if you used parametric tests, please indicate if the data distribution was tested for normality (and if so, how). If not, you must state something to the effect that "Data distribution was assumed to be normal but this was not formally tested."

5) Abstract and title: The abstract should be no longer than 160 words and should communicate the significance of the paper for a general audience. The title should be less than 100 characters including spaces. Make the title concise but accessible to a general readership.

6) Materials and methods: Should be comprehensive and not simply reference a previous publication for details on how an experiment was performed. Please provide full descriptions in the text for readers who may not have access to referenced manuscripts.

7) All antibodies, cell lines, animals, and tools used in the manuscript should be described in full, including accession numbers for materials available in a public repository such as the Resource Identification Portal. Please be sure to provide the sequences for all of your primers/oligos and RNAi constructs in the materials and methods. You must also indicate in the methods the source, species, and catalog numbers (where appropriate) for all of your antibodies. Please also indicate the acquisition and quantification methods for immunoblotting/western blots.

8) Microscope image acquisition: The following information must be provided about the acquisition and processing of images:
a. Make and model of microscope
b. Type, magnification, and numerical aperture of the objective lenses
c. Temperature
d. Imaging medium
e. Fluorochromes
f. Camera make and model
g. Acquisition software

h. Any software used for image processing subsequent to data acquisition. Please include details and types of operations involved (e.g., type of deconvolution, 3D reconstitutions, surface or volume rendering, gamma adjustments, etc.).

9) References: There is no limit to the number of references cited in a manuscript. References should be cited parenthetically in the text by author and year of publication. Abbreviate the names of journals according to PubMed.

10) * Supplemental materials: There are strict limits on the allowable amount of supplemental data. Reports may have up to 3 supplemental figures. Therefore, please reduce your SI count and be sure to correct the callouts in the text to reflect all changes. Please also note that tables, like figures, should be provided as individual, editable files. A summary of all supplemental material should appear at the end of the Materials and methods section.

11) eTOC summary: A ~40-50-word summary that describes the context and significance of the findings for a general readership should be included on the title page. The statement should be written in the present tense and refer to the work in the third person.

12) Conflict of interest statement: JCB requires inclusion of a statement in the acknowledgements regarding competing financial interests. If no competing financial interests exist, please include the following statement: "The authors declare no competing financial interests." If competing interests are declared, please follow your statement of these competing interests with the following statement: "The authors declare no further competing financial interests."

13) ORCID IDs: ORCID IDs are unique identifiers allowing researchers to create a record of their various scholarly contributions in a single place. Please note that ORCID IDs are now *required* for all authors. At resubmission of your final files, please be sure to provide your ORCID ID and those of all co-authors.

14) A separate author contribution section following the Acknowledgments. All authors should be mentioned and designated by their full names. We encourage use of the CRediT nomenclature.

Please note that JCB now requires authors to submit Source Data used to generate figures containing gels and Western blots with all revised manuscripts. This Source Data consists of fully uncropped and unprocessed images for each gel/blot displayed in the main and supplemental figures. For assays performed using capillary electrophoresis and/or immunoassay-based detection, authors should instead provide the electropherogram graph(s) for each experiment, plotting fluorescence/chemiluminescence intensity vs. molecular weight/size. Please be sure to provide one Source Data file for each figure gels, blots, and/or capillary electrophoresis assays along with your revised manuscript files. File names for Source Data figures should be alphanumeric without any spaces or special characters (i.e., SourceDataF#, where F# refers to the associated main figure number or SourceDataFS# for those associated with Supplementary figures). For traditional gels and blots, the lanes of the gels/blots should be labeled as they are in the associated figure, the place where cropping was applied should be marked (with a box), and molecular weight/size standards should be labeled wherever possible. For capillary electrophoresis assays, each trace in the graph should be color-coded and labeled to indicate which protein, gene, or sample is being measured (please try to avoid red/green combinations to accommodate our color-blind readers).

Source Data files will be made available to reviewers during evaluation of revised manuscripts and, if your paper is eventually published in JCB, the files will be directly linked to specific figures in the published article.

Source Data Figures should be provided as individual PDF files (one file per figure). Authors should endeavor to retain a minimum resolution of 300 dpi or pixels per inch. Please review our instructions for export from Photoshop, Illustrator, and PowerPoint here: https://rupress.org/jcb/pages/submission-guidelines#revised

Journal of Cell Biology now requires a data availability statement for all research article submissions. These statements will be published in the article directly above the Acknowledgments. The statement should address all data underlying the research presented in the manuscript. Please visit the JCB instructions for authors for guidelines and examples of statements at (https://rupress.org/jcb/pages/editorial-policies#data-availability-statement).

B. FINAL FILES:

Please upload the following materials to our online submission system. These items are required prior to acceptance. If you have any questions, contact JCB's Managing Editor, Lindsey Hollander (lhollander@rockefeller.edu).

-- An editable version of the final text (.DOC or .DOCX) is needed for copyediting (no PDFs).

-- High-resolution figure and MP4 video files: See our detailed guidelines for preparing your production-ready images, https://jcb.rupress.org/fig-vid-guidelines.

-- Cover images: If you have any striking images related to this story, we would be happy to consider them for inclusion on the journal cover. Submitted images may also be chosen for highlighting on the journal table of contents or JCB homepage carousel.

Images should be uploaded as TIFF or EPS files and must be at least 300 dpi resolution.

**It is JCB policy that if requested, original data images must be made available to the editors. Failure to provide original images upon request will result in unavoidable delays in publication. Please ensure that you have access to all original data images prior to final submission.**

**The license to publish form must be signed before your manuscript can be sent to production. A link to the license to publish form will be sent to the corresponding author only. Please take a moment to check your funder requirements before choosing the appropriate license.**

Additionally, JCB encourages authors to submit a short video summary of their work. These videos are intended to convey the main messages of the study to a non-specialist, scientific audience. Think of them as an extended version of your abstract, or a short poster presentation. We encourage first authors to present the results to increase their visibility. The videos will be shared on social media to promote your work. For more detailed guidelines and tips on preparing your video, please visit https://rupress.org/jcb/pages/submission-guidelines#videoSummaries.

Thank you for your attention to these final processing requirements. Please revise and format the manuscript and upload materials within 14 days. If you need an extension for whatever reason, please let us know and we can work with you to determine a suitable revision period.

Please contact the journal office with any questions at cellbio@rockefeller.edu.

Thank you for this interesting contribution, we look forward to publishing your paper in Journal of Cell Biology.

Sincerely,

Mark von Zastrow
Monitoring Editor

Andrea L. Marat
Deputy Editor

Journal of Cell Biology

------------------------------------------------------------------------------
Reviewer #1 (Comments to the Authors (Required)):

The authors have tried to respond with great care to the concerns and the paper is improved and should be accepted with minor revision. However, the title and abstract do not convey the contents and need revising as follows:

Line 143 Change summary statement "Lysosomal enlargement drives" to PIKfyve inhibition drives or something similar

apilimod-induced lysosome enlargement (line 144) should be reworded to Apilomod-altered lysosomes since enlargement is no longer the relevant variable

This is an odd summary for a paper not discussing the CASM route. In the scenario presented in this manuscript, there is no coincidence detection. Please re-phrase to reflect what is new IN THIS PAPER.

Also, title doesn't match experiments shown here. Why not include ClC7 and PIKfyve in title? The abstract should reflect the experiments here not the conclusions since many authors only read abstracts; eTOC is better

This is not suitable for THIS paper: "Together, our results support a model in which LRRK2 acts as a coincidence detector for lysosomal stress, using Rab GTPases as broadly required inputs and GABARAP as a conditional scaffold under CASM-inducing conditions (Fig. 5G). This architecture may restrict LRRK2 activation to appropriately stressed lysosomes while limiting inappropriate signaling at other membranes"

Reviewer #2 (Comments to the Authors (Required)):

The authors have significantly improved their manuscript, addressing my major concerns around quantification of some of the imaging data, confirmation of successful CLC-7 KO generation, interpretation of sucrose findings, and tempering of some of the conclusions with respect to novelty of the work. I have no additional comments and feel this is appropriate for publication in JCB.

Reviewer #3 (Comments to the Authors (Required)):

The additional experimental evidence and revisions the authors have provided strengthens the insights in the manuscript. However, several claims remain overstated, and I am finding the new emphasis on CASM/GABARAPs and the "integration" of Rabs and GABARAPs in the recruitment and activation of LRRK2 in the text quite confusing in the context of the data presented. Most of the data focuses on the role of ClC7 (which is not even mentioned in the abstract), while only 4 experiments out of the whole manuscript report on GABARAP lipidation. Revisions are needed in the abstract, title, and text to clearly reflect the data reported in the manuscript. That being said, I think the data is quality and the insights are interesting, however further revisions are needed for focus and clarity in the text, so JCB readers can clearly understand the new data and claims within the context of prior literature.

Remaining specific comments:

1. Please revise the section titled "Lysosomal enlargement drives LRRK2 signaling". As noted in my prior comments, and acknowledged by the authors in the rebuttal, the evidence presented does not support lysosomal enlargement/swelling as a causal factor in LRRK2 signaling, which is implied by the use of the term "drives". The data presented in the section is correlative and the new data showing lysosomal enlargement in the Clc7KO and pRab10 localization does not support this conclusion.

2. Please revise claims regarding Rab32 recruitment in Fig. 5a-b. In Fig. 5a-b, the authors claim that Rab32 detection at lysosomes is "lost" [line 195]. The Rab32 trends downward in the KO, but it is not statistically significant. Please revise claims to accurately reflect the Rab32 data presented. Further, Rab "accumulation" implies a change in Rabs under untreated vs treated conditions, however the analysis presented is treated only KO/WT. It would be helpful to present the ratios of Rab detection on lysosomes between untreated and treated in WT and KO, as "accumulation" may still occur in the KO, just at lower levels compared to WT. Please clarify in the text.

3. Please revise claims around the order of operations in ClC7 activity and direct interactions with Rabs. In lines 99-102, authors state "We show that PIKfyve inhibition activates LRRK2 through a CASM-independent pathway that begins with ClC-7 activity and proceeds through the accumulation of multiple Rabs on enlarged lysosomes and direct LRRK2-Rab interactions but without a requirement for CASM and GABARAP."
- The authors do not present evidence that LRRK2 activation "begins" with ClC-7 activity - other factors could be involved upstream. Please revise.
- The authors also do not present evidence that there are "direct" LRRK2-Rab interactions. The evidence shown demonstrates that mutations in the regions known to interact with specific Rabs are required, which indirectly suggests Rab interactions are involved, but whether these interactions directly occur or are abolished with apilimod treatment is not tested (e.g., by immunoprecipitation). The Rab interacting regions are also located within larger domains that could interact with other factors, which the authors acknowledge [lines 252-254]. Whether loss of interacting Rabs also produces similar results would also need to be tested to bolster the claim for "direct" interactions.

4. There are also some general tense and grammatical errors that need revision in the text.

**Response to Reviewers**

----------------------------------------------------------------------------

*Reviewer #1 (Comments to the Authors (Required)):*

*The authors have tried to respond with great care to the concerns and the paper is improved and should be accepted with minor revision. However, the title and abstract do not convey the contents and need revising as follows:*

We appreciate the time taken to help us better focus our message. We have updated the Title and Abstract to better highlight our key findings related to PIKfyve and ClC-7.

*Line 143 Change summary statement "Lysosomal enlargement drives" to PIKfyve inhibition drives or something similar*

We have removed this statement.

*apilimod-induced lysosome enlargement (line 144) should be reworded to Apilomod-altered lysosomes since enlargement is no longer the relevant variable*

We reduced emphasis on interpretation of “lysosome enlargement” by removing the section title. This follow up suggestion does not fit as well as the idea that both the bafilomycin and phloretin treatments reduce lysosome enlargement builds on previous studies that we cited on this topic. As the text currently stands, we report the correlation between effects on apilimod-induced lysosome enlargement without declaring a causative relationship.

*This is an odd summary for a paper not discussing the CASM route. In the scenario presented in this manuscript, there is no coincidence detection. Please re-phrase to reflect what is new IN THIS PAPER.*

In merging the results and discussion, this summary was significantly edited. In revising the manuscript, we have been mindful of not over-emphasizing CASM. However, we feel that it is appropriate to note that our results show that in contrast to stimuli such as LLOME, nigericin and STING which engage CASM as part of their activation of LRRK2, apilimod does not. In keeping with other suggestions, the role for ClC-7 in this process is now better acknowledged.

*Also, title doesn't match experiments shown here. Why not include ClC7 and PIKfyve in title? The abstract should reflect the experiments here not the conclusions since many authors only read abstracts; eTOC is better*

We appreciate the suggestion and have updated the title accordingly. The new title is:
**ClC-7 links PIKfyve inhibition to Rab-dependent LRRK2 activity at lysosomes**

*This is not suitable for THIS paper: "Together, our results support a model in which LRRK2 acts as a coincidence detector for lysosomal stress, using Rab GTPases as broadly required inputs and GABARAP as a conditional scaffold under CASM-inducing conditions (Fig. 5G). This architecture may restrict LRRK2 activation to appropriately stressed lysosomes while limiting inappropriate signaling at other membranes"*

In the revised manuscript, we have updated this summary to better reflect our specific observations related to the PIKfyve- and ClC-7-dependent regulation of LRRK2.

*Reviewer #2 (Comments to the Authors (Required)):*

*The authors have significantly improved their manuscript, addressing my major concerns around quantification of some of the imaging data, confirmation of successful CLC-7 KO generation, interpretation of sucrose findings, and tempering of some of the conclusions with respect to novelty of the work. I have no additional comments and feel this is appropriate for publication in JCB.*

We appreciate the effort that went into evaluating our manuscript.

*Reviewer #3 (Comments to the Authors (Required)):*

*The additional experimental evidence and revisions the authors have provided strengthens the insights in the manuscript. However, several claims remain overstated, and I am finding the new emphasis on CASM/GABARAPs and the "integration" of Rabs and GABARAPs in the recruitment and activation of LRRK2 in the text quite confusing in the context of the data presented. Most of the data focuses on the role of ClC7 (which is not even mentioned in the abstract), while only 4 experiments out of the whole manuscript report on GABARAP lipidation. Revisions are needed in the abstract, title, and text to clearly reflect the data reported in the manuscript. That being said, I think the data is quality and the insights are interesting, however further revisions are needed for focus and clarity in the text, so JCB readers can clearly understand the new data and claims within the context of prior literature.*

We appreciate the suggestions and have made updates to the title, abstract and elsewhere in the text to better highlight the contributions of PIKfyve and ClC-7 to regulation of LRRK2.

Remaining specific comments:

*1. Please revise the section titled "Lysosomal enlargement drives LRRK2 signaling". As noted in my prior comments, and acknowledged by the authors in the rebuttal, the evidence presented does not support lysosomal enlargement/swelling as a causal factor in LRRK2 signaling, which is implied by the use of the term "drives". The data presented in the*

*section is correlative and the new data showing lysosomal enlargement in the Clc7KO and pRab10 localization does not support this conclusion.*

We have updated this section. This includes removing the title which was overly conclusive. However, we retain some mention of lysosome enlargement in the text in order to reflect the fact that these experiments were motivated by literature linking PIKfyve inhibition to lysosome enlargement and the use of bafilomycin and phloretin to prevent this enlargement. We have cited the relevant papers on this topic.

*2. Please revise claims regarding Rab32 recruitment in Fig. 5a-b. In Fig. 5a-b, the authors claim that Rab32 detection at lysosomes is "lost" [line 195]. The Rab32 trends downward in the KO, but it is not statistically significant. Please revise claims to accurately reflect the Rab32 data presented. Further, Rab "accumulation" implies a change in Rabs under untreated vs treated conditions, however the analysis presented is treated only KO/WT. It would be helpful to present the ratios of Rab detection on lysosomes between untreated and treated in WT and KO, as "accumulation" may still occur in the KO, just at lower levels compared to WT. Please clarify in the text.*

We have moderated conclusions related to Rab32. We have furthermore performed new experiments that used Rab32 siRNA to directly test its role in the regulation of LRRK2 by PIKfyve inhibition. The results of these experiments argue against a critical role for Rab32 in this LRRK2 regulation and we have adjusted our interpretation accordingly.

We opted to focus analysis of lysosomal Rab accumulation on KO/WT ratio in order to simplify the comparisons and thus better highlight the most striking differences. This helps to best explain why Rab12 and 32 were prioritized for follow up. The new evidence that we provide for Rab12 in supporting LRRK2 signaling in apilimod-treated cells supports this strategy.

*3. Please revise claims around the order of operations in ClC7 activity and direct interactions with Rabs. In lines 99-102, authors state "We show that PIKfyve inhibition activates LRRK2 through a CASM-independent pathway that begins with ClC-7 activity and proceeds through the accumulation of multiple Rabs on enlarged lysosomes and direct LRRK2-Rab interactions but without a requirement for CASM and GABARAP."*
*- The authors do not present evidence that LRRK2 activation "begins" with ClC-7 activity - other factors could be involved upstream. Please revise.*
*- The authors also do not present evidence that there are "direct" LRRK2-Rab interactions. The evidence shown demonstrates that mutations in the regions known to interact with specific Rabs are required, which indirectly suggests Rab interactions are involved, but whether these interactions directly occur or are abolished with apilimod treatment is not tested (e.g., by immunoprecipitation). The Rab interacting regions are also located within larger domains that could interact with other factors, which the authors acknowledge [lines 252-254]. Whether loss of interacting Rabs also produces similar results would also need to be tested to bolster the claim for "direct" interactions.*

We thank the reviewer for identifying these points. We agree that our original wording implied a more strictly defined sequence of events than is established by our experiments. We have therefore revised the manuscript to state that ClC-7 is required for, rather than necessarily the initiating event in, PIKfyve-inhibition-induced LRRK2 activation. We have avoided statements that our experiments demonstrate “direct LRRK2–Rab interactions” with language specifying that established Rab-binding surfaces within LRRK2 are required for this response. Previous biochemical and structural studies demonstrated direct binding of Rabs to these regions, but our experiments do not directly measure these interactions following PIKfyve inhibition.

In addition, prompted by the reviewer’s suggestion to test individual LRRK2-interacting Rabs, we have now examined Rab12 and find that loss of Rab12 reduces PIKfyve-inhibition-induced LRRK2 signaling. These new data identify Rab12 as a specific contributor to this pathway and have been incorporated into the revised manuscript as Fig. 5 C and D. We have revised our interpretation accordingly and now describe a pathway in which PIKfyve inhibition produces ClC-7-dependent changes in lysosomal Rab abundance that promote Rab-dependent LRRK2 activation, while acknowledging that additional factors may contribute.

*4. There are also some general tense and grammatical errors that need revision in the text.*

We are grateful for this attention to detail. We have read through the manuscript and have done our best to correct these errors.
