# Supplementary material for: ClC-7 links PIKfyve inhibition to Rab-dependent LRRK2 activity at lysosomes
Source: J Cell Biol. 2026 Oct 20;225(11):e202511137. doi: 10.1083/jcb.202511137 (PMC13682606; doi:10.1083/jcb.202511137)

1A

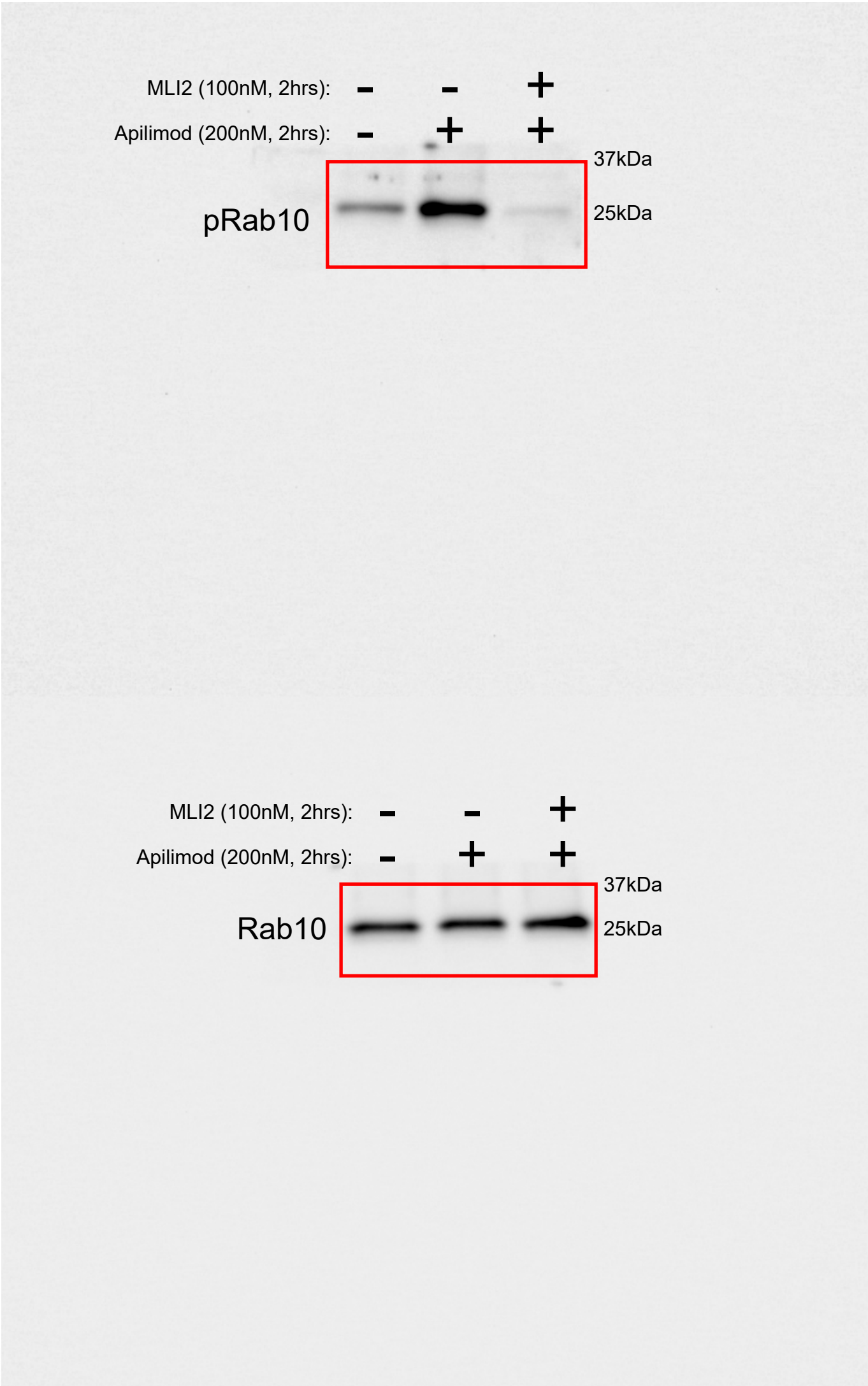

MLI2 (100nM, 2hrs): - - +
Apilimod (200nM, 2hrs): - + +
37kDa
pRab10
25kDa
MLI2 (100nM, 2hrs): - - +
Apilimod (200nM, 2hrs): - + +
37kDa
Rab10
25kDa

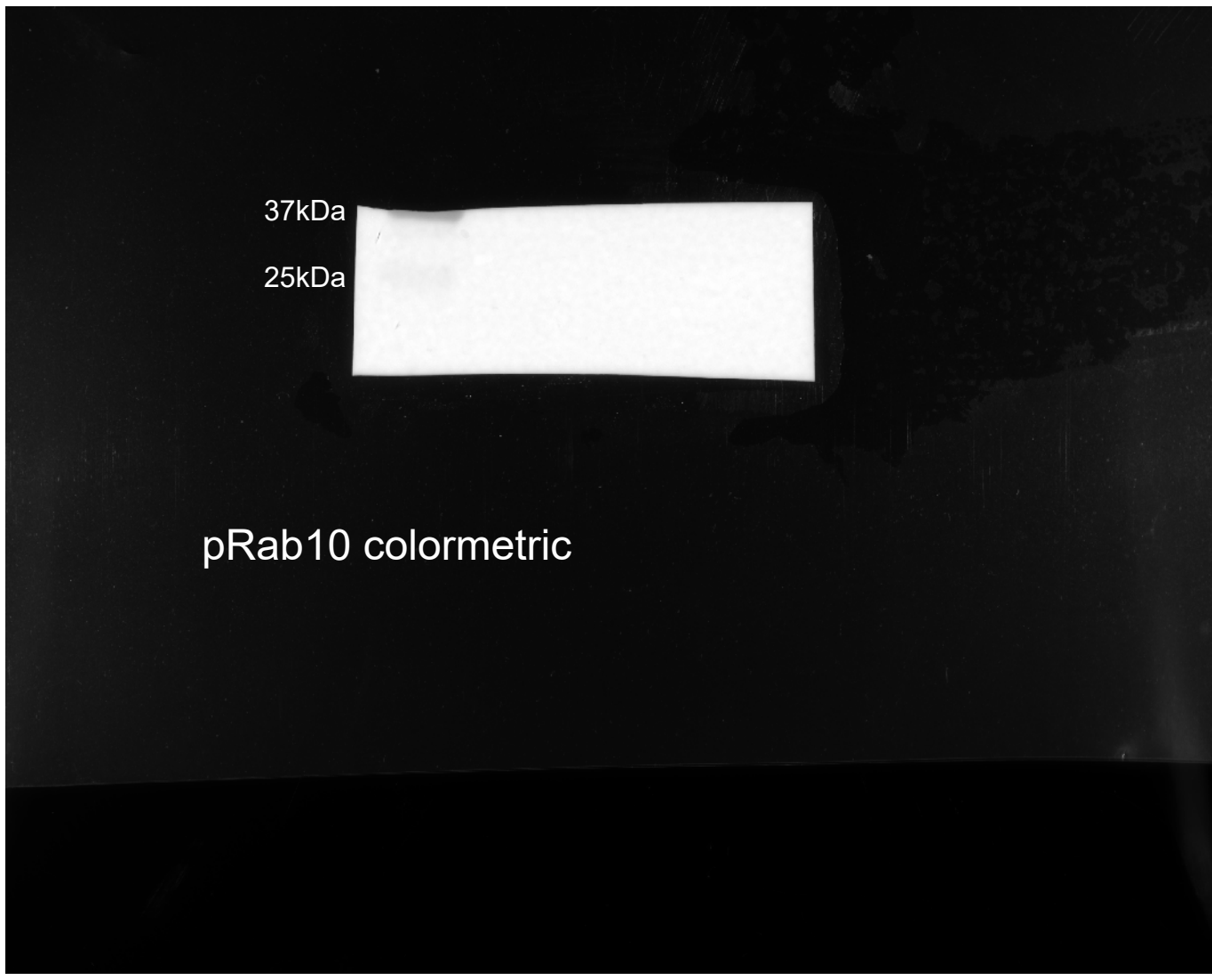

37kDa
25kDa
pRab10 colormetric

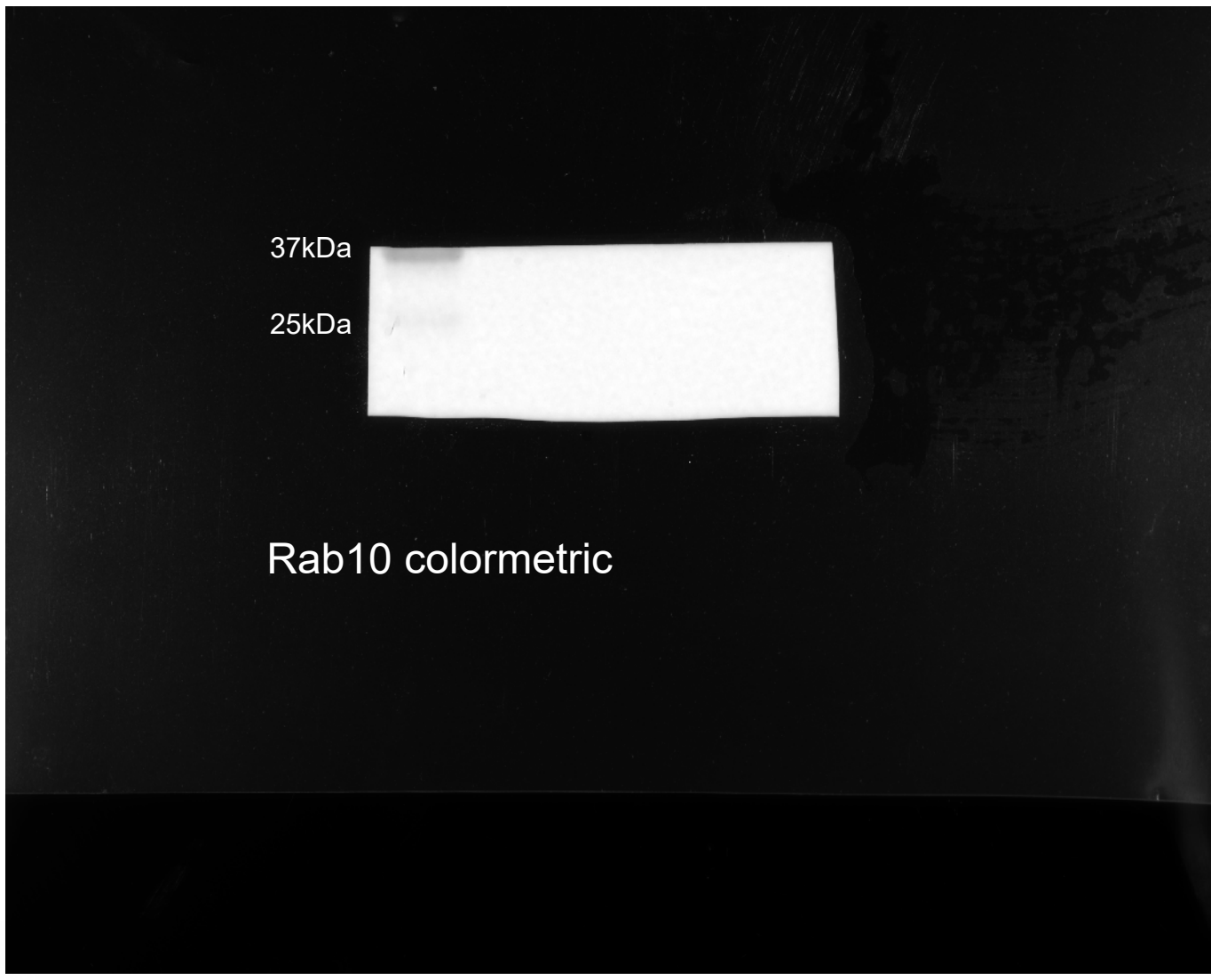

37kDa
25kDa
Rab10 colormetric

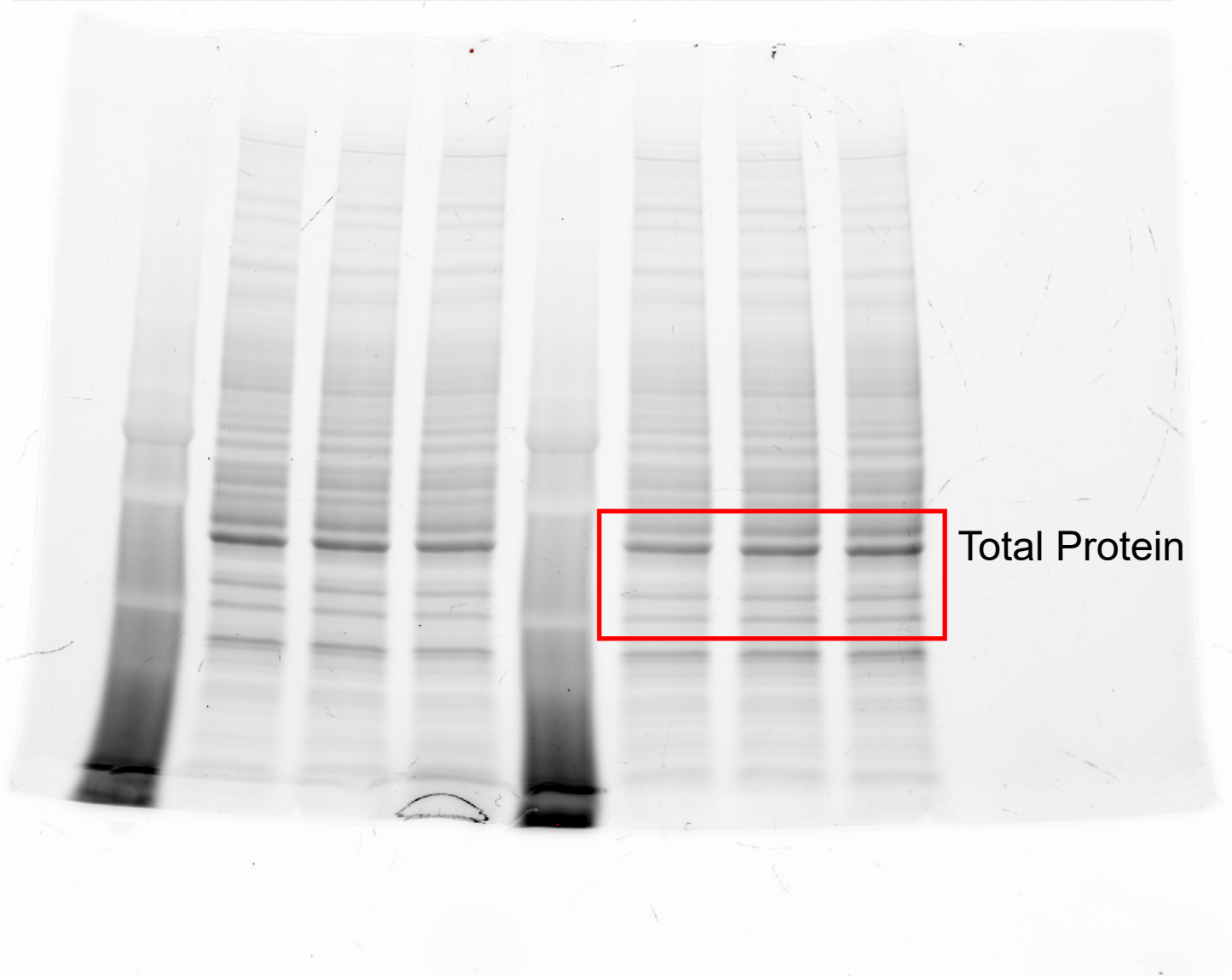

Total Protein

1E

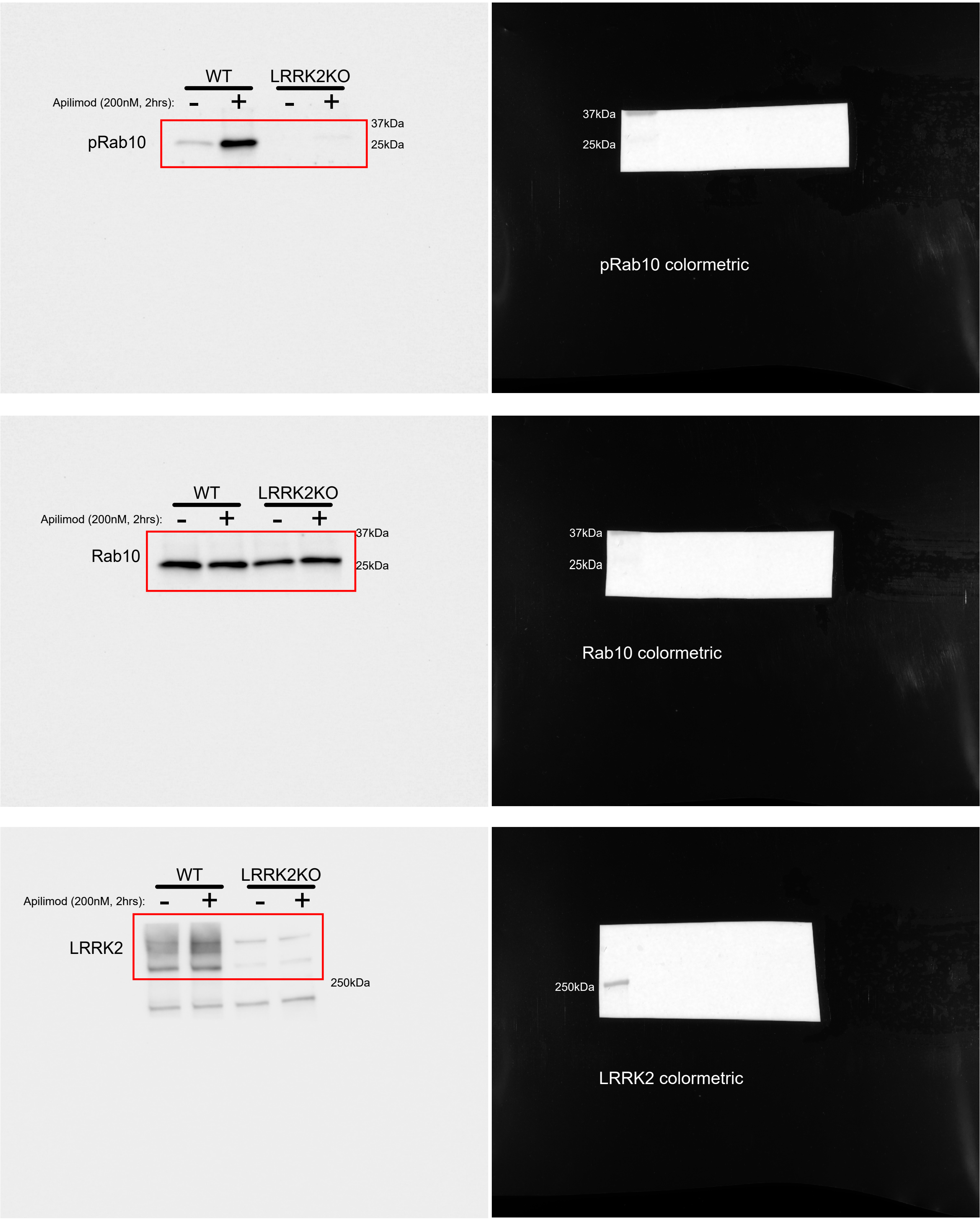
WT
LRRK2KO
Apilimod (200nM, 2hrs): - + - +
pRab10
37kDa
25kDa
37kDa
25kDa
pRab10 colormetric
WT
LRRK2KO
Apilimod (200nM, 2hrs): - + - +
Rab10
37kDa
25kDa
37kDa
25kDa
Rab10 colormetric
WT
LRRK2KO
Apilimod (200nM, 2hrs): - + - +
LRRK2
250kDa
250kDa
LRRK2 colormetric

1G

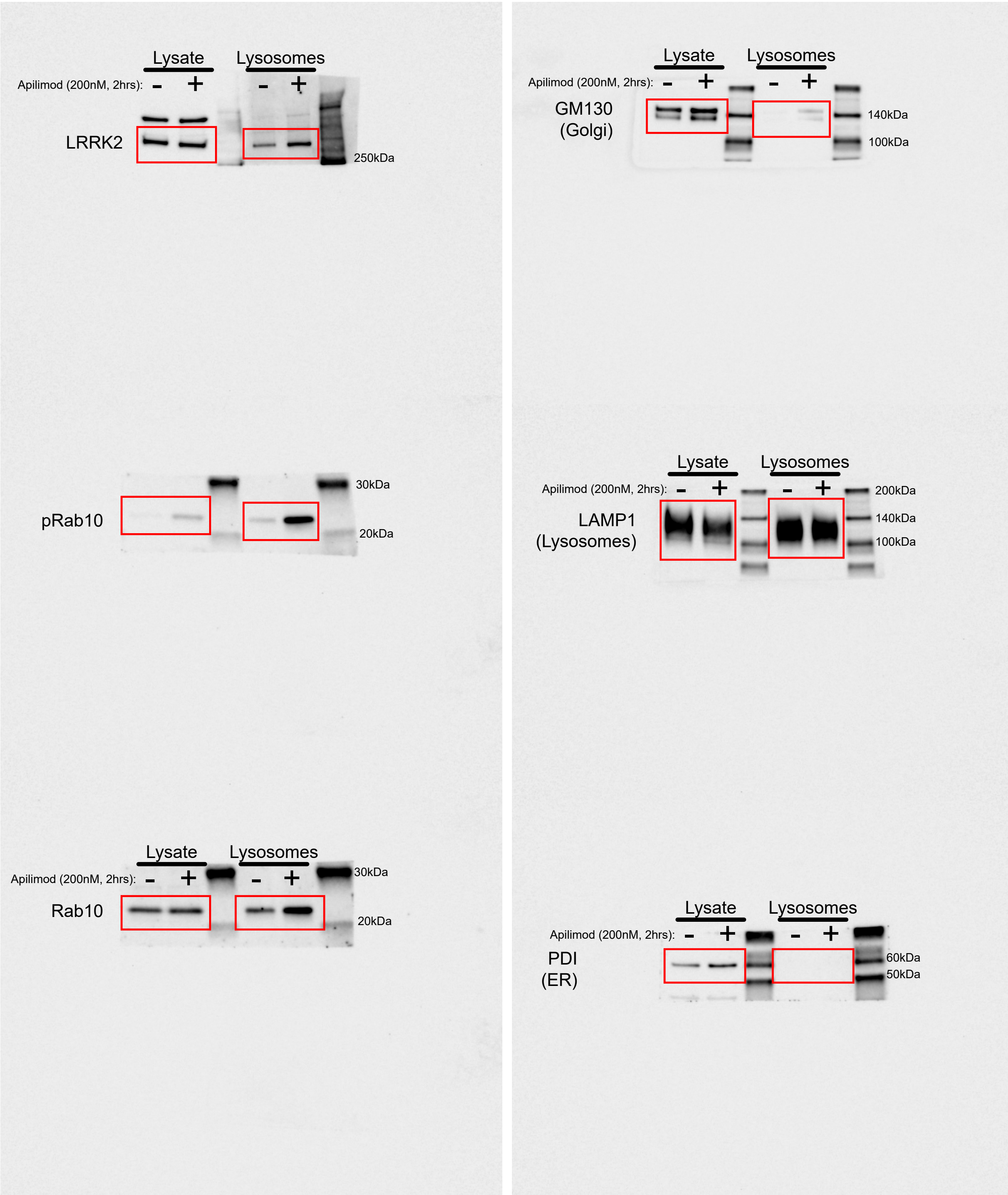
Lysate
Lysosomes
Apilimod (200nM, 2hrs):
- + - +
LRRK2
250kDa
pRab10
30kDa
20kDa
Rab10
30kDa
20kDa
GM130
(Golgi)
140kDa
100kDa
LAMP1
(Lysosomes)
200kDa
140kDa
100kDa
PDI
(ER)
60kDa
50kDa

1J

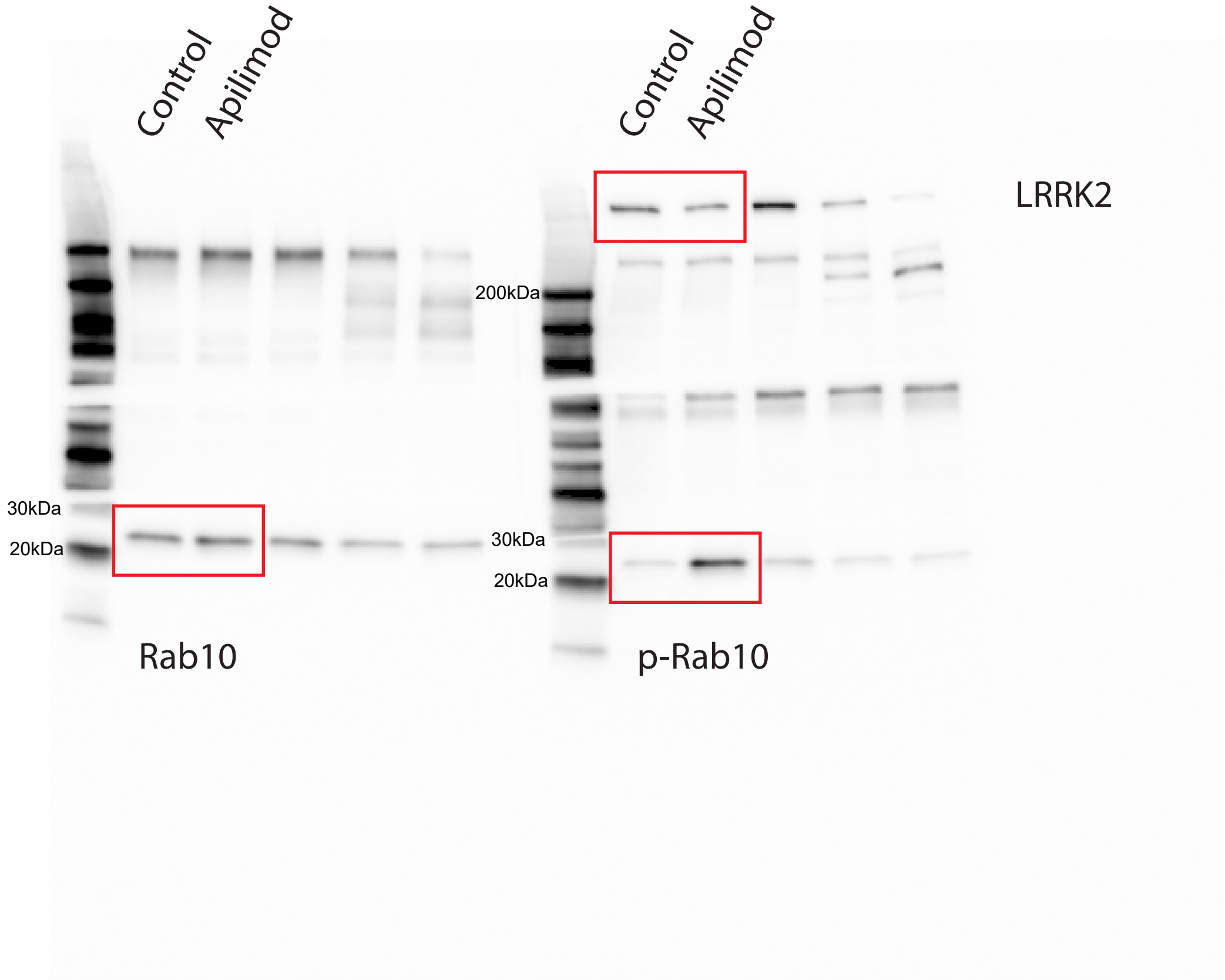
Control
Apilimod
Control
Apilimod
LRRK2
200kDa
30kDa
20kDa
30kDa
20kDa
Rab10
p-Rab10

1C

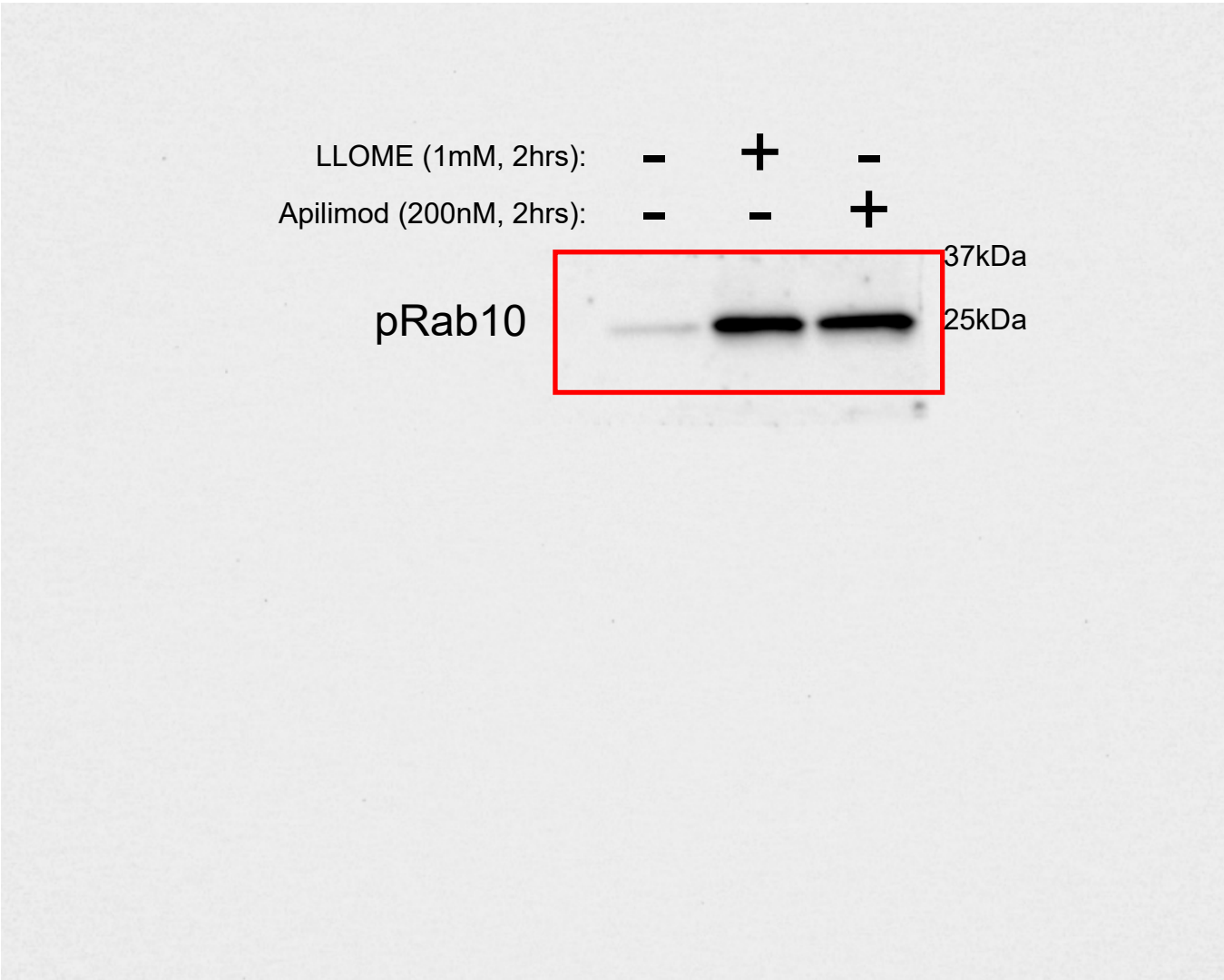
LLOME (1mM, 2hrs): - + -
Apilimod (200nM, 2hrs): - - +
37kDa
pRab10
25kDa

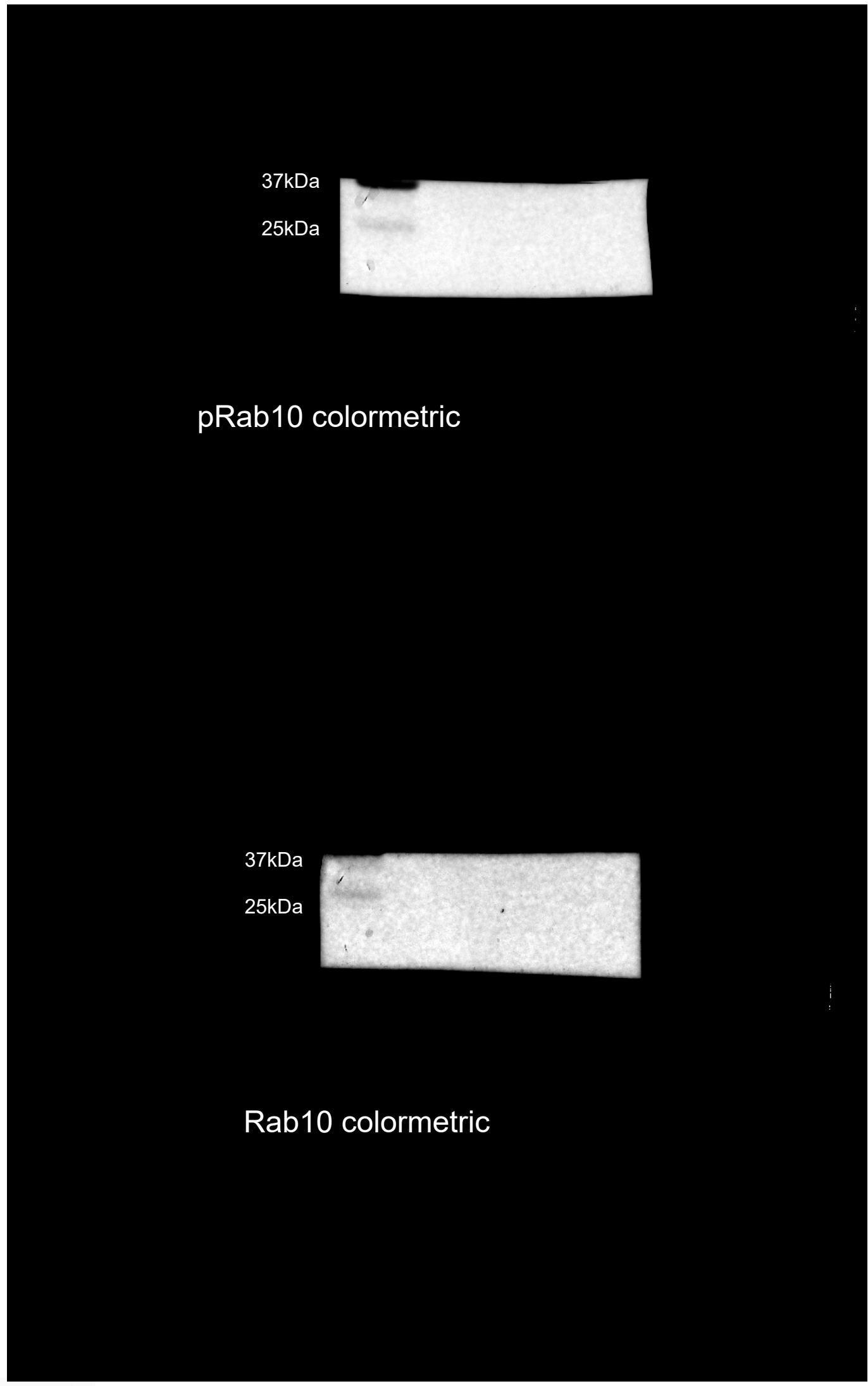
37kDa
25kDa
pRab10 colormetric
37kDa
25kDa
Rab10 colormetric

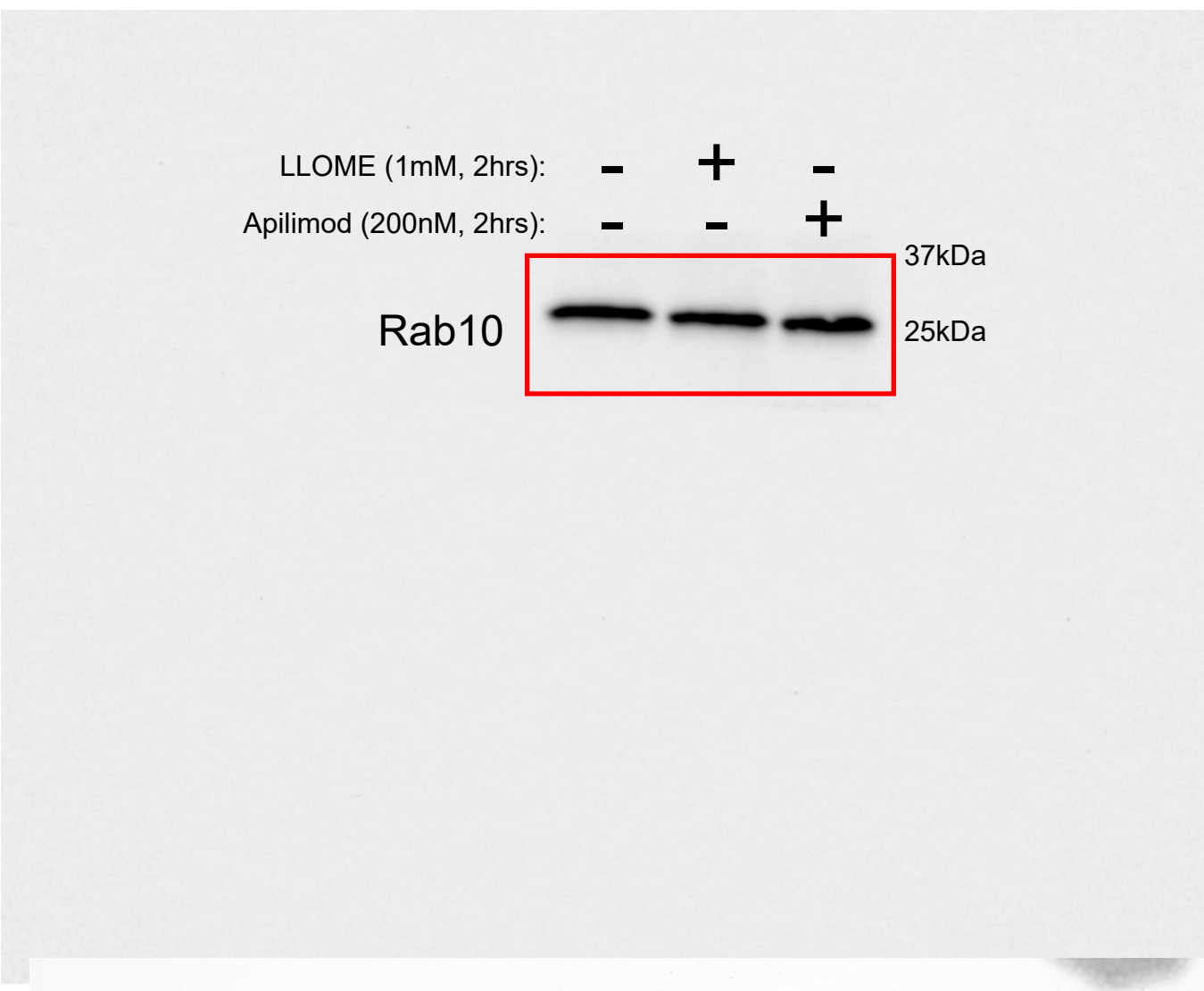
LLOME (1mM, 2hrs): - + -
Apilimod (200nM, 2hrs): - - +
37kDa
Rab10
25kDa

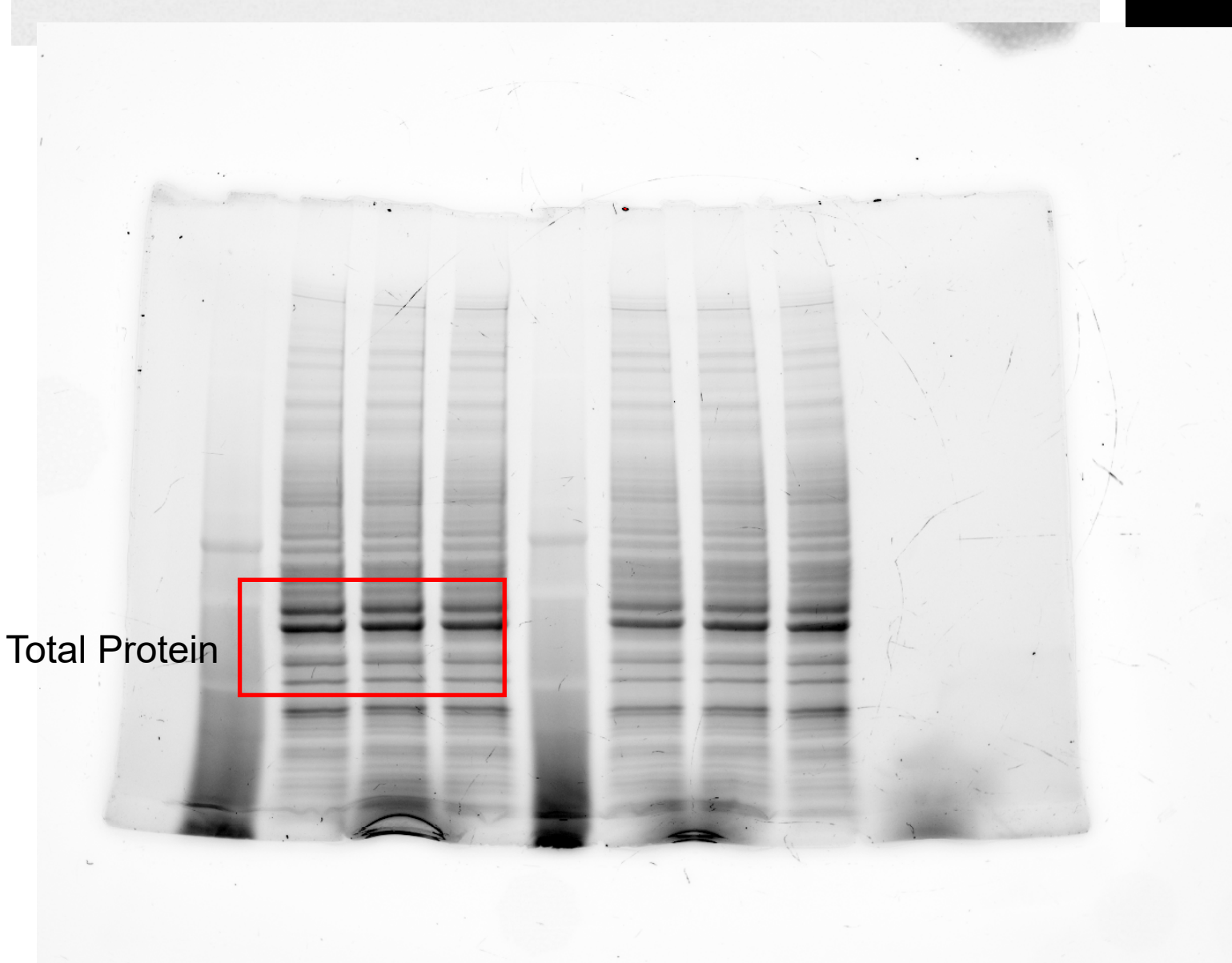
Total Protein

1E

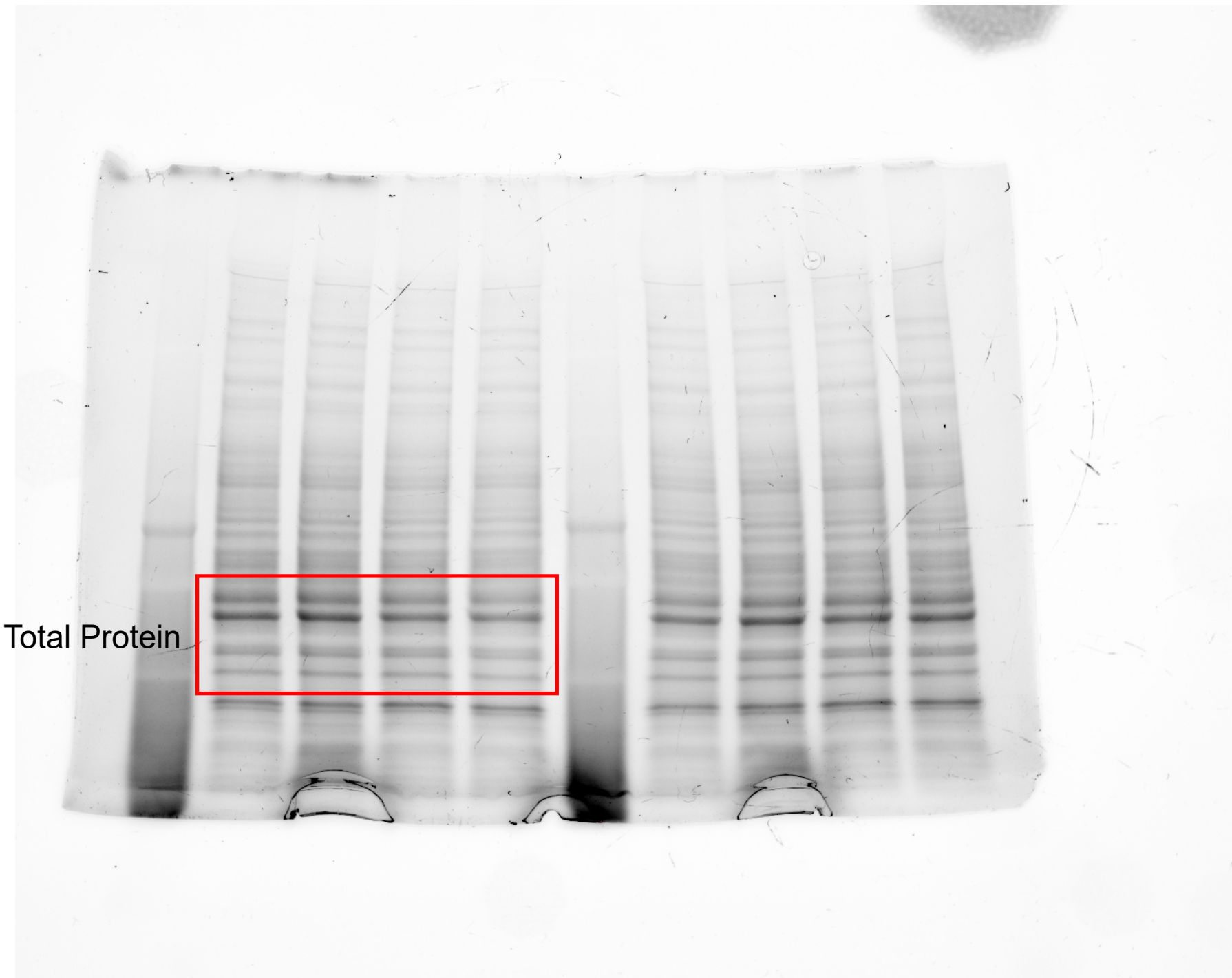

Supplement: Supplementary file 2 [file jcb_202511137_sourcedataf1.pdf]
