# Supplementary material for: ClC-7 links PIKfyve inhibition to Rab-dependent LRRK2 activity at lysosomes
Source: J Cell Biol. 2026 Oct 20;225(11):e202511137. doi: 10.1083/jcb.202511137 (PMC13682606; doi:10.1083/jcb.202511137)
Supplement: Supplementary file 3 [file jcb_202511137_sourcedataf2.pdf]

2C

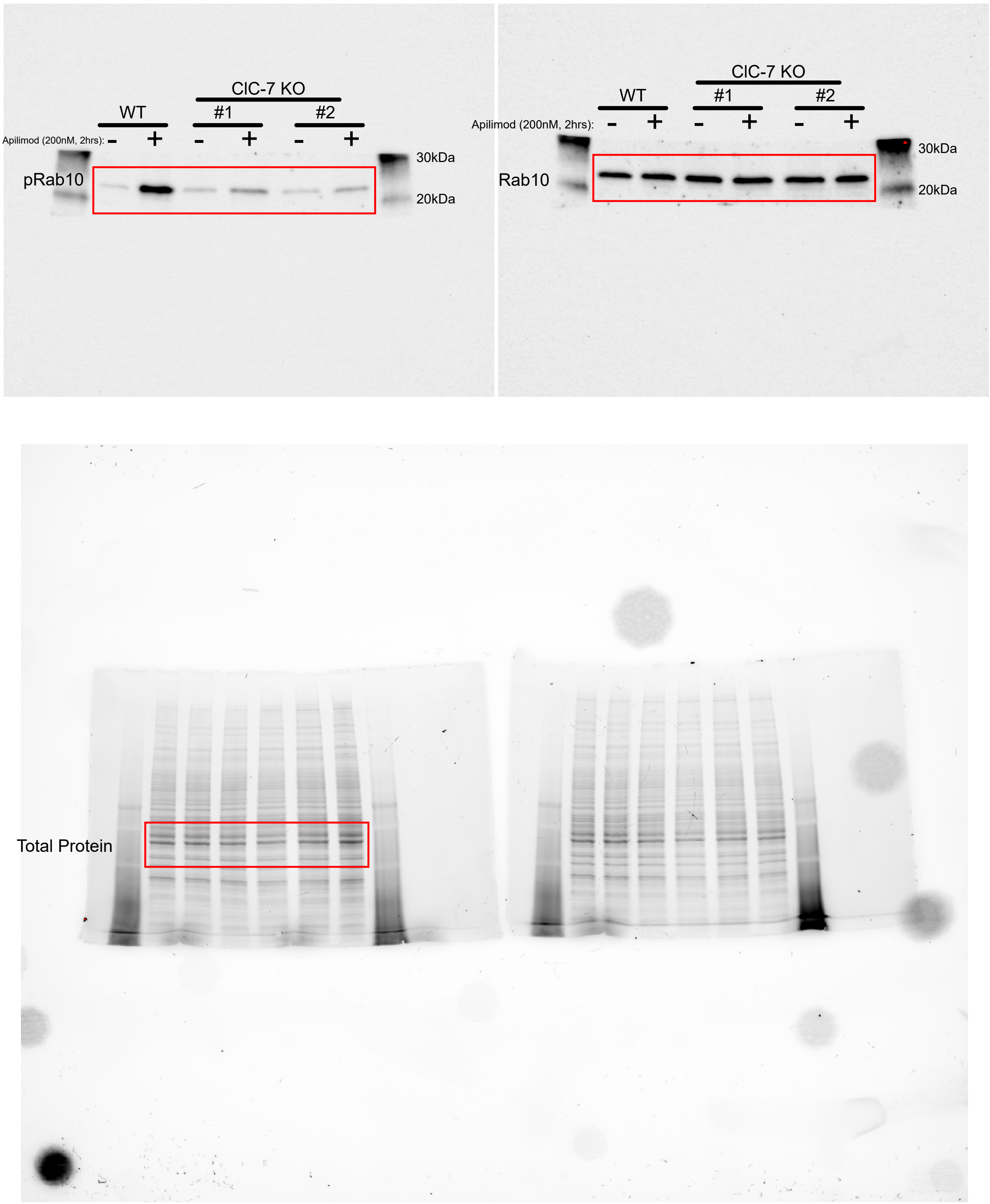
ClC-7 KO
WT
#1
#2
Apilimod (200nM, 2hrs): - + - + - +
pRab10
30kDa
20kDa
ClC-7 KO
WT
#1
#2
Apilimod (200nM, 2hrs): - + - + - +
Rab10
30kDa
20kDa
Total Protein

2E

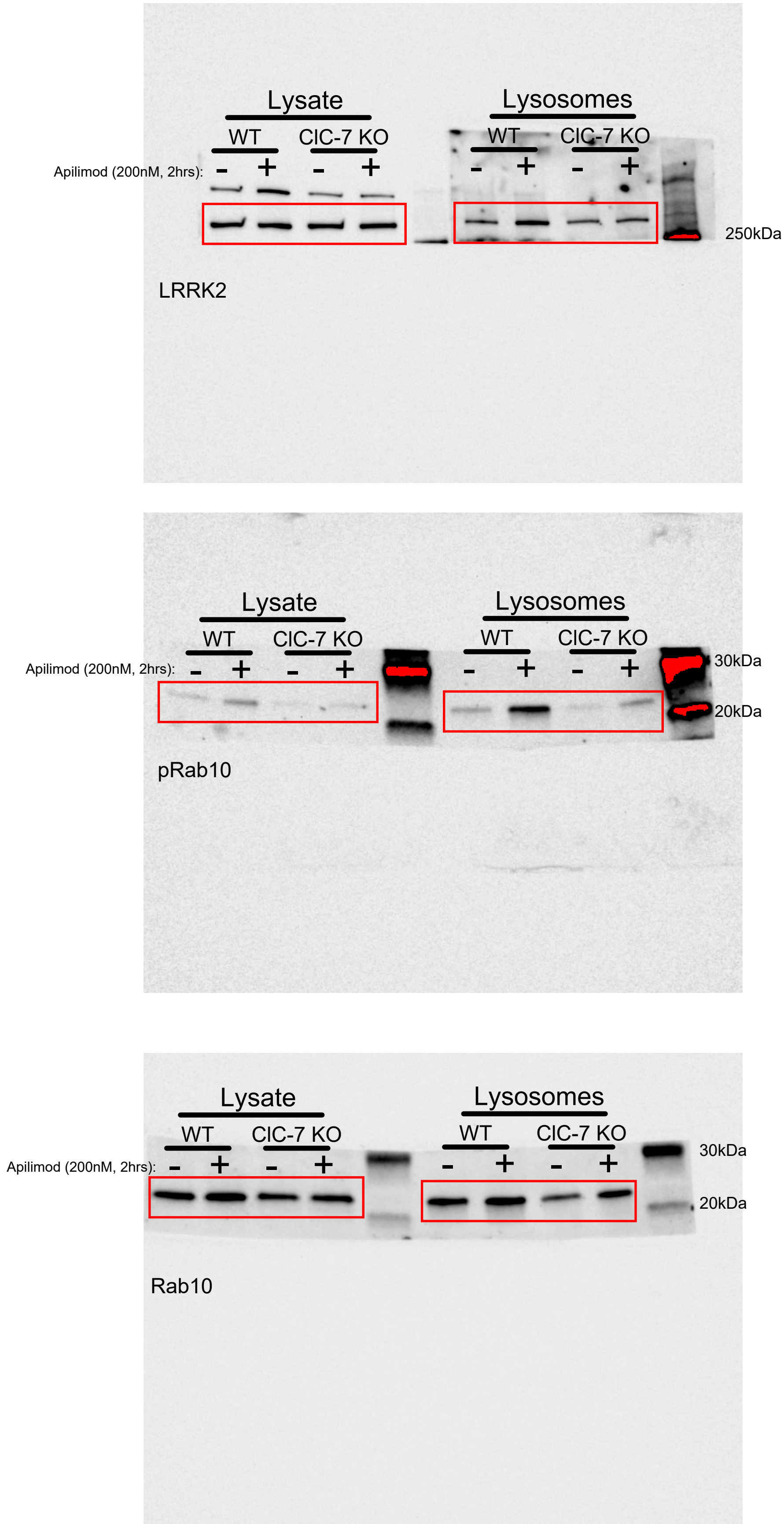
Lysate
Lysosomes
WT
ClC-7 KO
WT
ClC-7 KO
Apilimod (200nM, 2hrs): - + - + - + - +
250kDa
LRRK2
Lysate
Lysosomes
WT
ClC-7 KO
WT
ClC-7 KO
Apilimod (200nM, 2hrs): - + - + - + - +
30kDa
20kDa
pRab10
Lysate
Lysosomes
WT
ClC-7 KO
WT
ClC-7 KO
Apilimod (200nM, 2hrs): - + - + - + - +
30kDa
20kDa
Rab10

2E

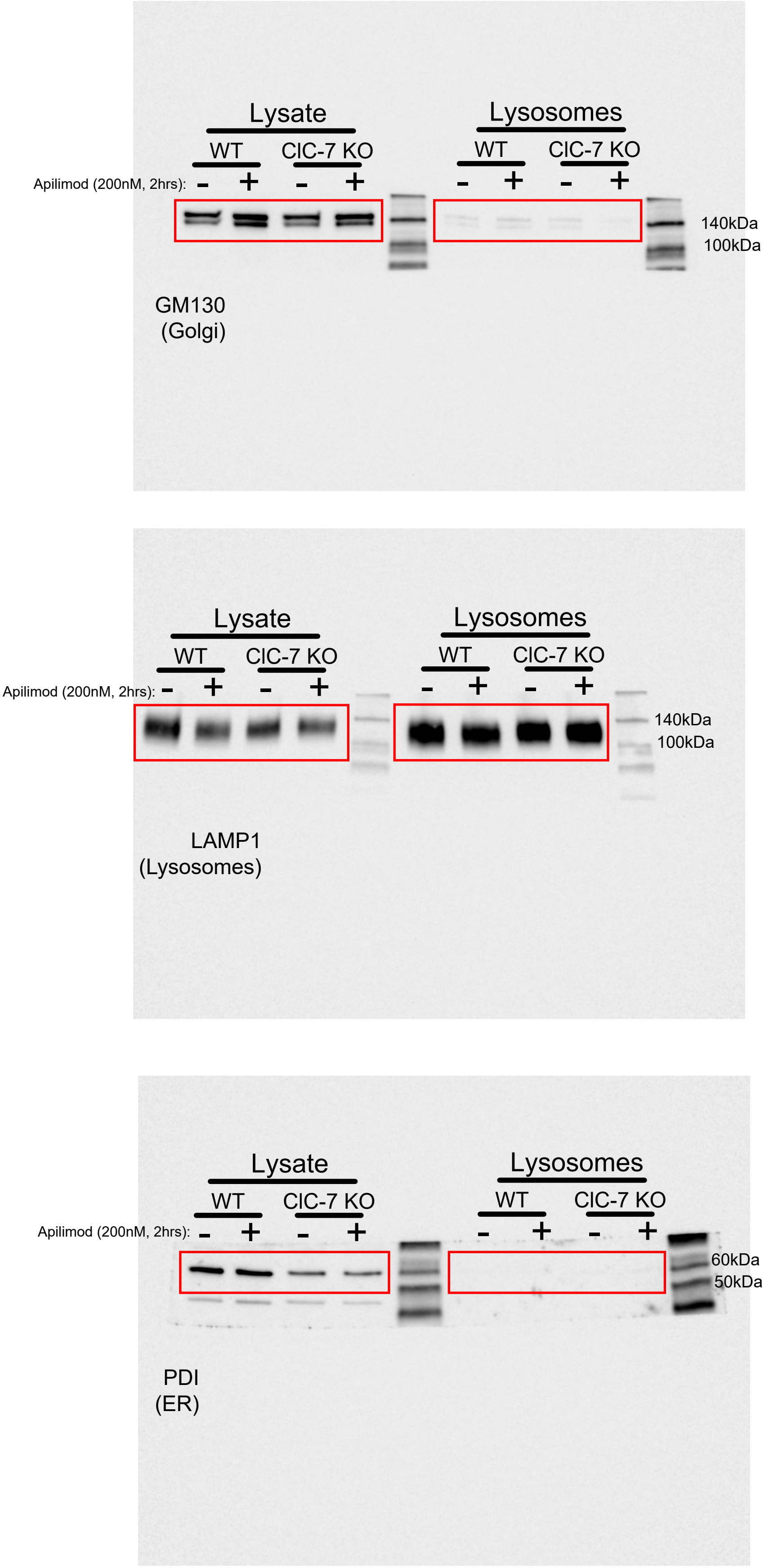
Lysate
WT
ClC-7 KO
Lysosomes
WT
ClC-7 KO
Apilimod (200nM, 2hrs): - + - + - + - +
140kDa
100kDa
GM130
(Golgi)
Lysate
WT
ClC-7 KO
Lysosomes
WT
ClC-7 KO
Apilimod (200nM, 2hrs): - + - + - + - +
140kDa
100kDa
LAMP1
(Lysosomes)
Lysate
WT
ClC-7 KO
Lysosomes
WT
ClC-7 KO
Apilimod (200nM, 2hrs): - + - + - + - +
60kDa
50kDa
PDI
(ER)
