# Supplementary material for: ClC-7 links PIKfyve inhibition to Rab-dependent LRRK2 activity at lysosomes
Source: J Cell Biol. 2026 Oct 20;225(11):e202511137. doi: 10.1083/jcb.202511137 (PMC13682606; doi:10.1083/jcb.202511137)
Supplement: Supplementary file 4 [file jcb_202511137_sourcedataf3.pdf]

3B

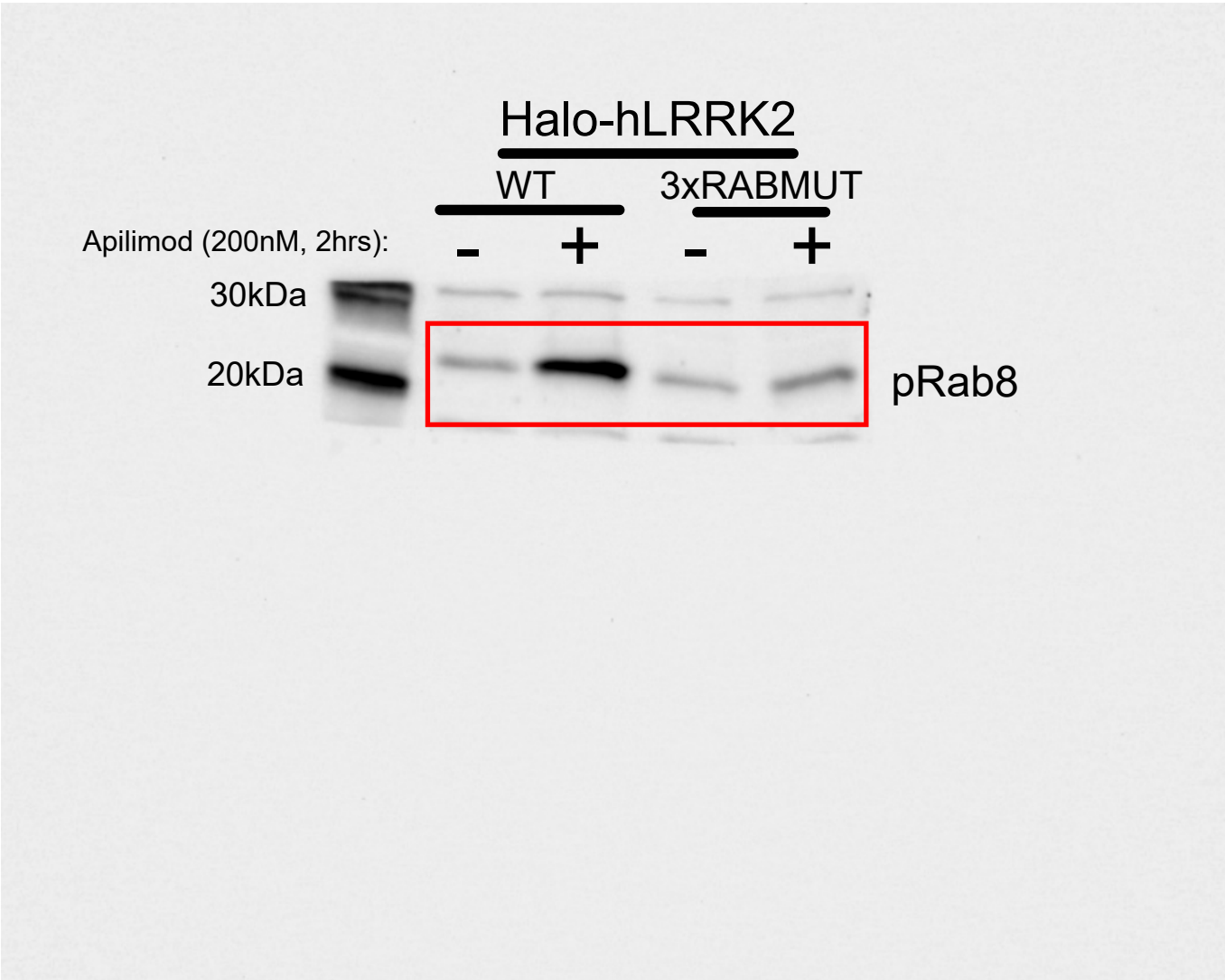

Halo-hLRRK2
WT
3xRABMUT
Apilimod (200nM, 2hrs):
- + - +
30kDa
20kDa
pRab8

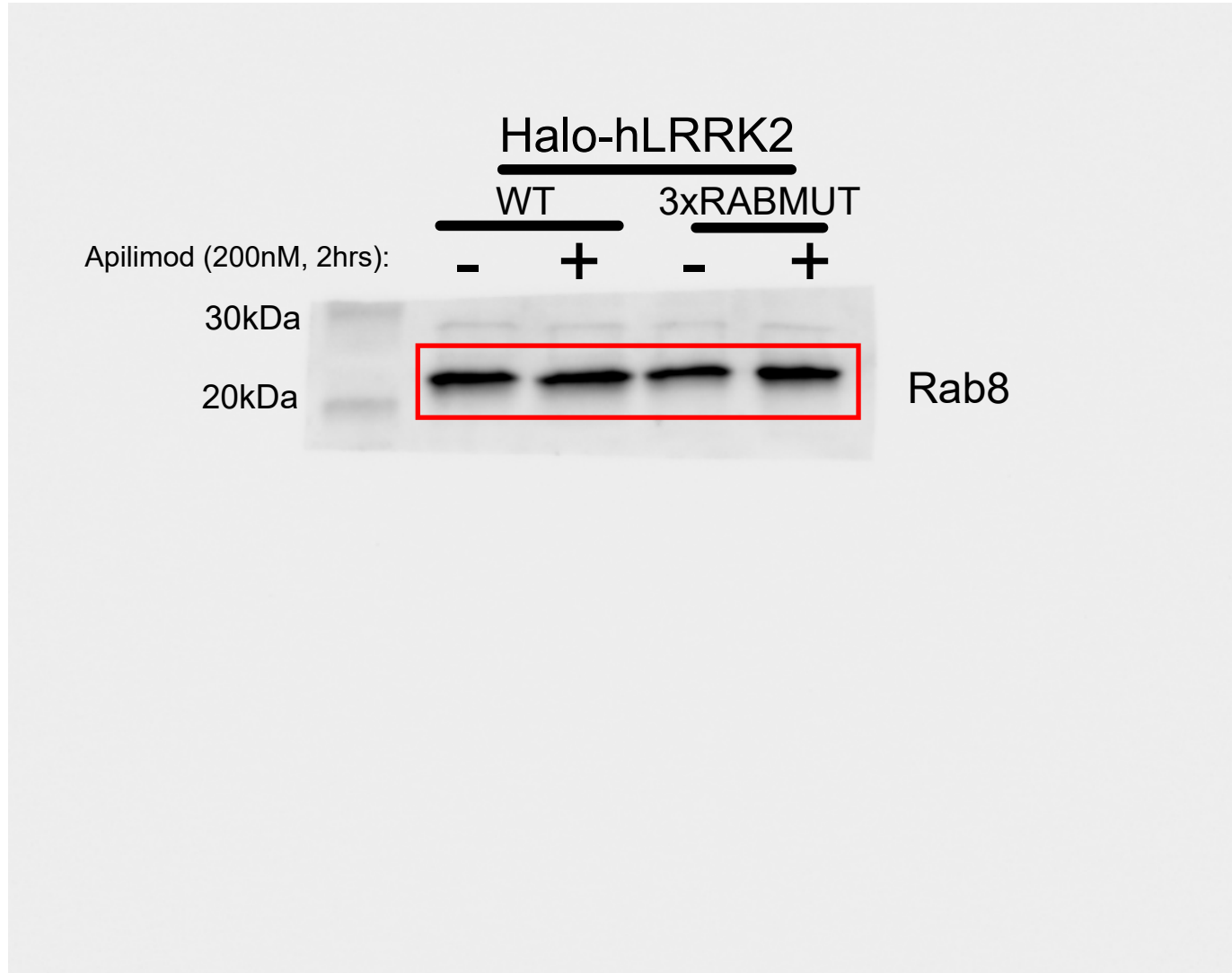

Halo-hLRRK2
WT
3xRABMUT
Apilimod (200nM, 2hrs):
- + - +
30kDa
20kDa
Rab8

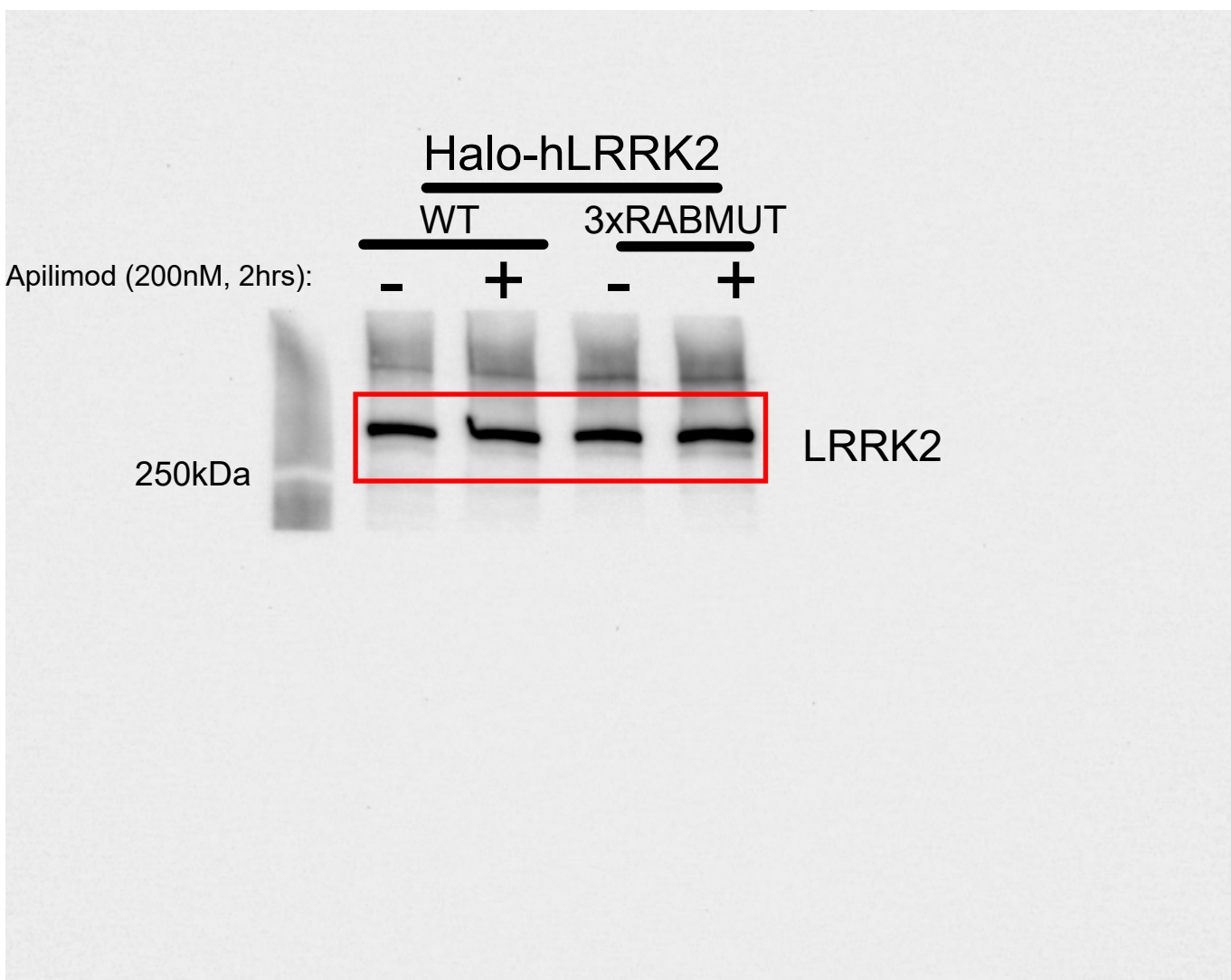

Halo-hLRRK2
WT
3xRABMUT
Apilimod (200nM, 2hrs):
- + - +
250kDa
LRRK2

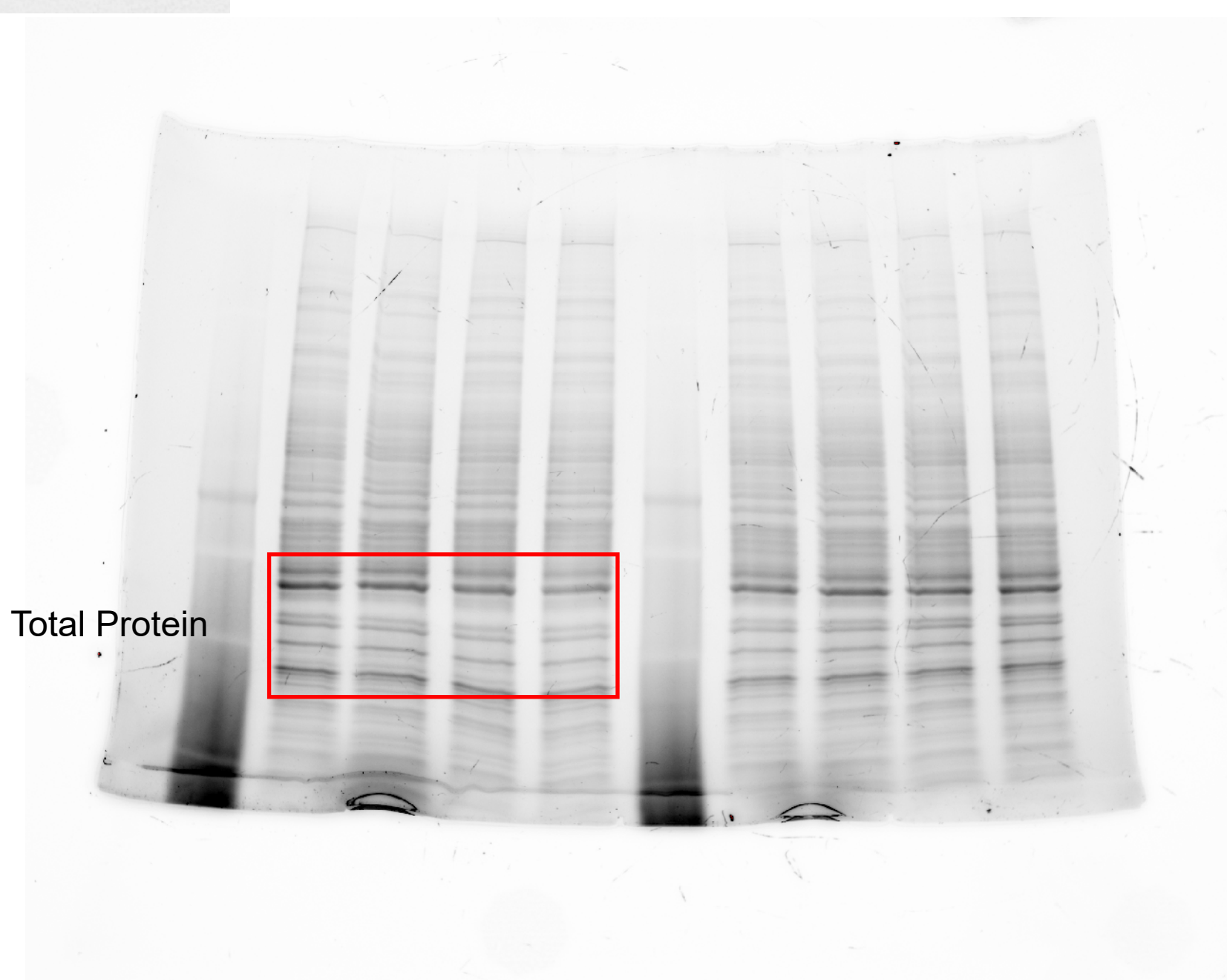

Total Protein

3D

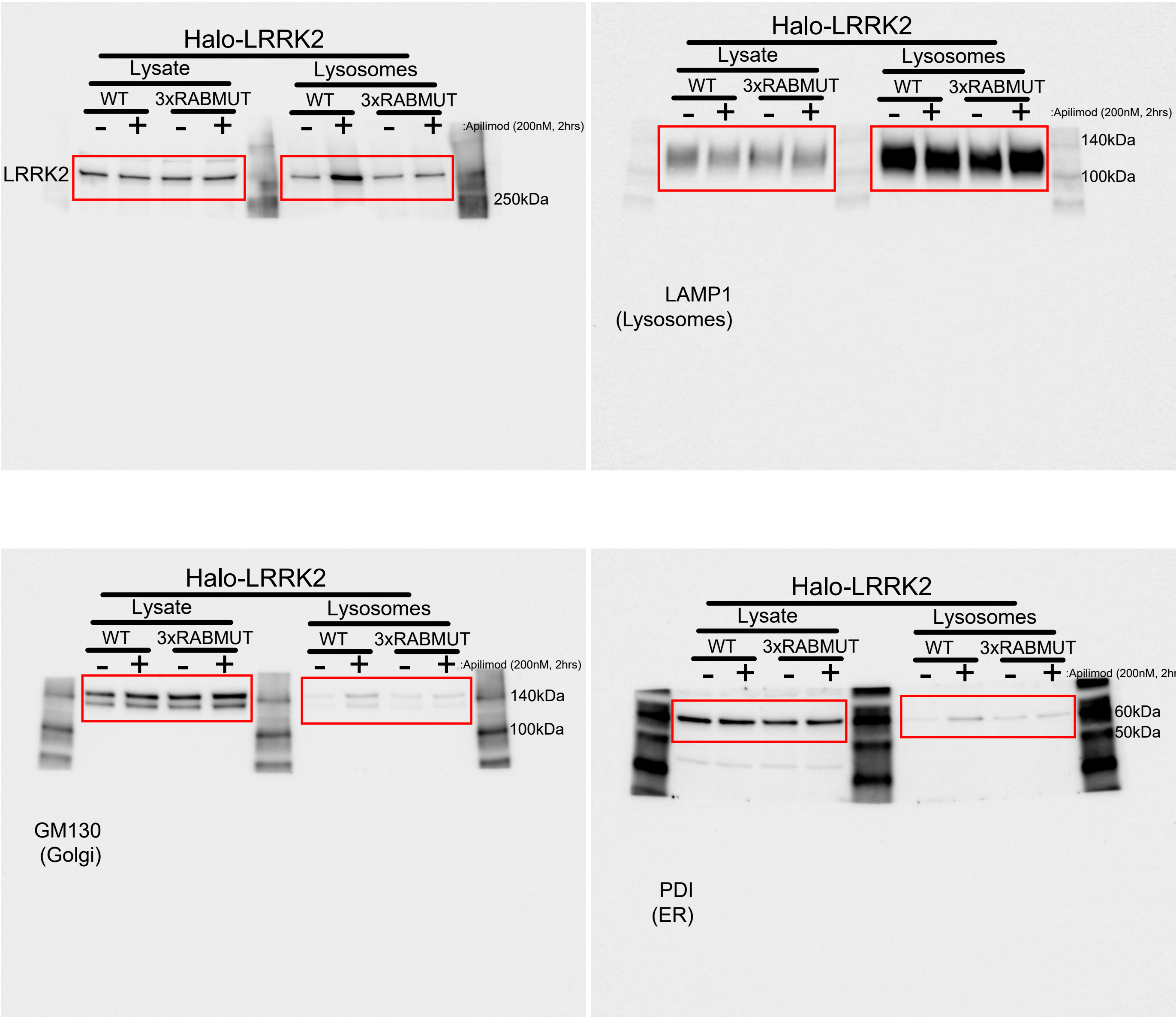
Halo-LRRK2
Lysate
Lysosomes
WT
3xRABMUT
WT
3xRABMUT
- + - +
- + - +
:Apilimod (200nM, 2hrs)
LRRK2
250kDa
Halo-LRRK2
Lysate
Lysosomes
WT
3xRABMUT
WT
3xRABMUT
- + - +
- + - +
:Apilimod (200nM, 2hrs)
140kDa
100kDa
LAMP1
(Lysosomes)
Halo-LRRK2
Lysate
Lysosomes
WT
3xRABMUT
WT
3xRABMUT
- + - +
- + - +
:Apilimod (200nM, 2hrs)
140kDa
100kDa
GM130
(Golgi)
Halo-LRRK2
Lysate
Lysosomes
WT
3xRABMUT
WT
3xRABMUT
- + - +
- + - +
60kDa
50kDa
PDI
(ER)

3F

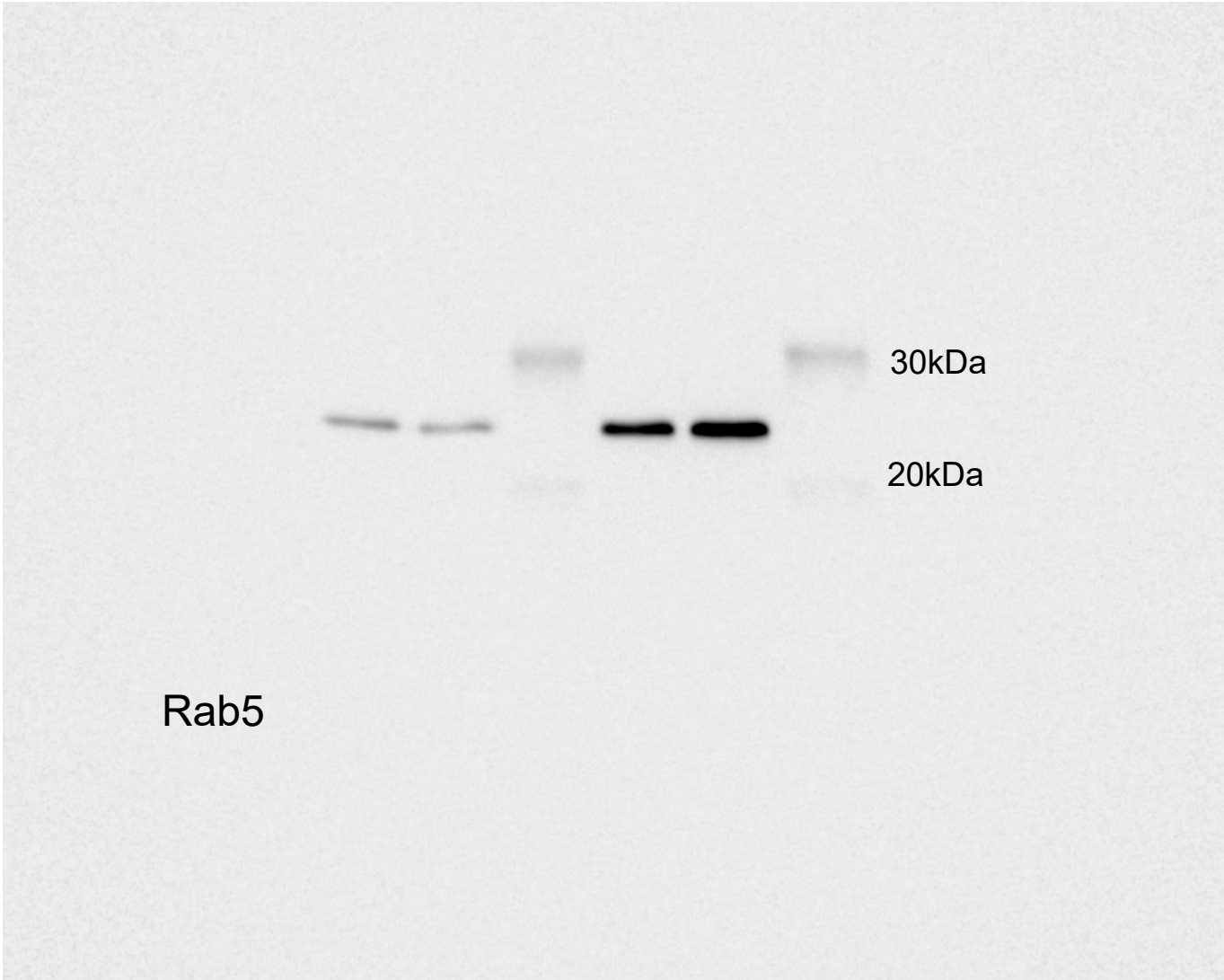
30kDa
20kDa
Rab5

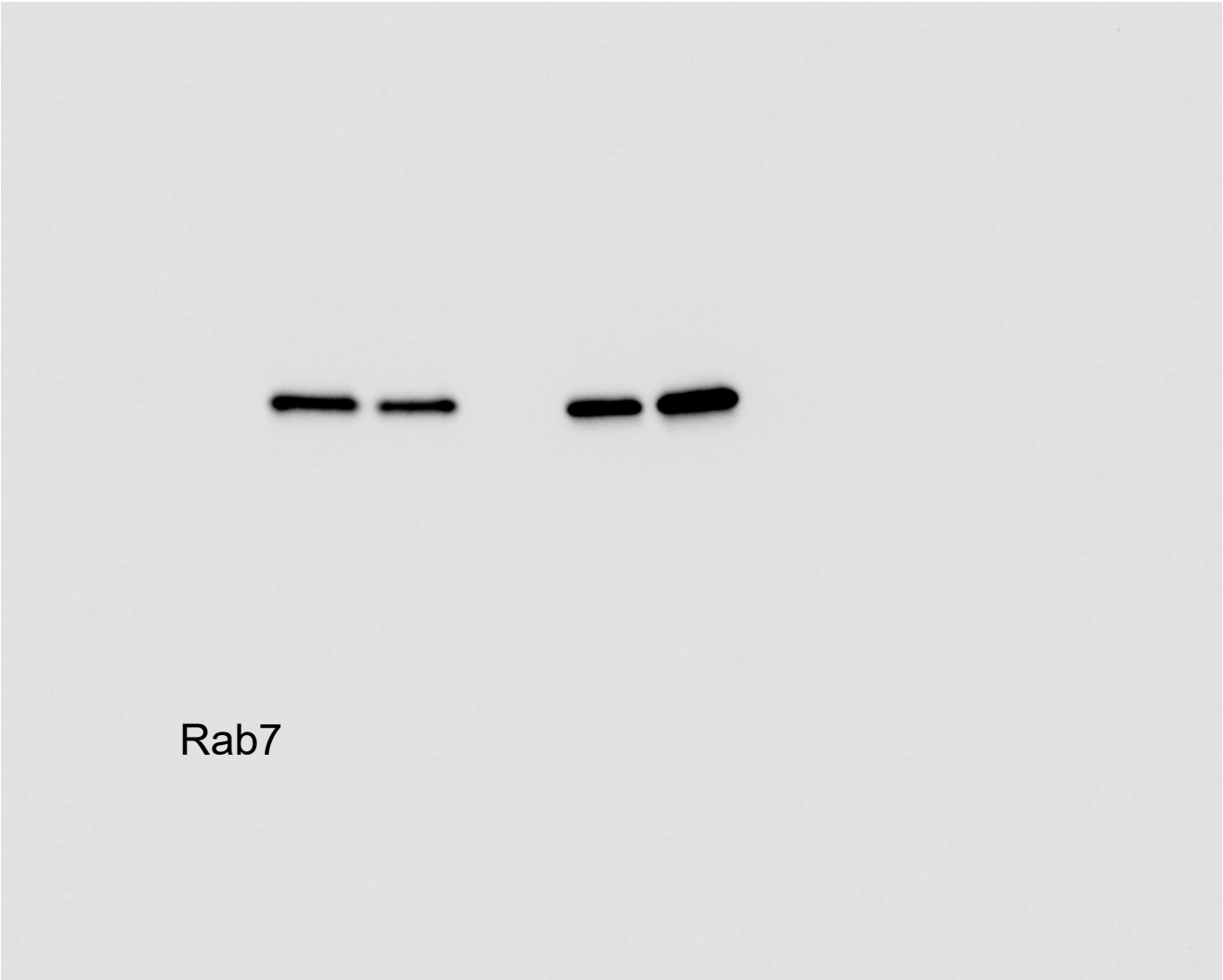
Rab7

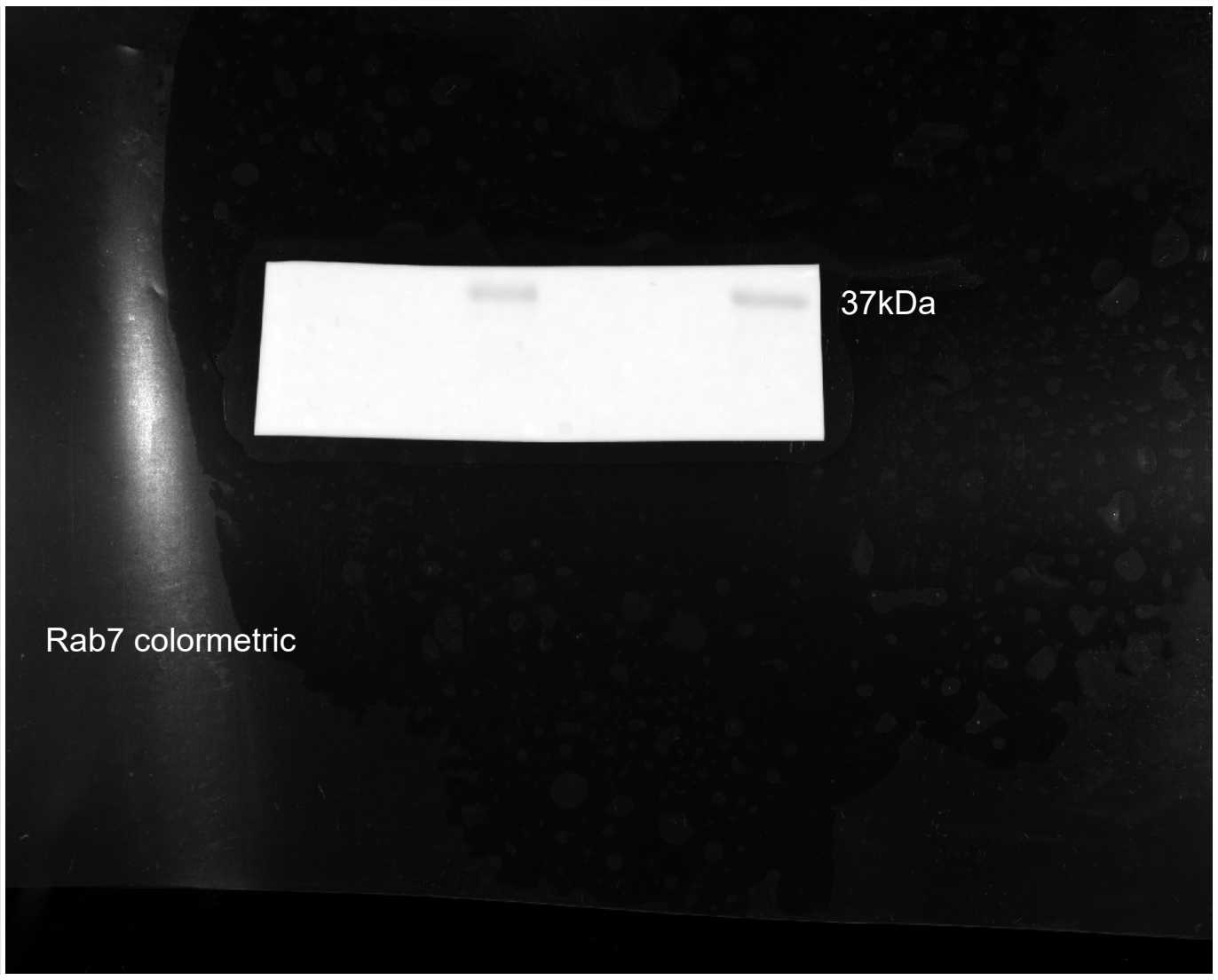
37kDa
Rab7 colormetric

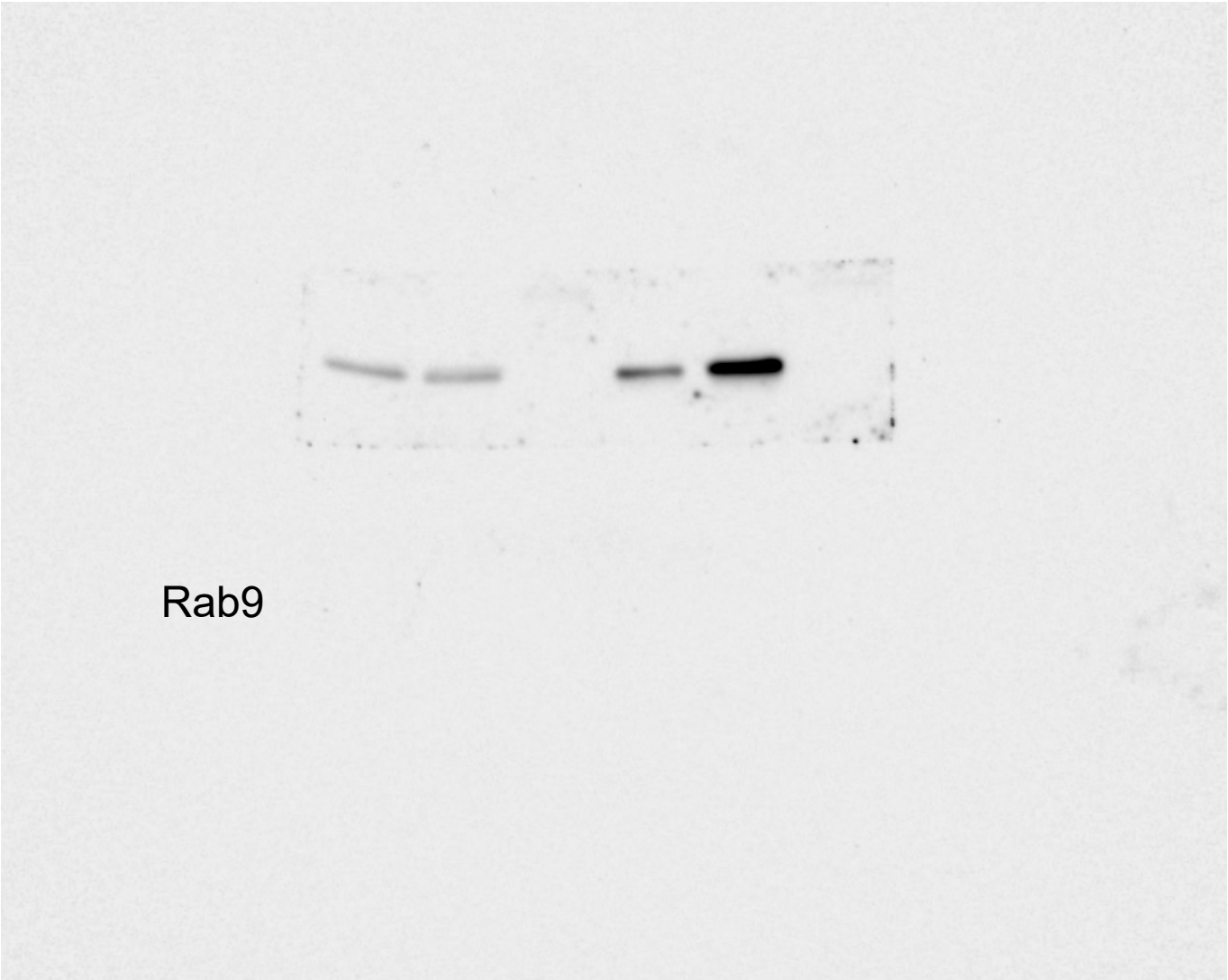
Rab9

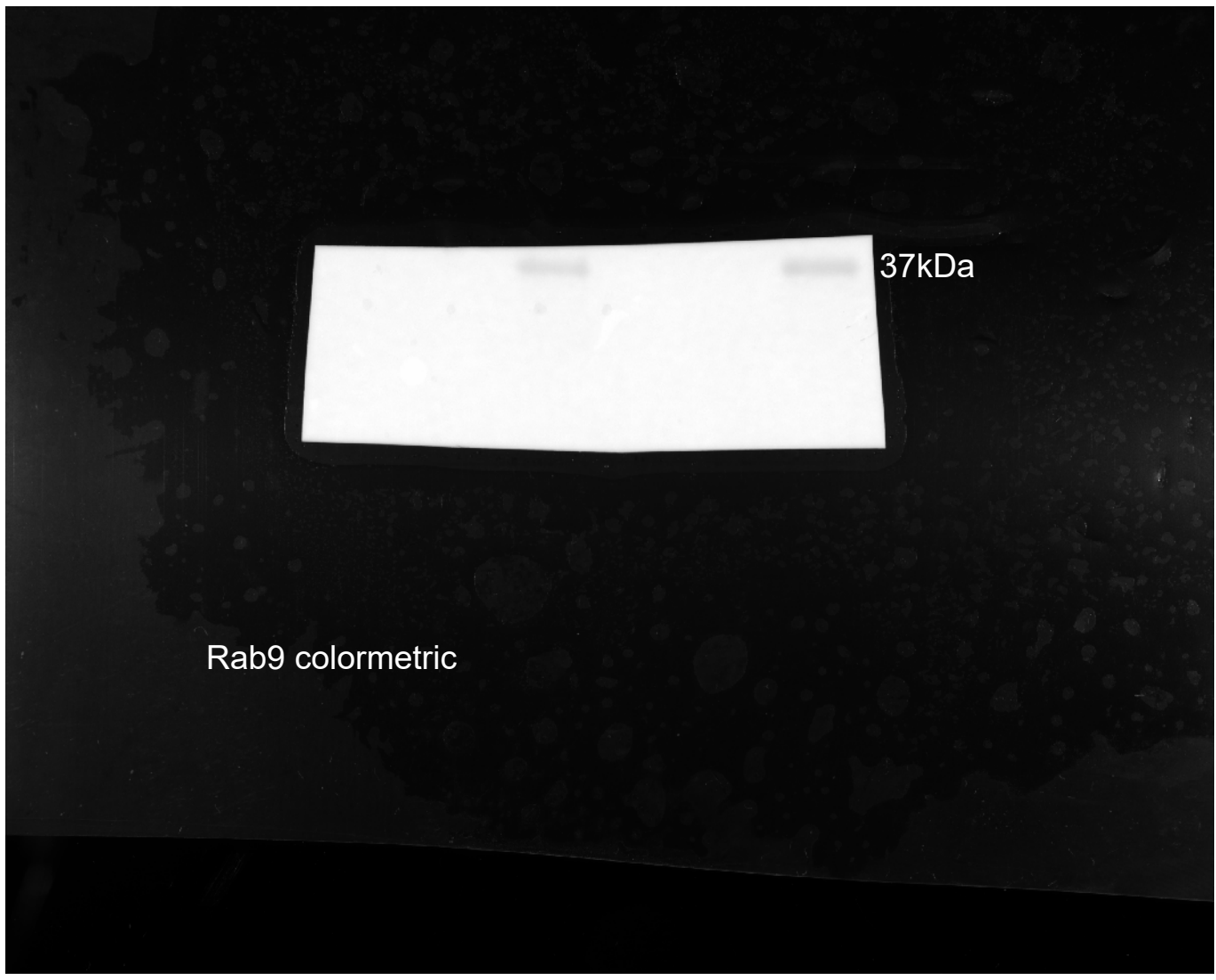
37kDa
Rab9 colormetric

3F

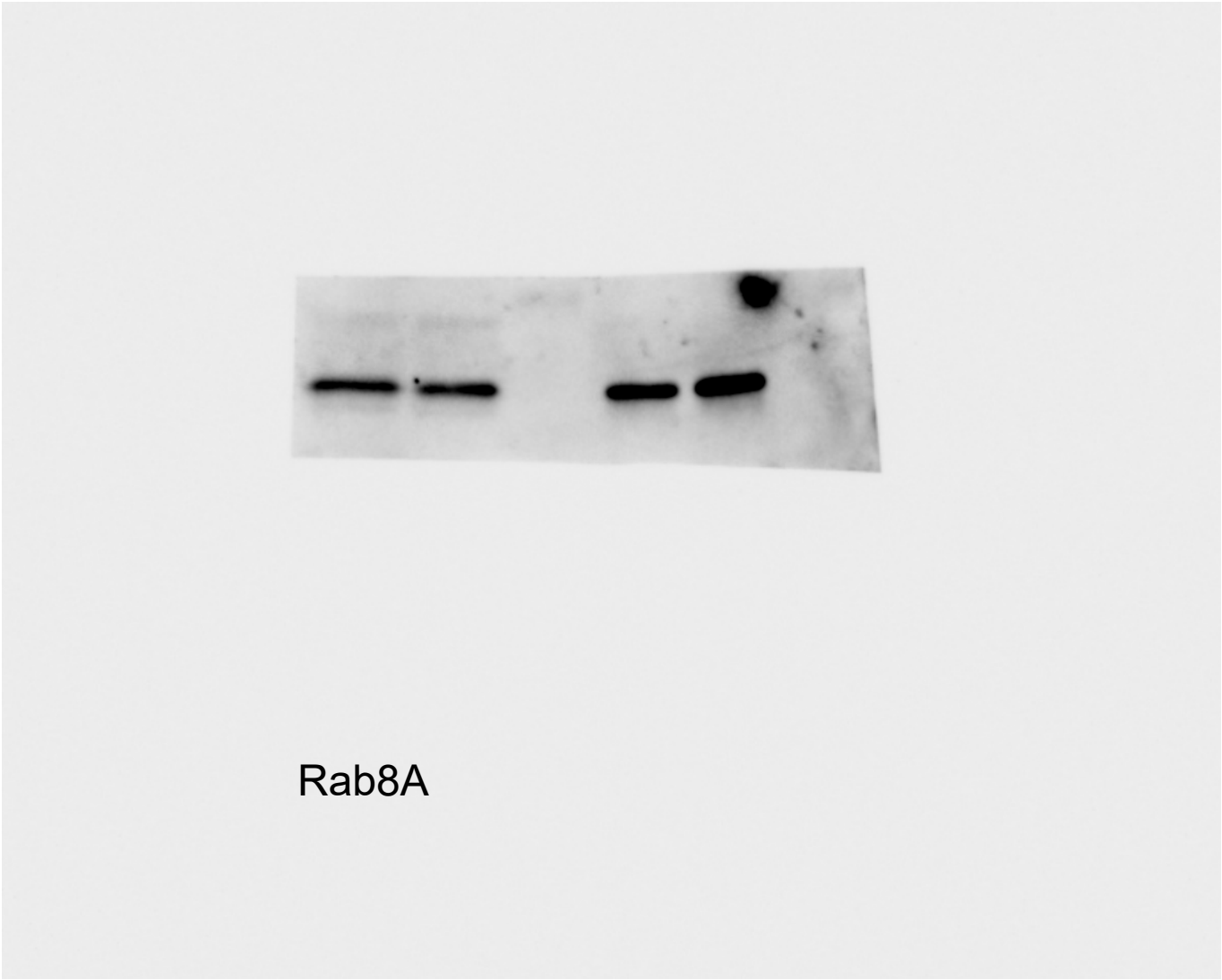
Rab8A

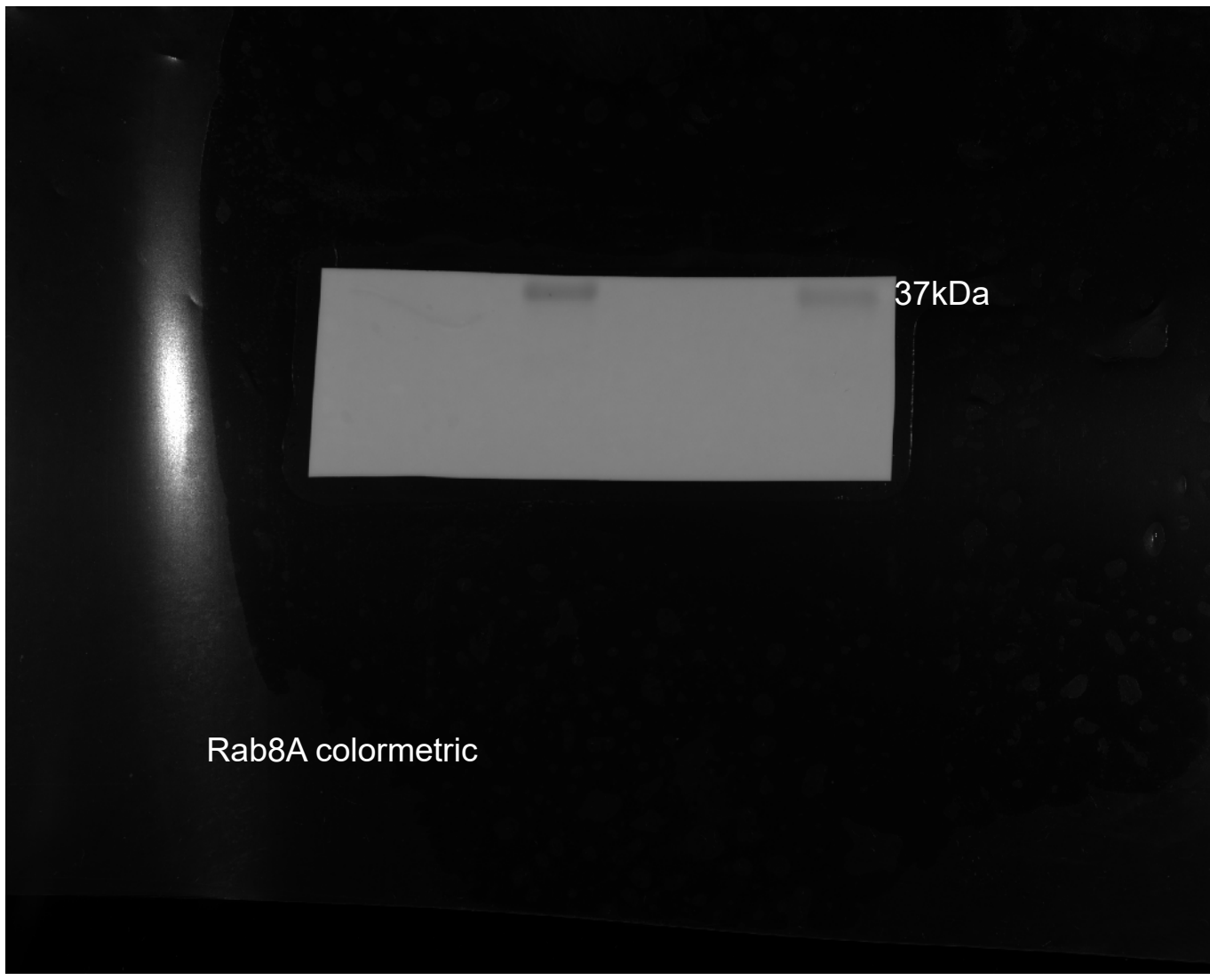
37kDa
Rab8A colormetric

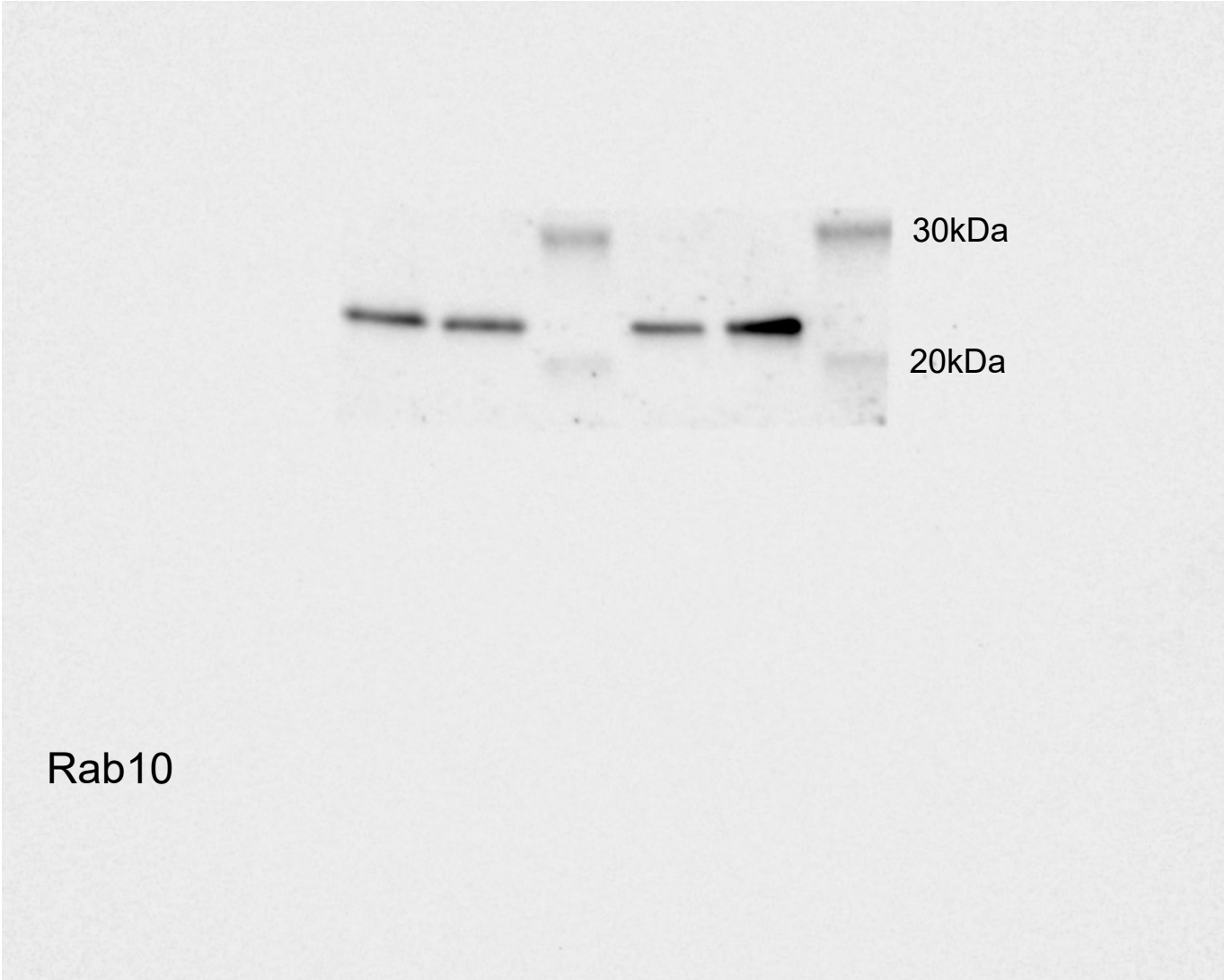
30kDa
20kDa
Rab10

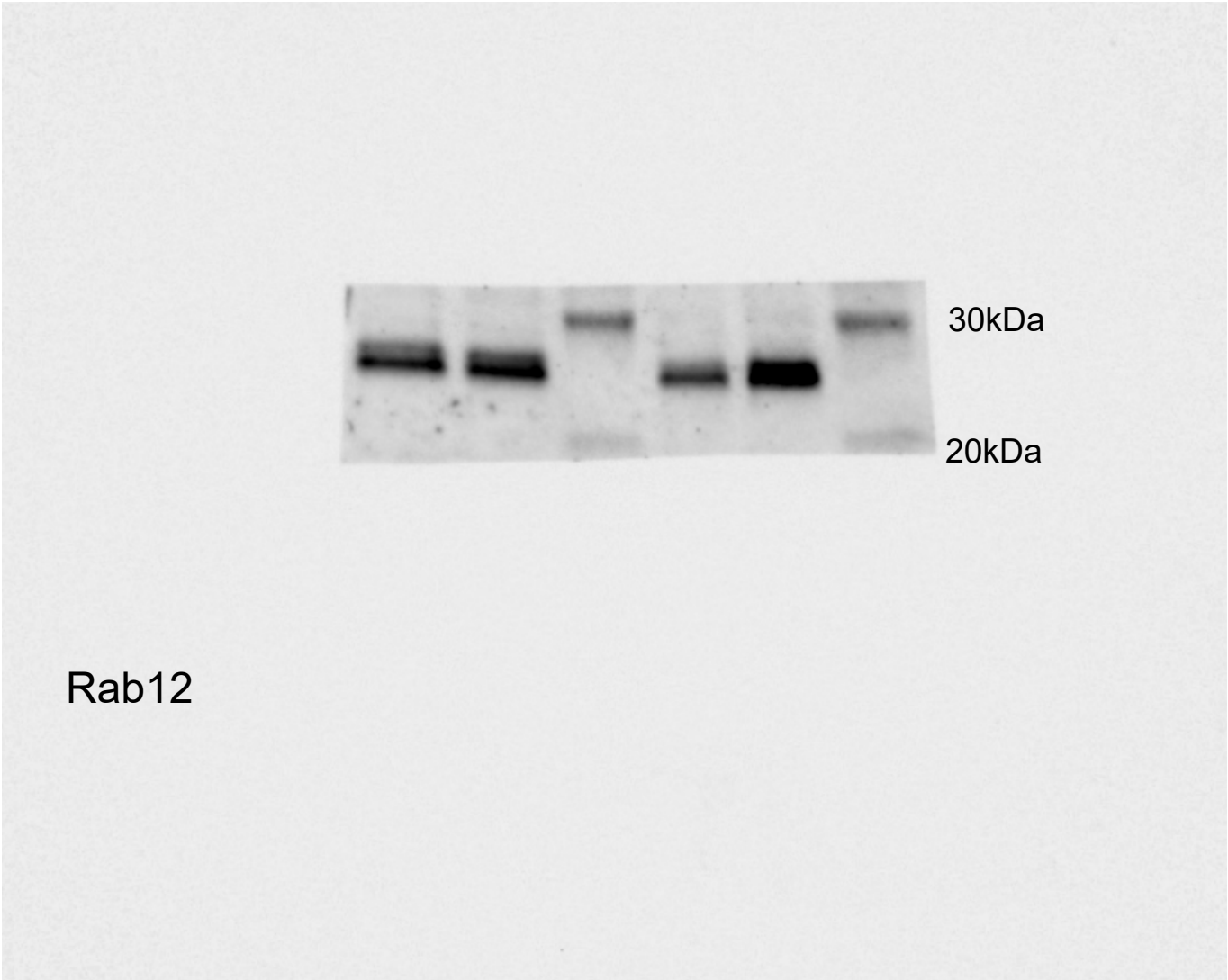
30kDa
20kDa
Rab12

3F

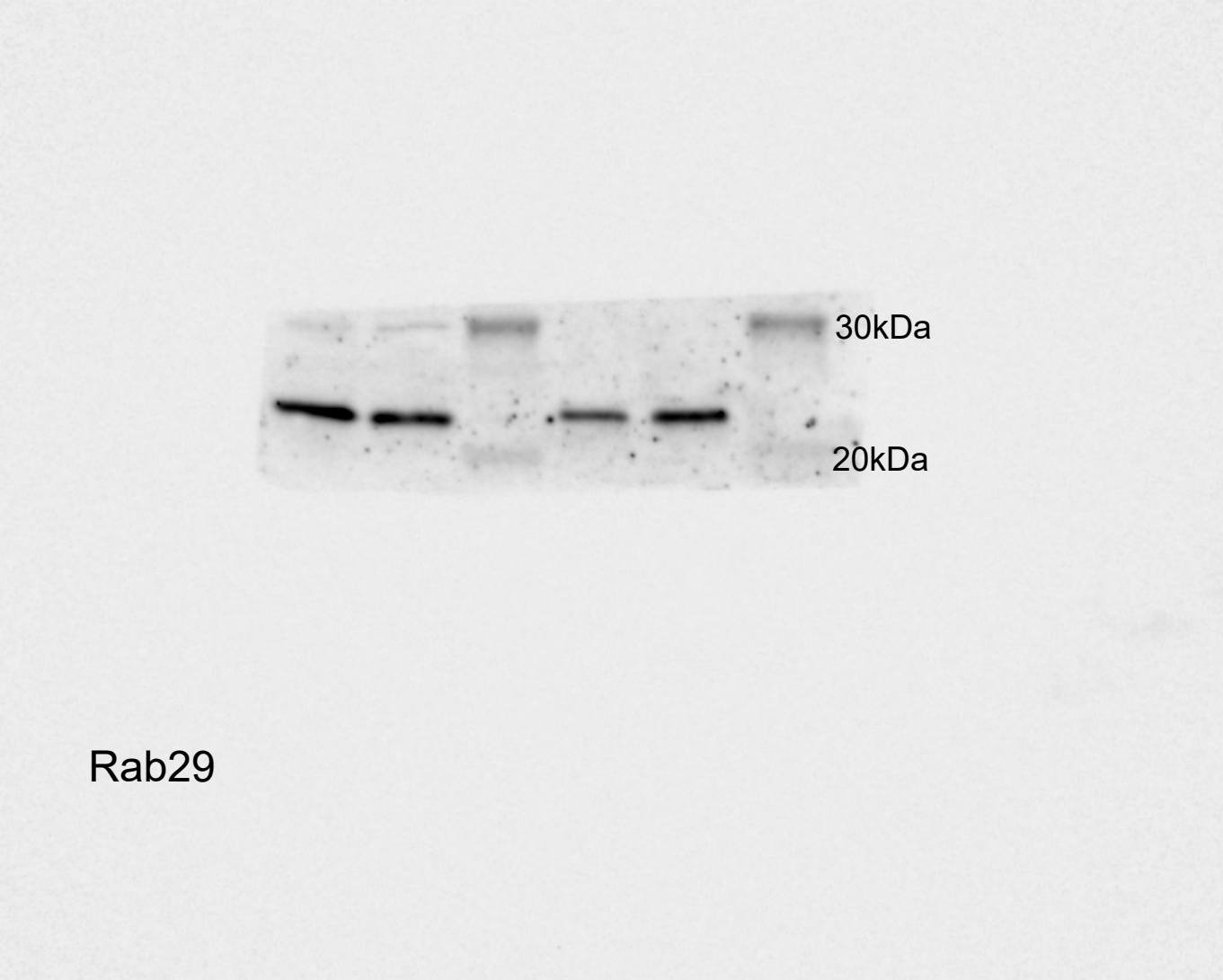

30kDa
20kDa
Rab29

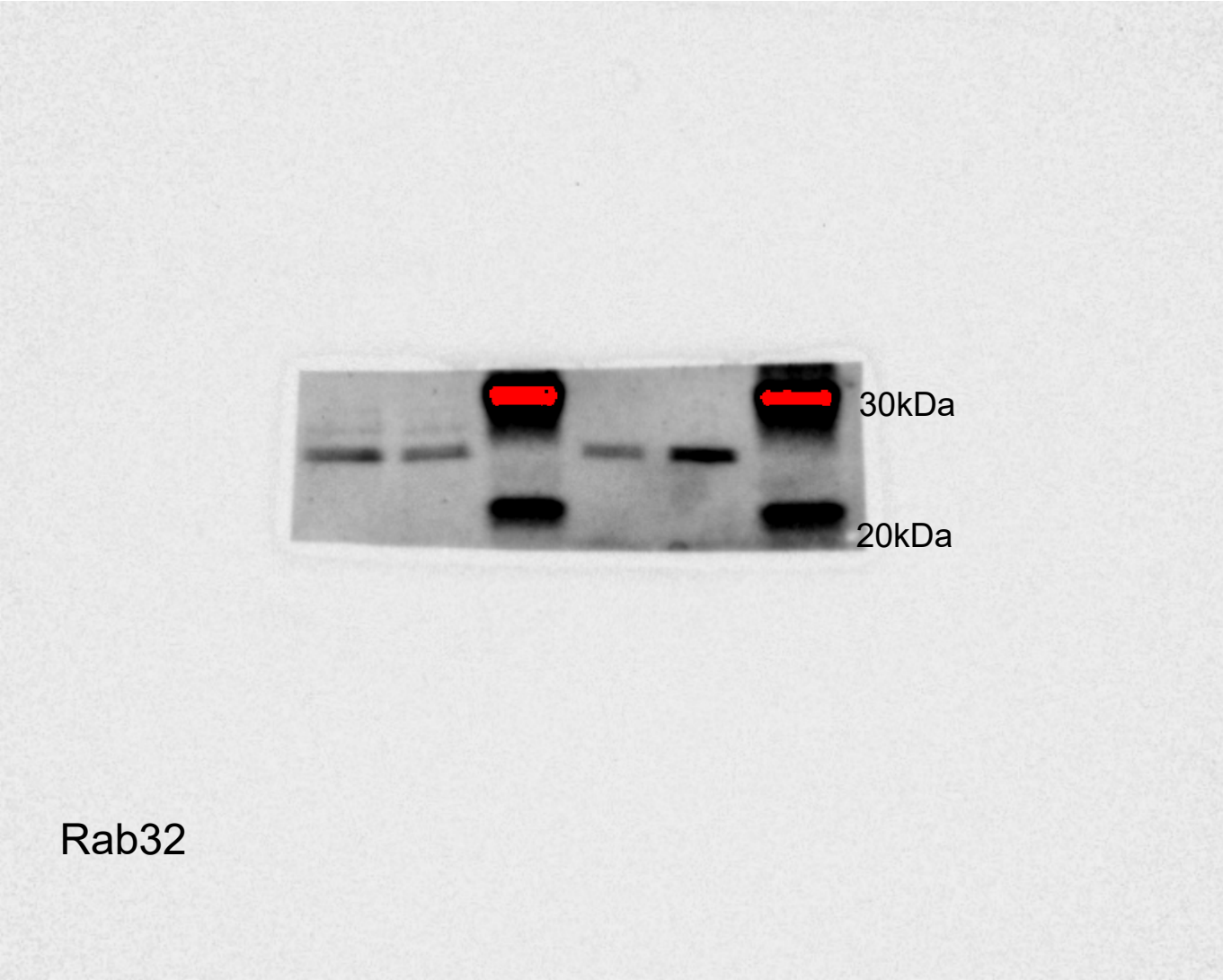

30kDa
20kDa
Rab32

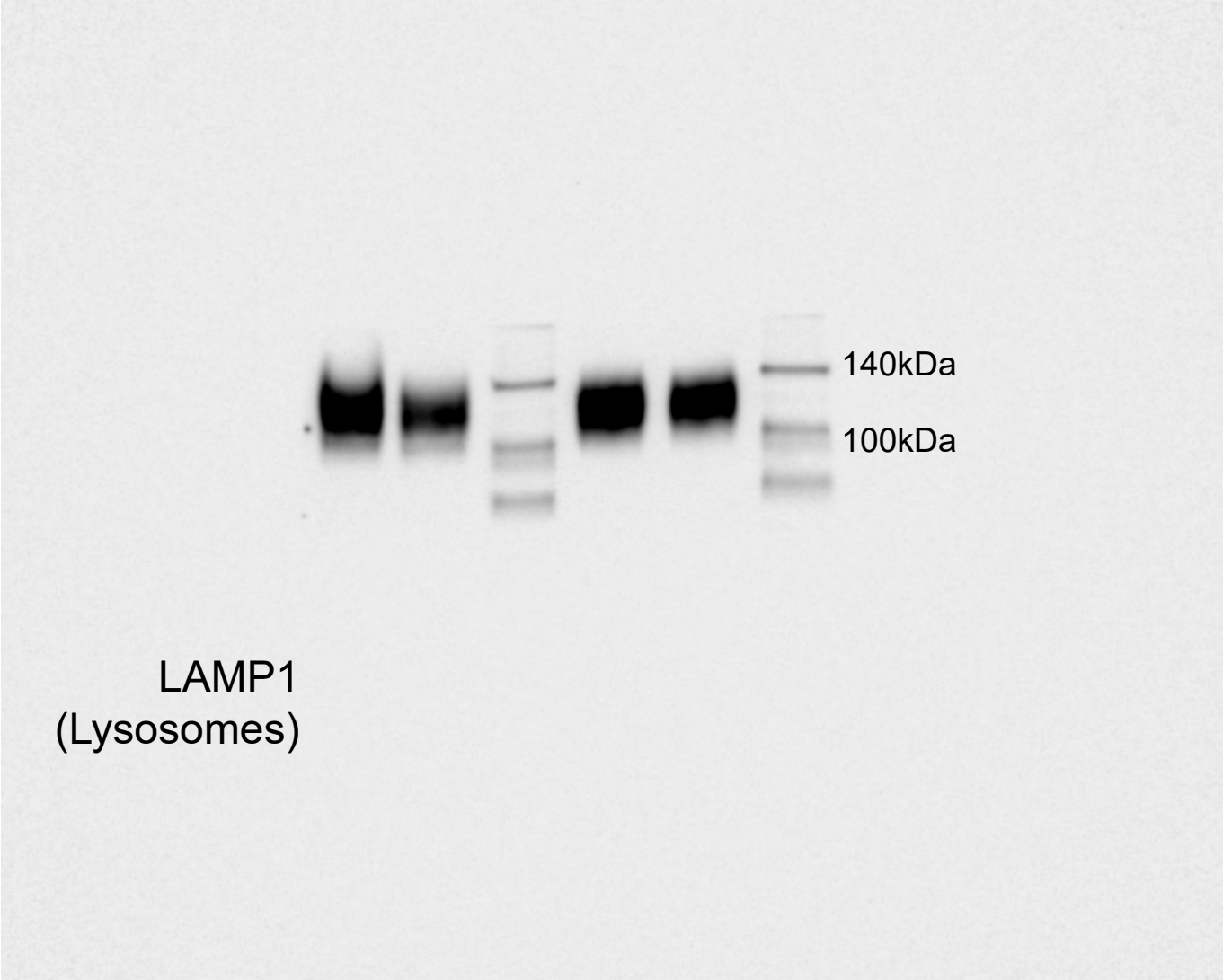

140kDa
100kDa
LAMP1
(Lysosomes)
