# Supplementary material for: ClC-7 links PIKfyve inhibition to Rab-dependent LRRK2 activity at lysosomes
Source: J Cell Biol. 2026 Oct 20;225(11):e202511137. doi: 10.1083/jcb.202511137 (PMC13682606; doi:10.1083/jcb.202511137)
Supplement: Supplementary file 5 [file jcb_202511137_sourcedataf4.pdf]

4A

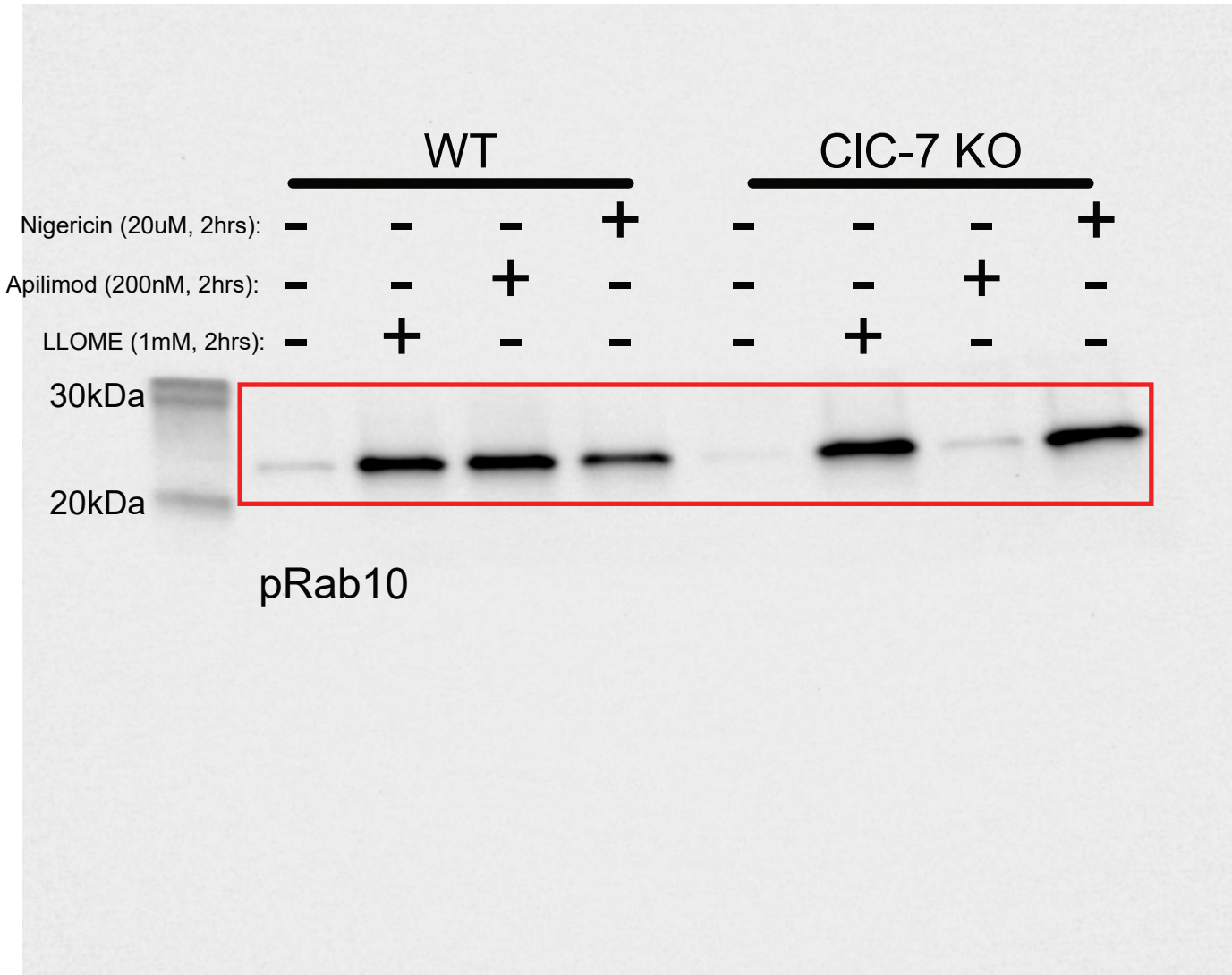
WT
ClC-7 KO
Nigericin (20uM, 2hrs):
Apilimod (200nM, 2hrs):
LLOME (1mM, 2hrs):
30kDa
20kDa
pRab10

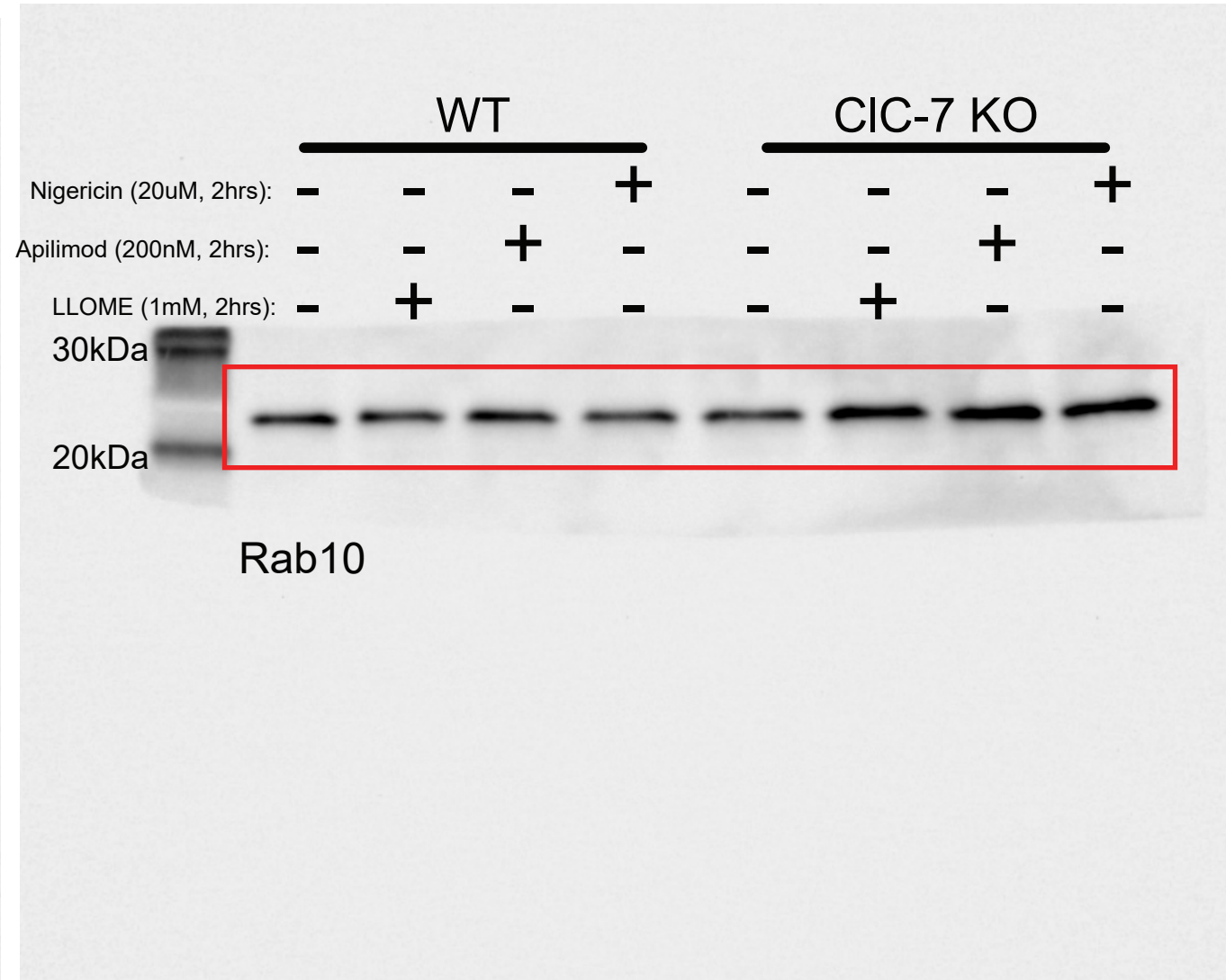
WT
ClC-7 KO
Nigericin (20uM, 2hrs):
Apilimod (200nM, 2hrs):
LLOME (1mM, 2hrs):
30kDa
20kDa
Rab10

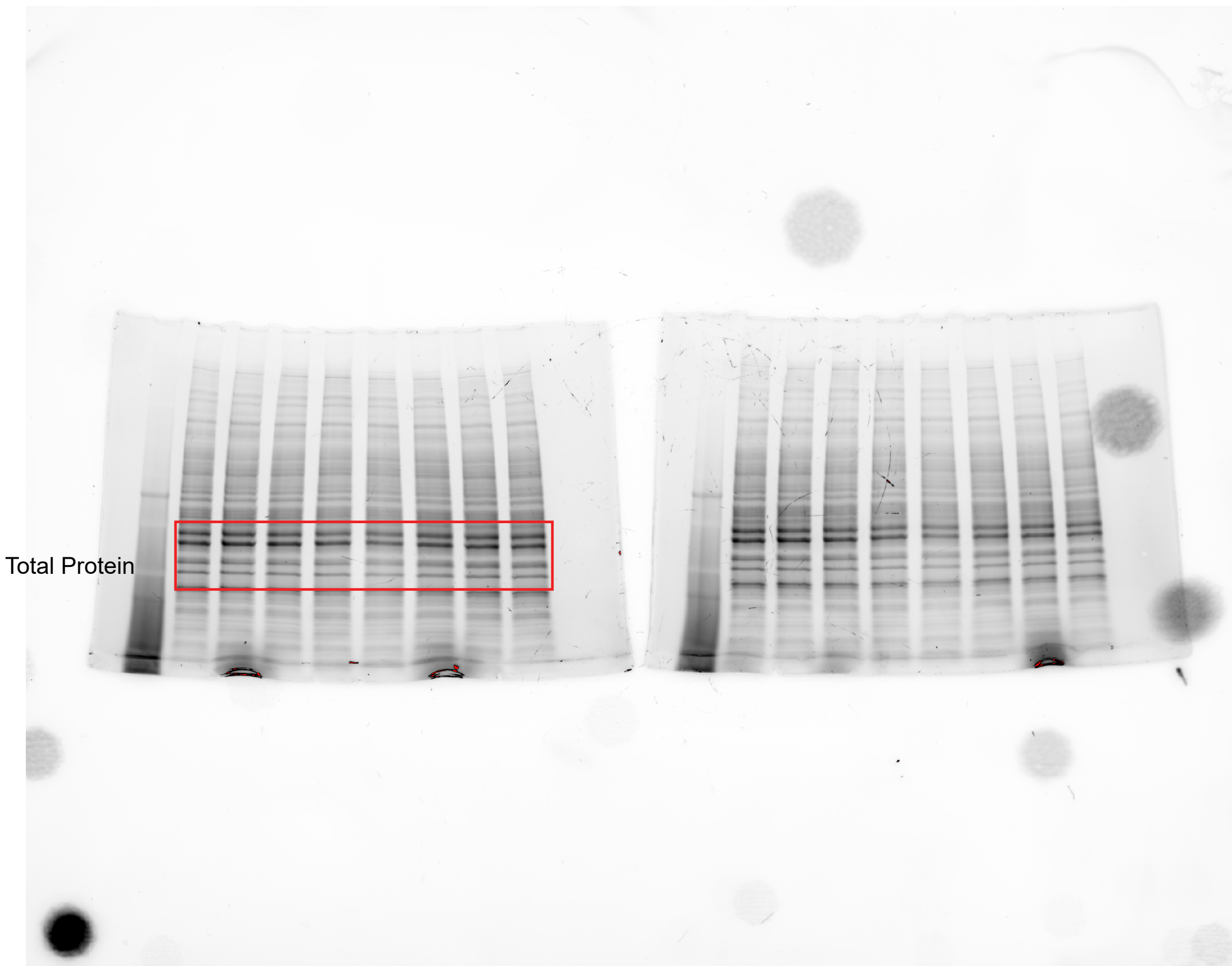
Total Protein

4C

WT vs Atg16L1 KO

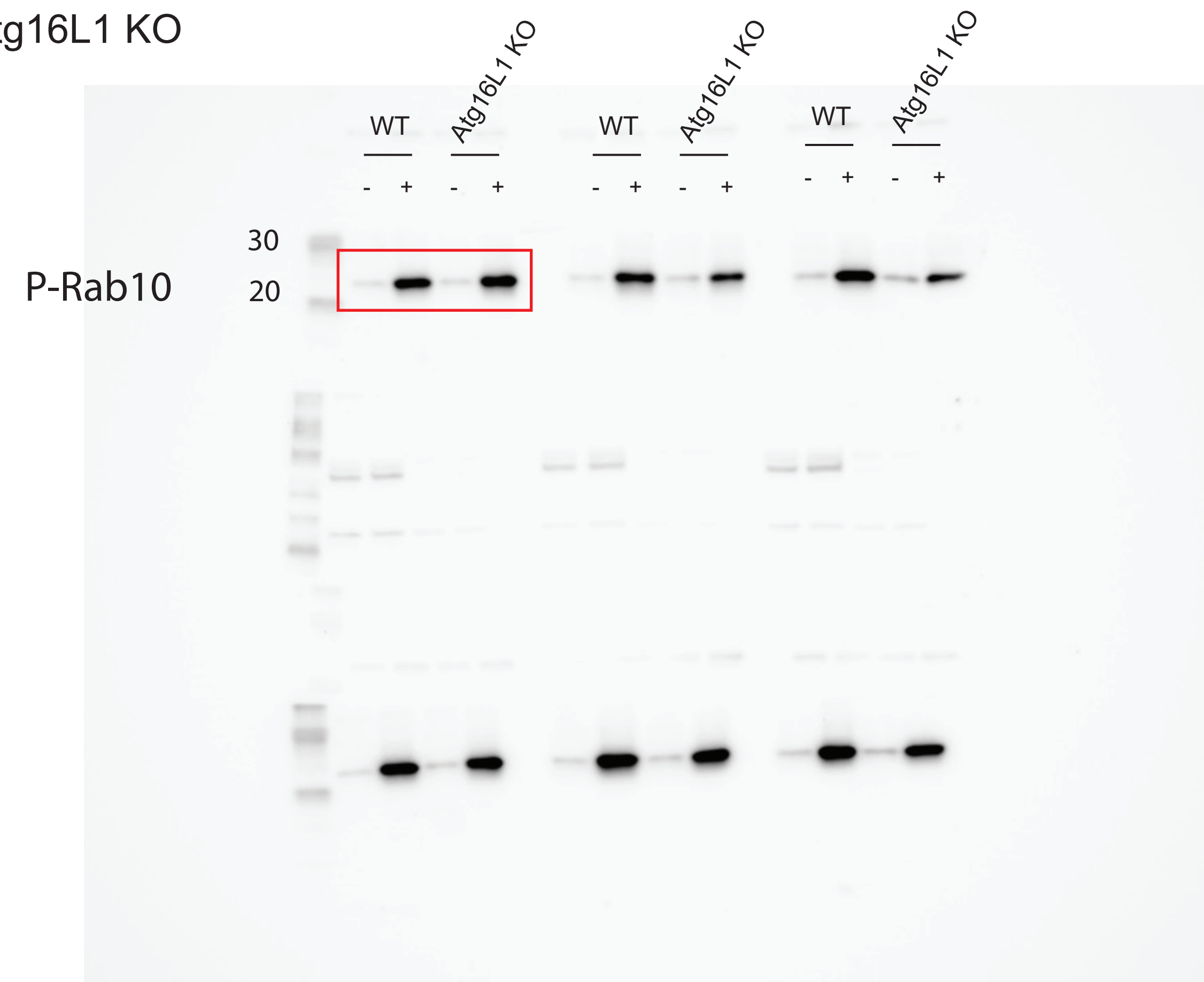

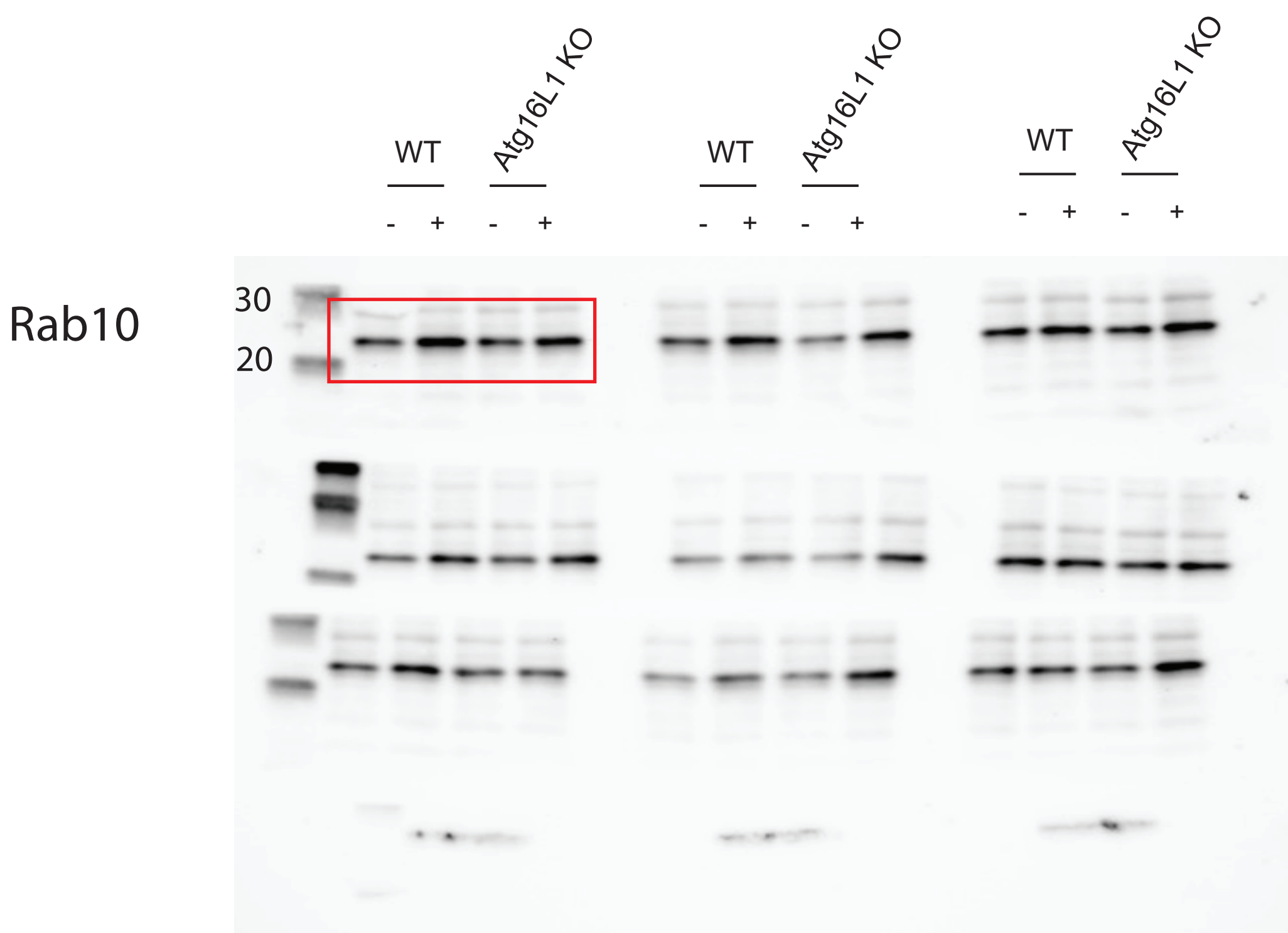

4C

WT vs Atg16L1 KO

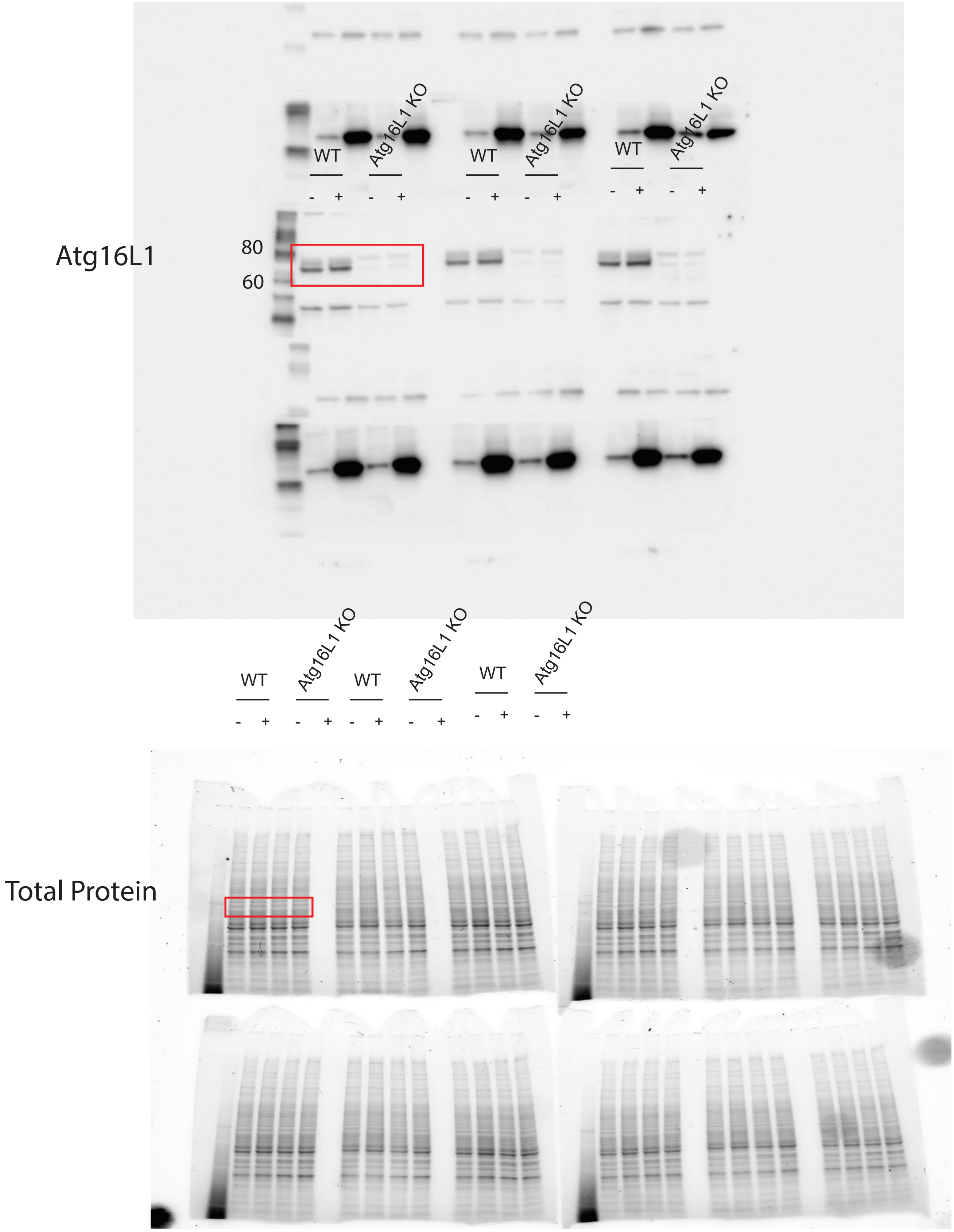

4E

WT vs SopF

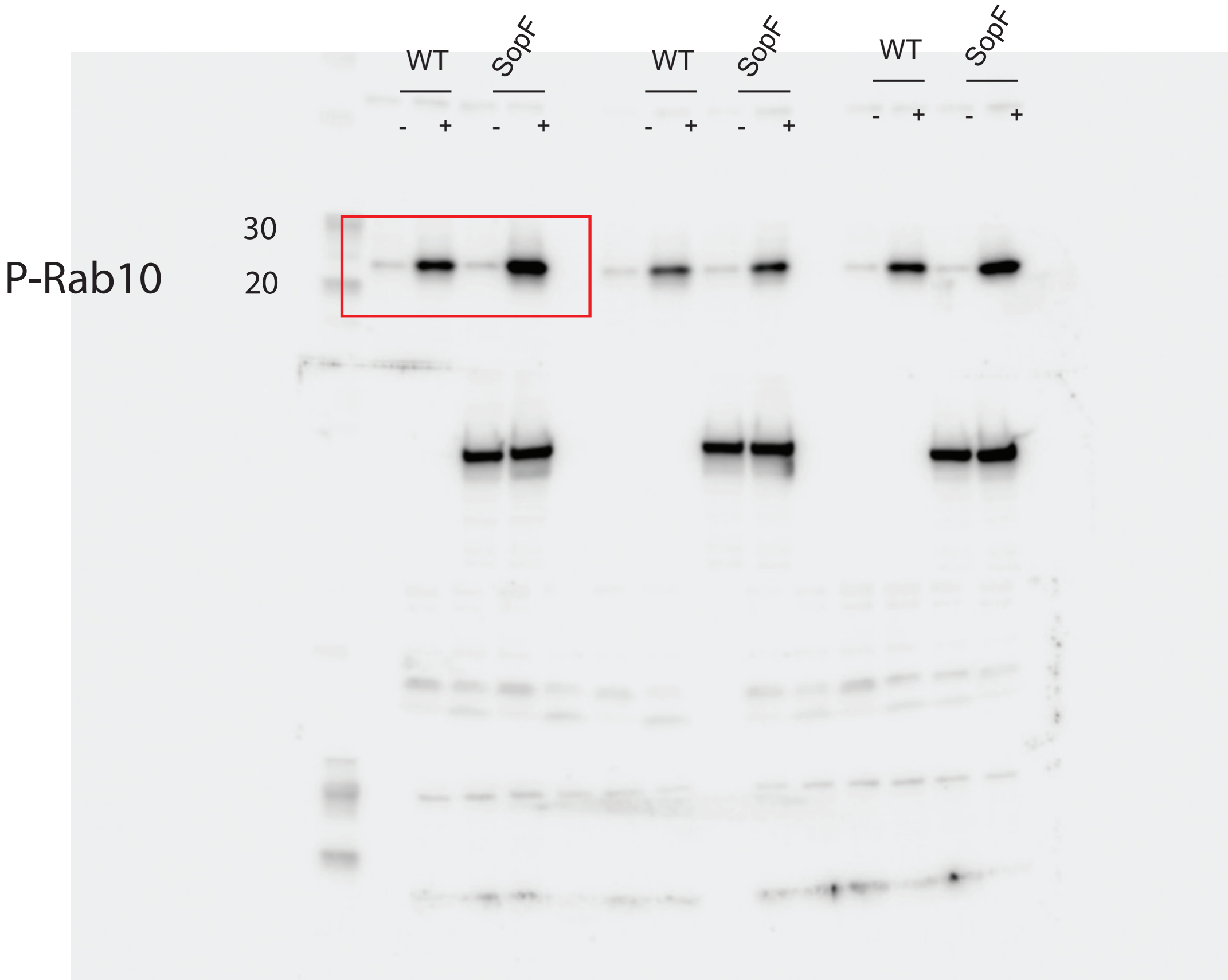
WT
SopF
WT
SopF
WT
SopF
- + - + - + - + - + - +
P-Rab10
30
20

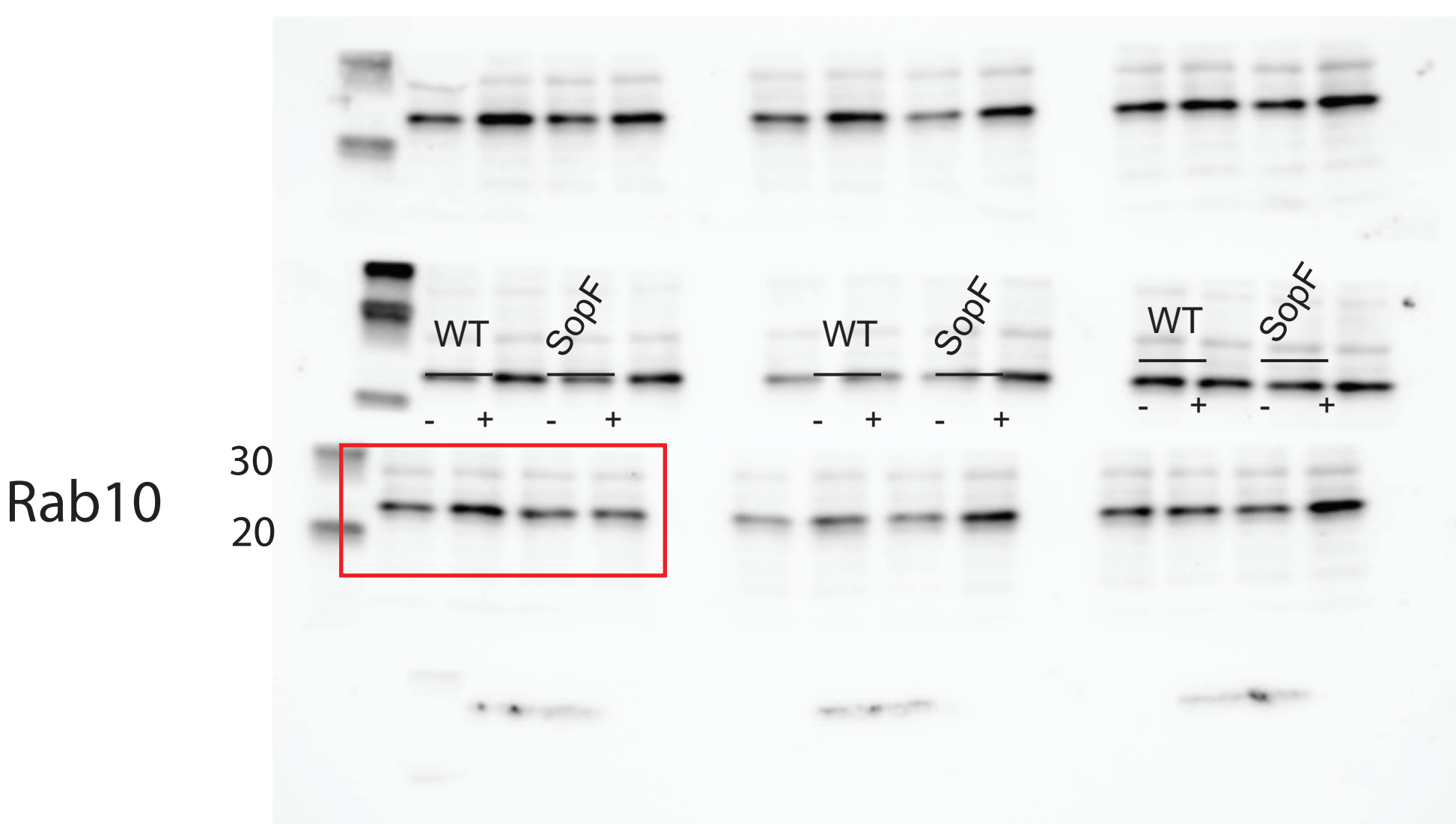
WT
SopF
WT
SopF
WT
SopF
- + - + - + - + - + - +
Rab10
30
20

4E

WT vs SopF

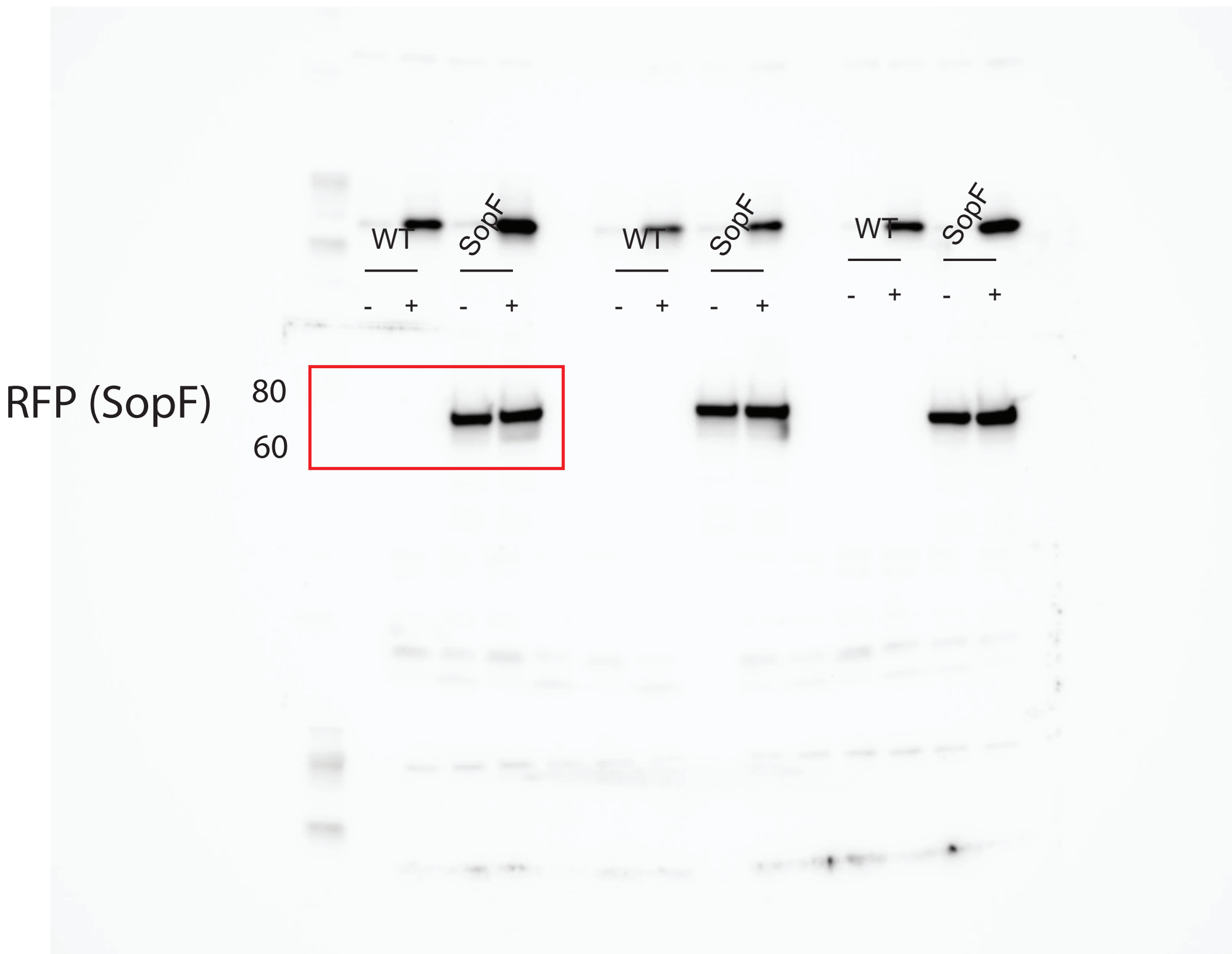
WT
SopF
WT
SopF
WT
SopF
- + - +
- + - +
- + - +
RFP (SopF)
80
60

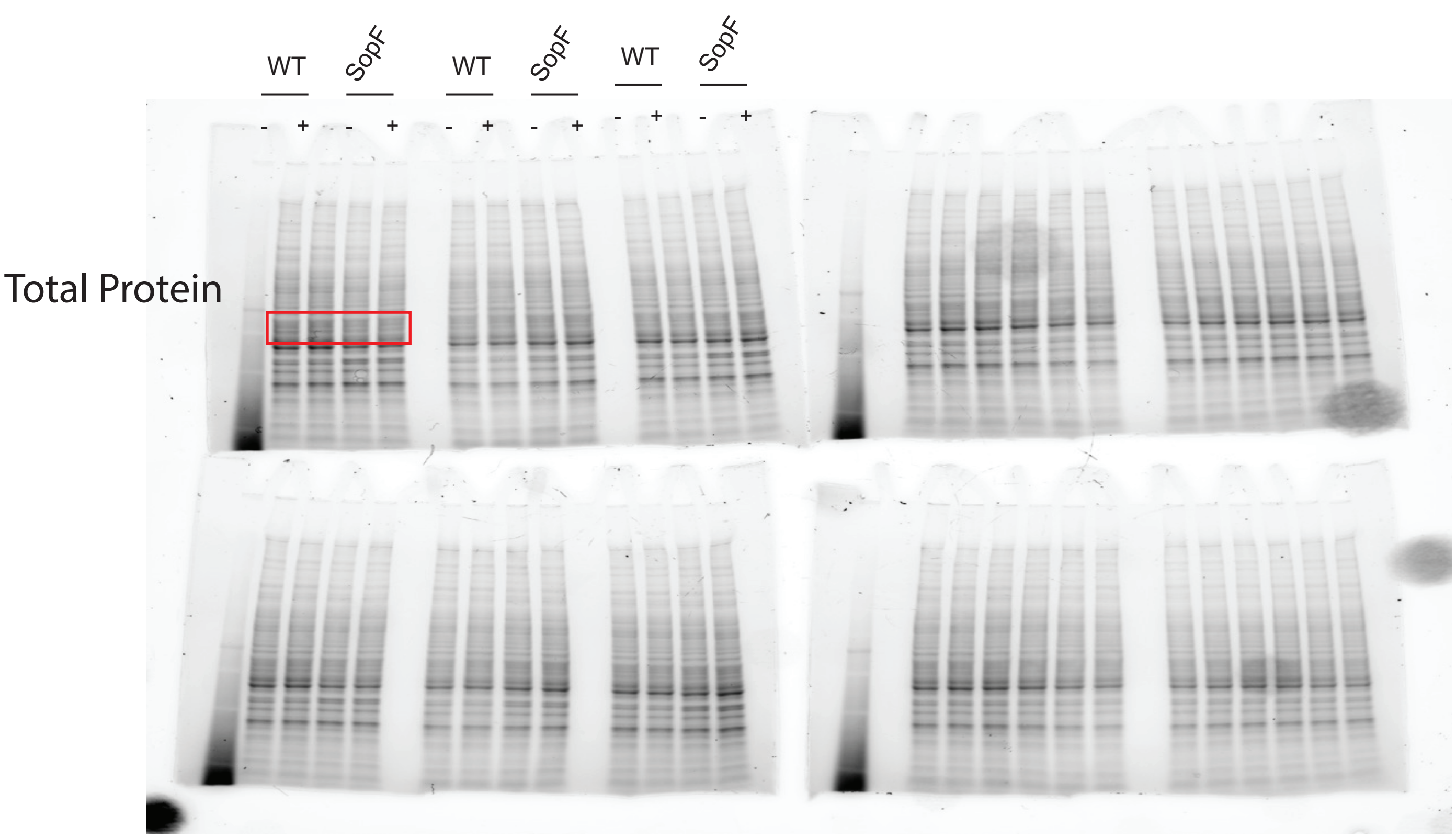
WT
SopF
WT
SopF
WT
SopF
- + - +
- + - +
- + - +
Total Protein

4G

WT vs Gabarap KO

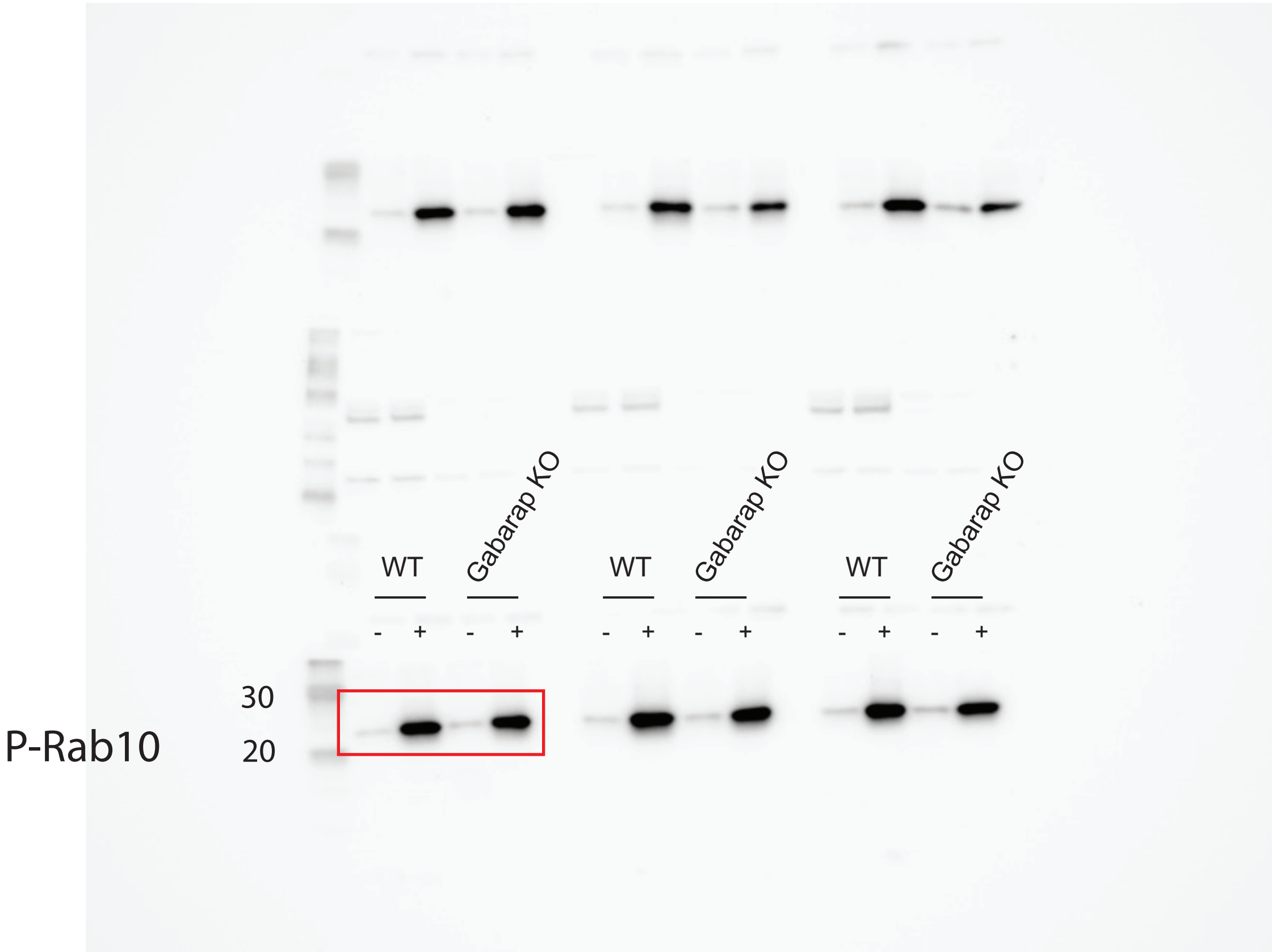

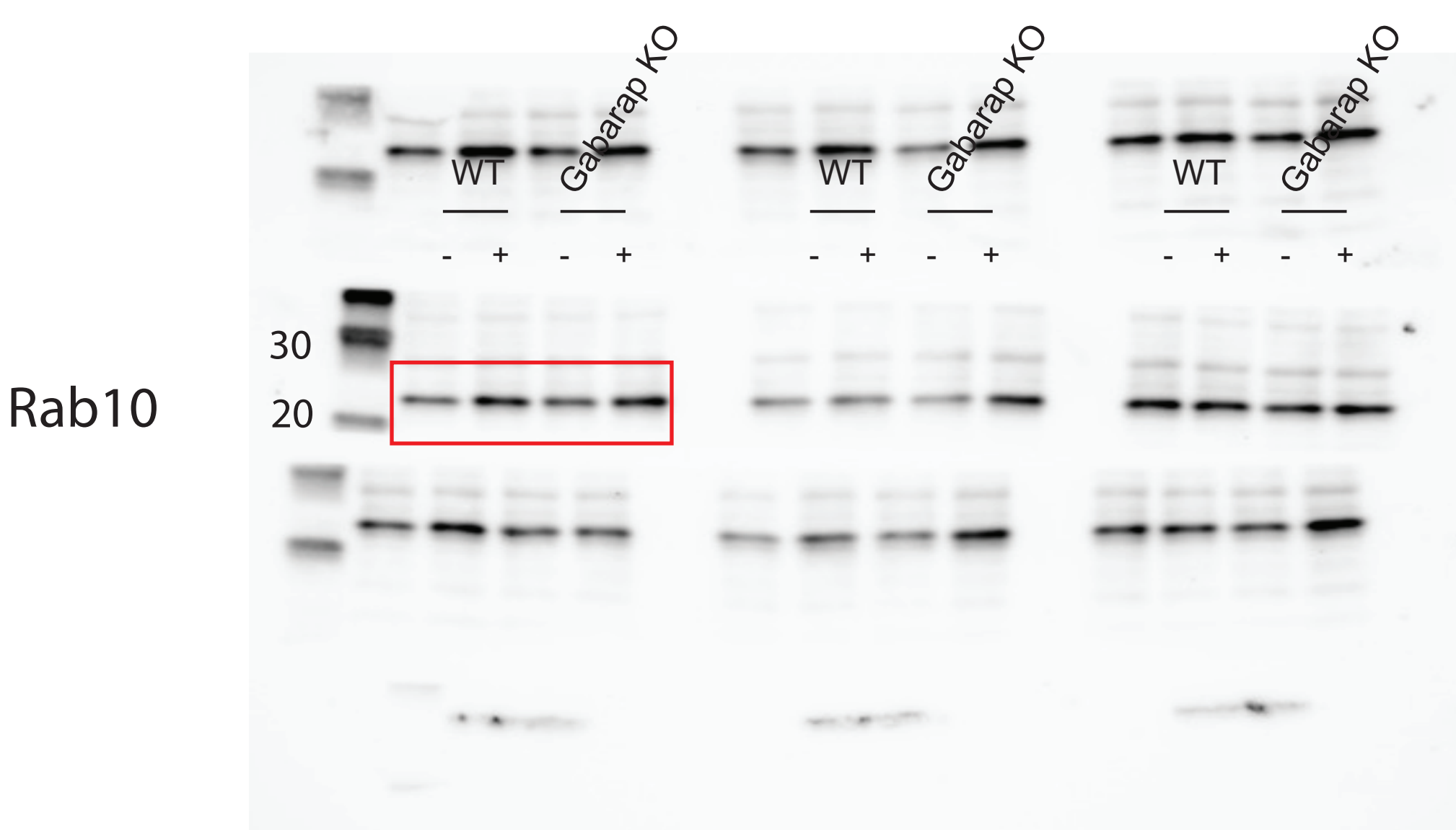

4G

WT vs Gabarap KO

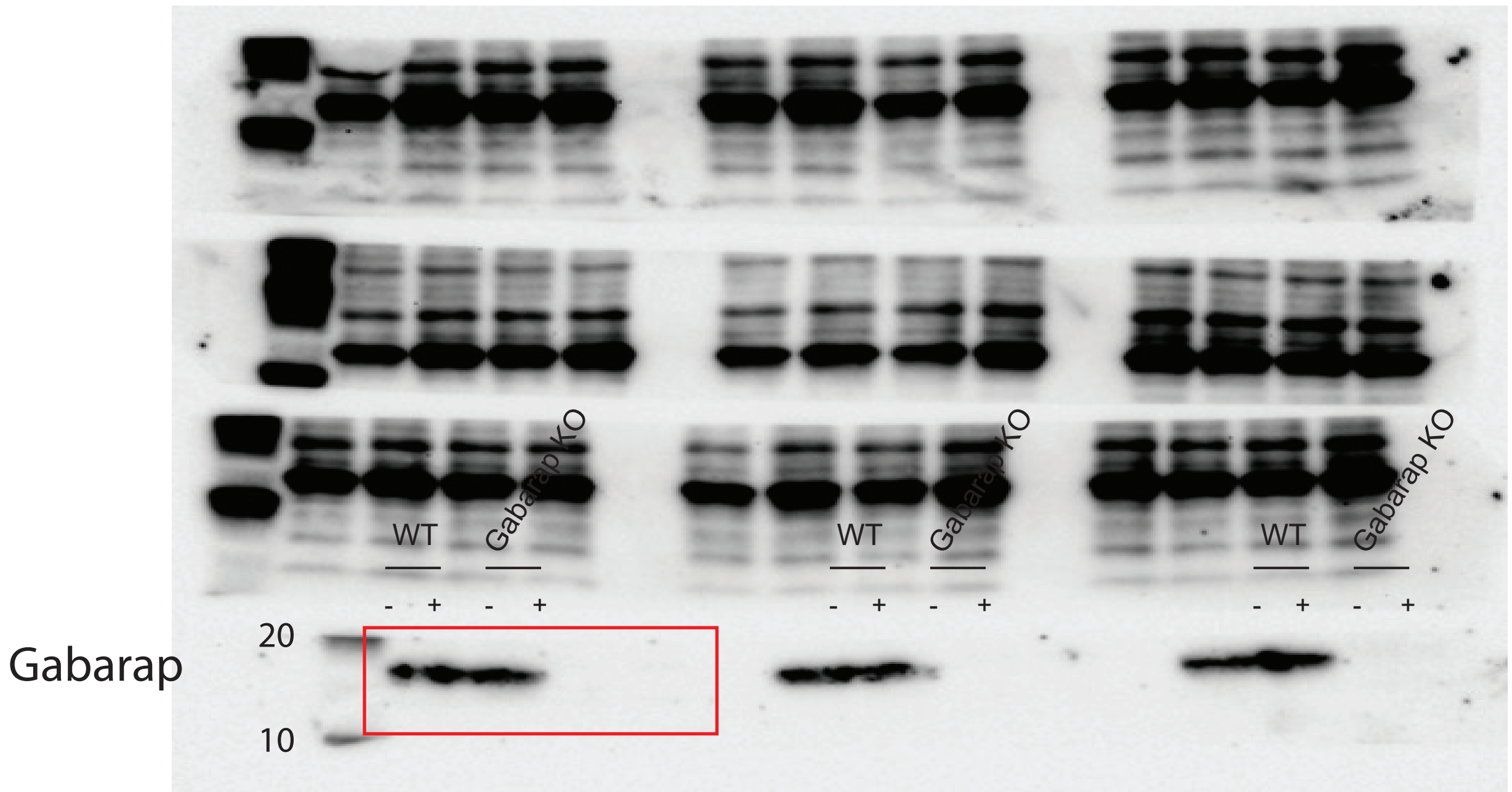

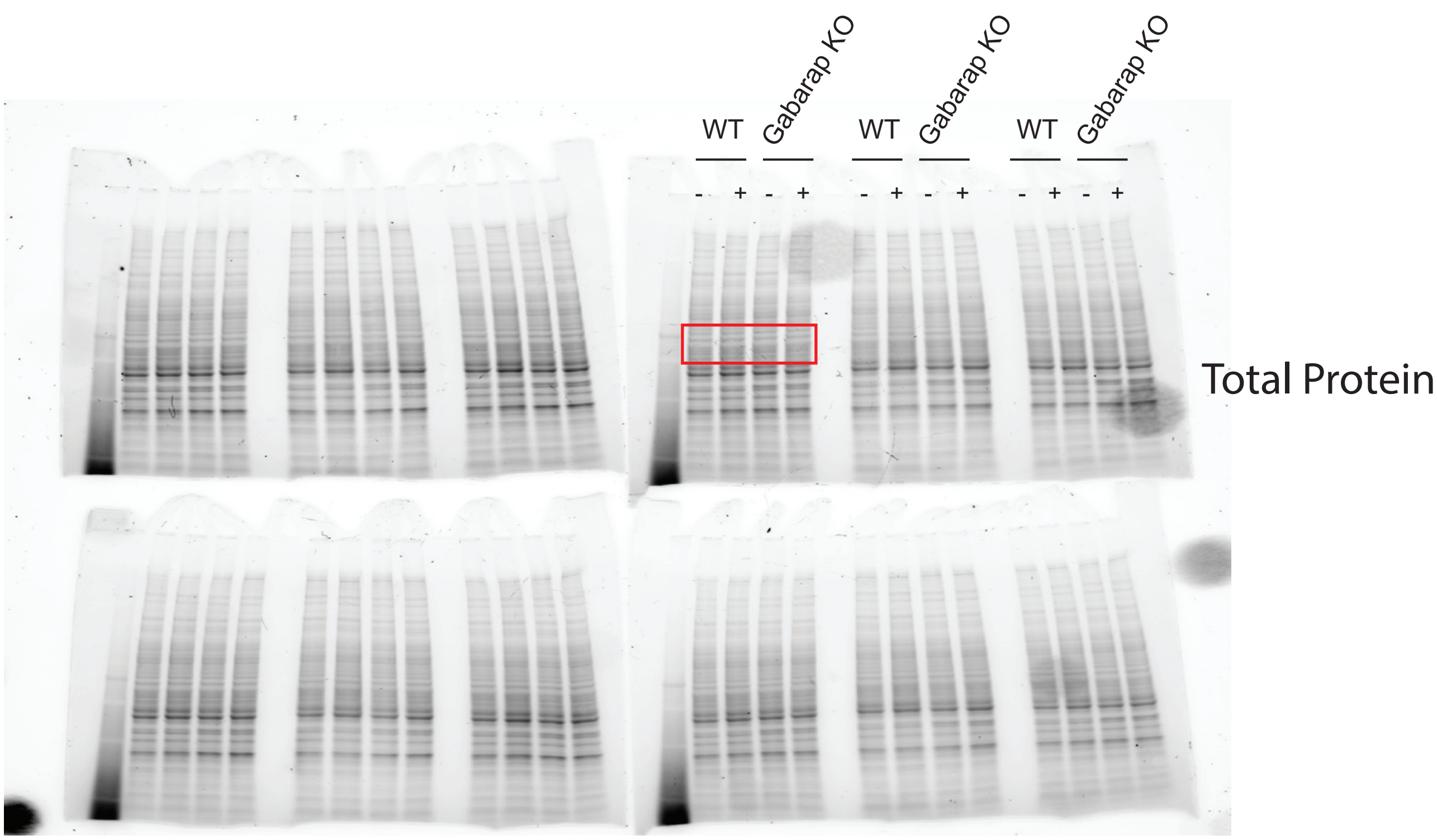

4I

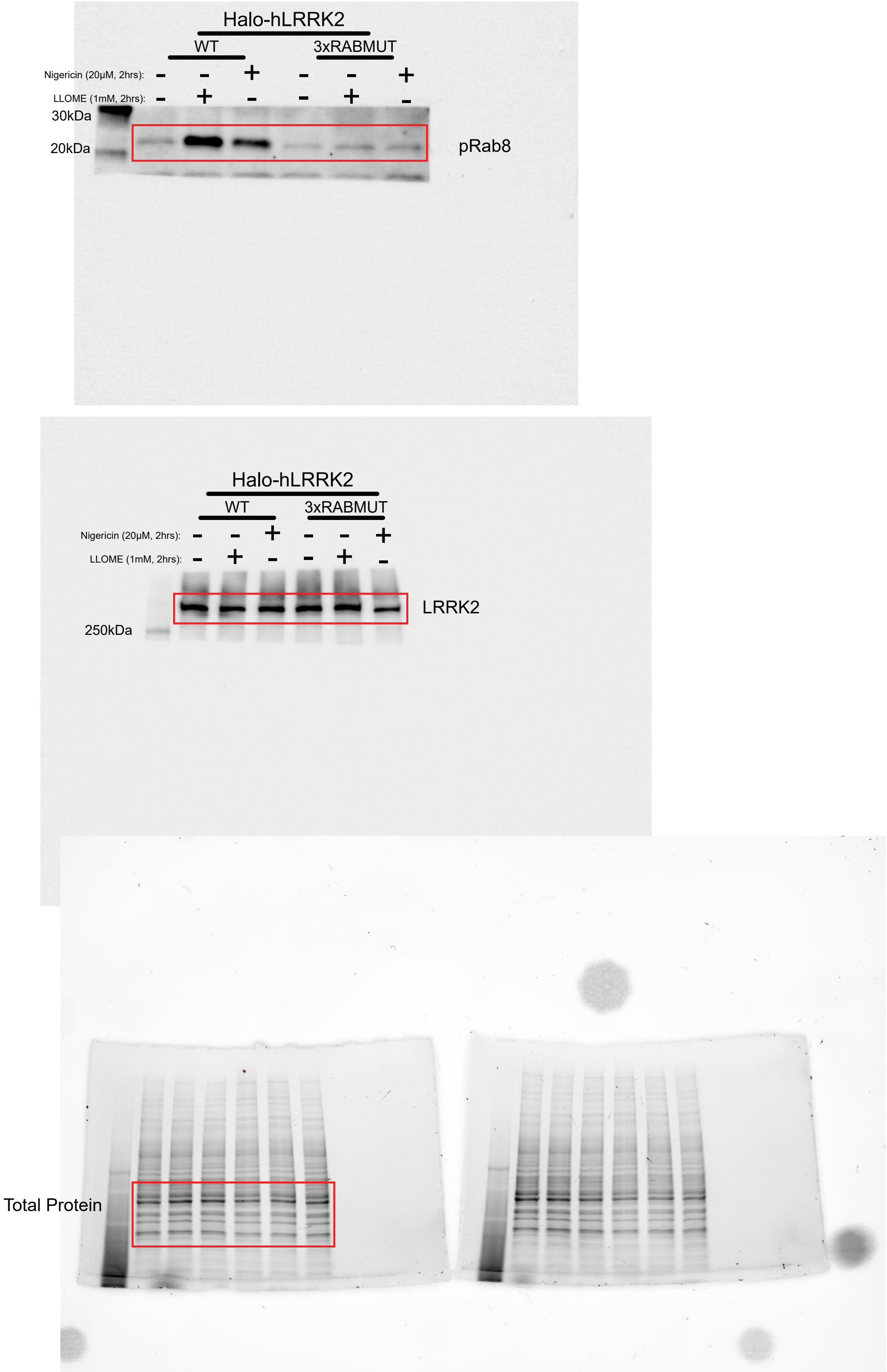
Halo-hLRRK2
WT
3xRABMUT
Nigericin (20μM, 2hrs):
LLOME (1mM, 2hrs):
30kDa
20kDa
pRab8
Halo-hLRRK2
WT
3xRABMUT
Nigericin (20μM, 2hrs):
LLOME (1mM, 2hrs):
250kDa
LRRK2
Total Protein

4I

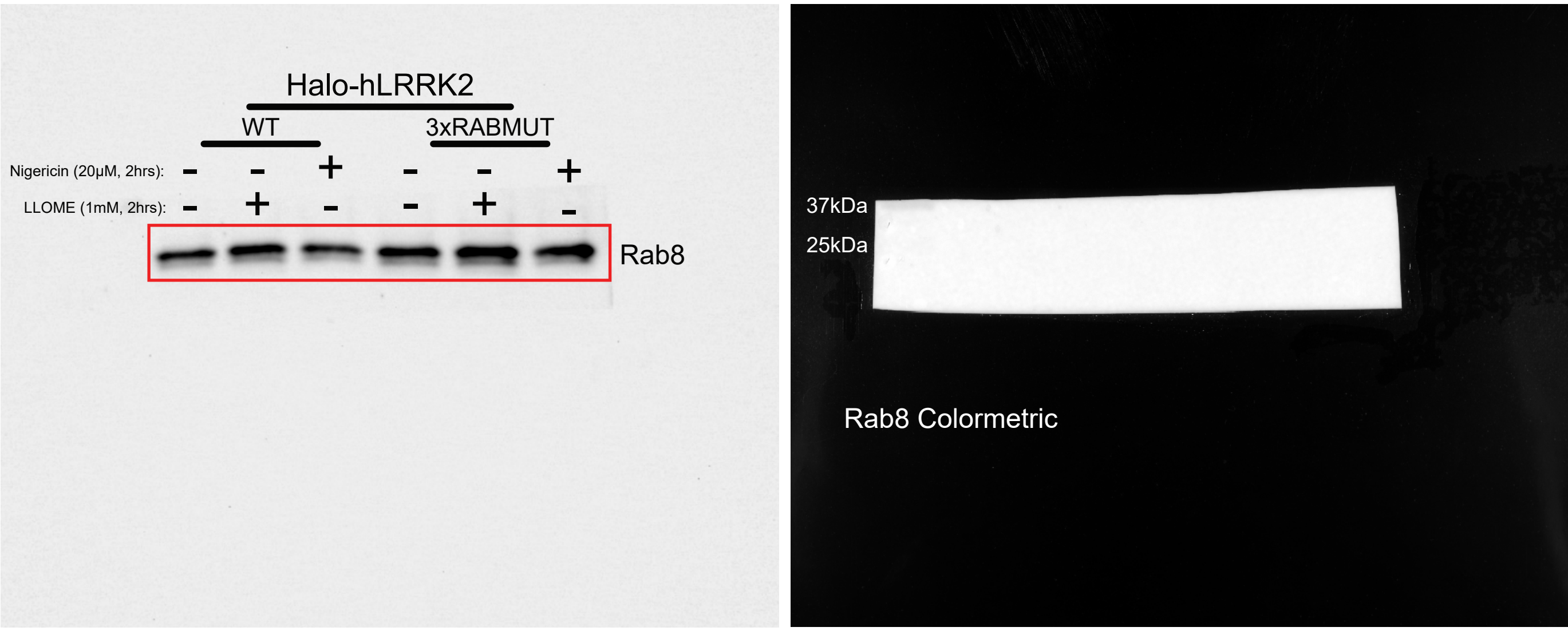
Halo-hLRRK2
WT
3xRABMUT
Nigericin (20μM, 2hrs):
LLOME (1mM, 2hrs):
Rab8
37kDa
25kDa
Rab8 Colormetric

4K

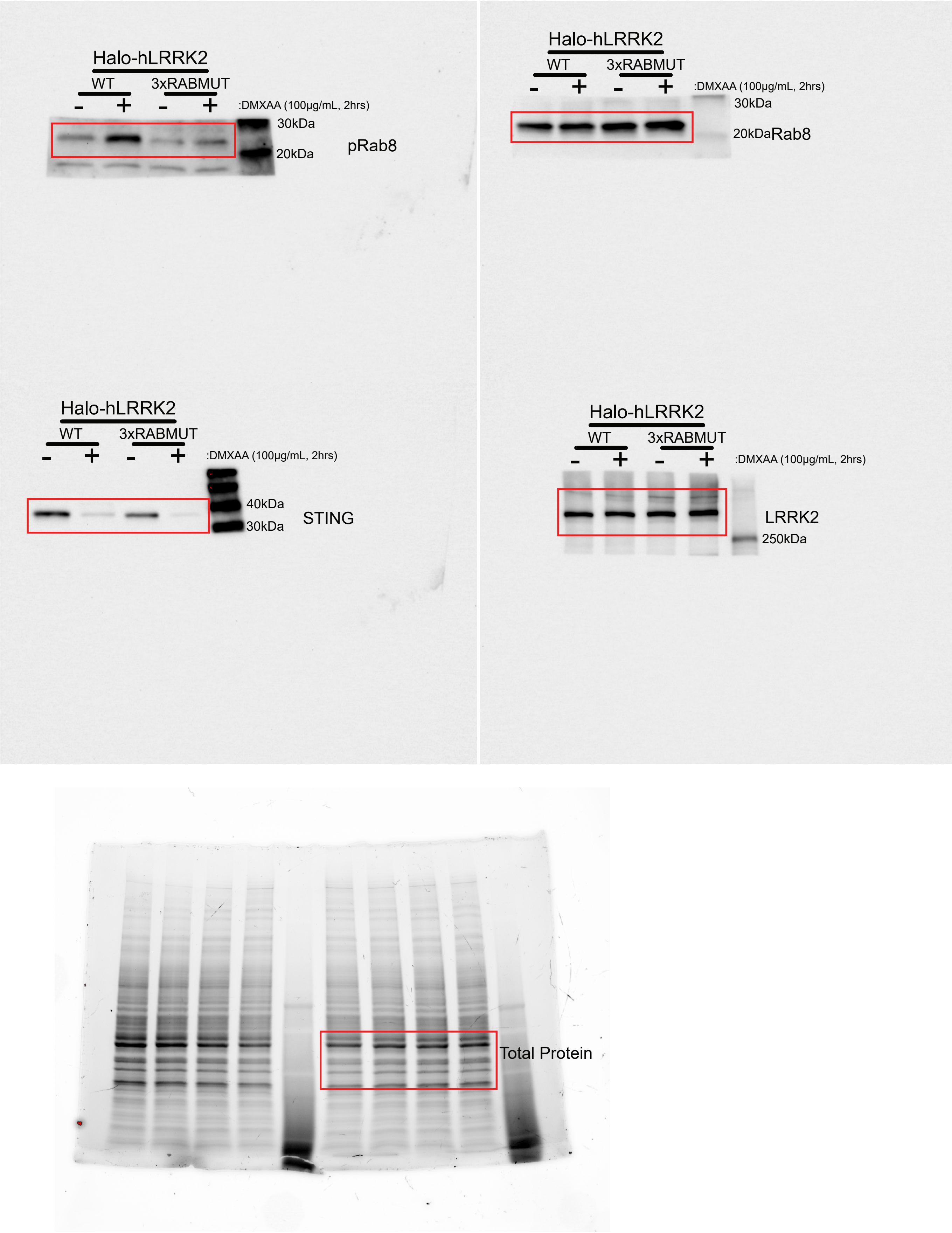
Halo-hLRRK2
WT
3xRABMUT
- + - +
:DMXAA (100µg/mL, 2hrs)
30kDa
20kDa
pRab8
Halo-hLRRK2
WT
3xRABMUT
- + - +
:DMXAA (100µg/mL, 2hrs)
30kDa
20kDa
Rab8
Halo-hLRRK2
WT
3xRABMUT
- + - +
:DMXAA (100µg/mL, 2hrs)
40kDa
30kDa
STING
Halo-hLRRK2
WT
3xRABMUT
- + - +
:DMXAA (100µg/mL, 2hrs)
LRRK2
250kDa
Total Protein
