# Supplementary material for: ClC-7 links PIKfyve inhibition to Rab-dependent LRRK2 activity at lysosomes
Source: J Cell Biol. 2026 Oct 20;225(11):e202511137. doi: 10.1083/jcb.202511137 (PMC13682606; doi:10.1083/jcb.202511137)

5A.

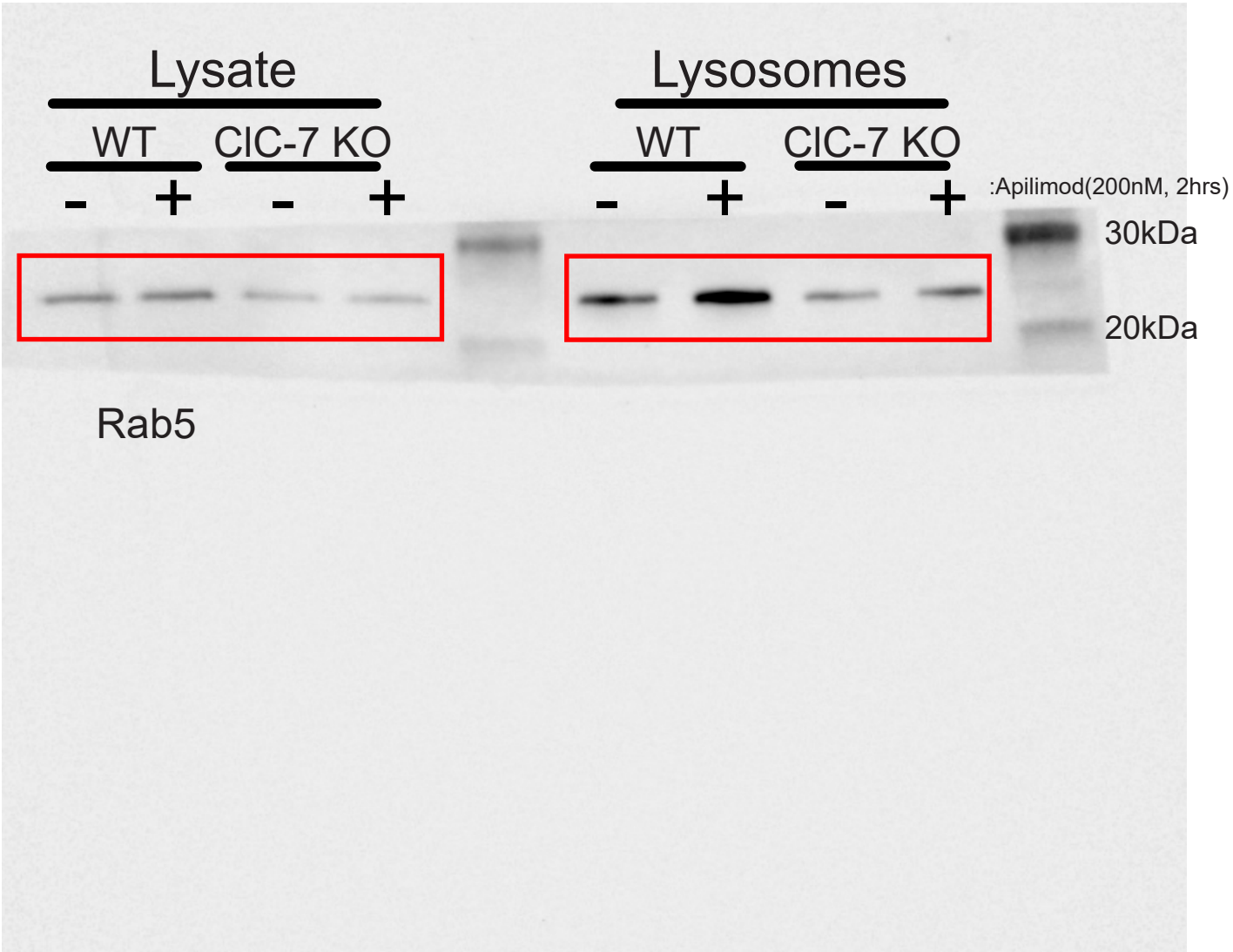
Lysate
Lysosomes
WT
ClC-7 KO
WT
ClC-7 KO
- + - +
- + - +
:Apilimod(200nM, 2hrs)
30kDa
20kDa
Rab5

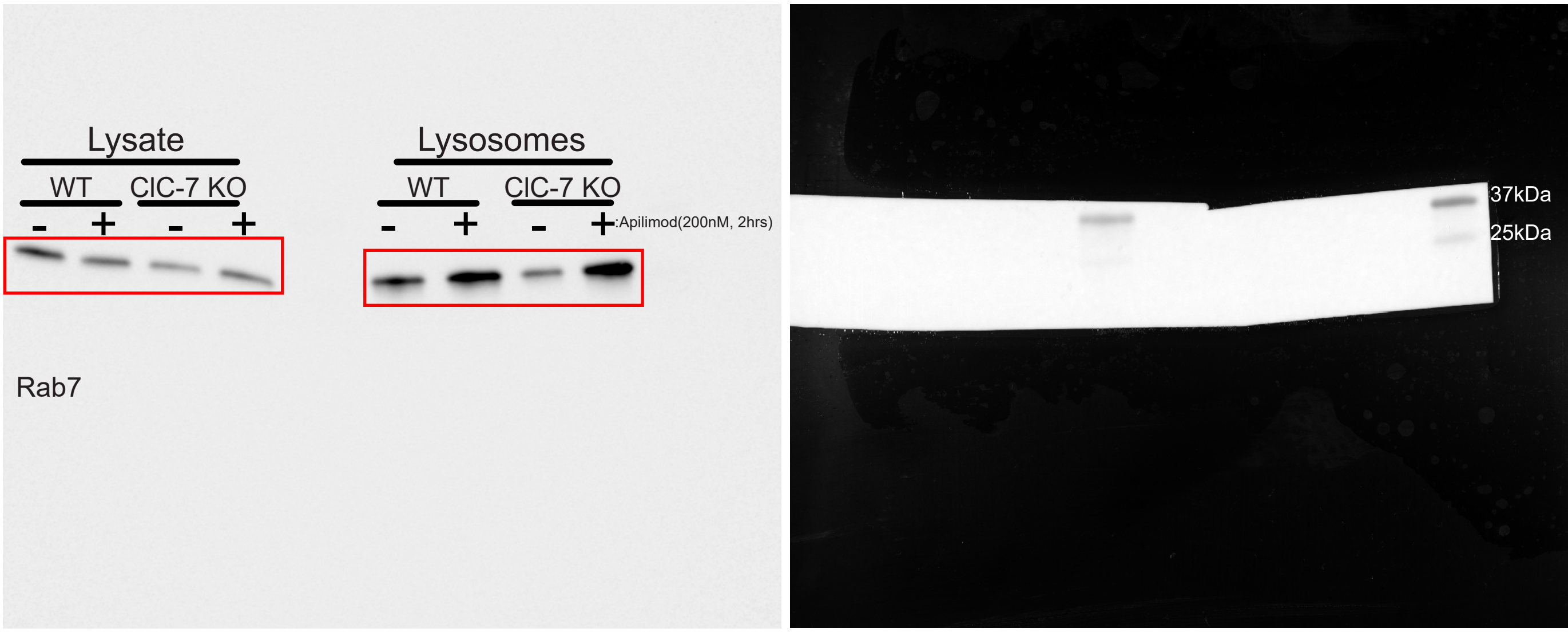
Lysate
Lysosomes
WT
ClC-7 KO
WT
ClC-7 KO
- + - +
- + - +
:Apilimod(200nM, 2hrs)
Rab7
37kDa
25kDa

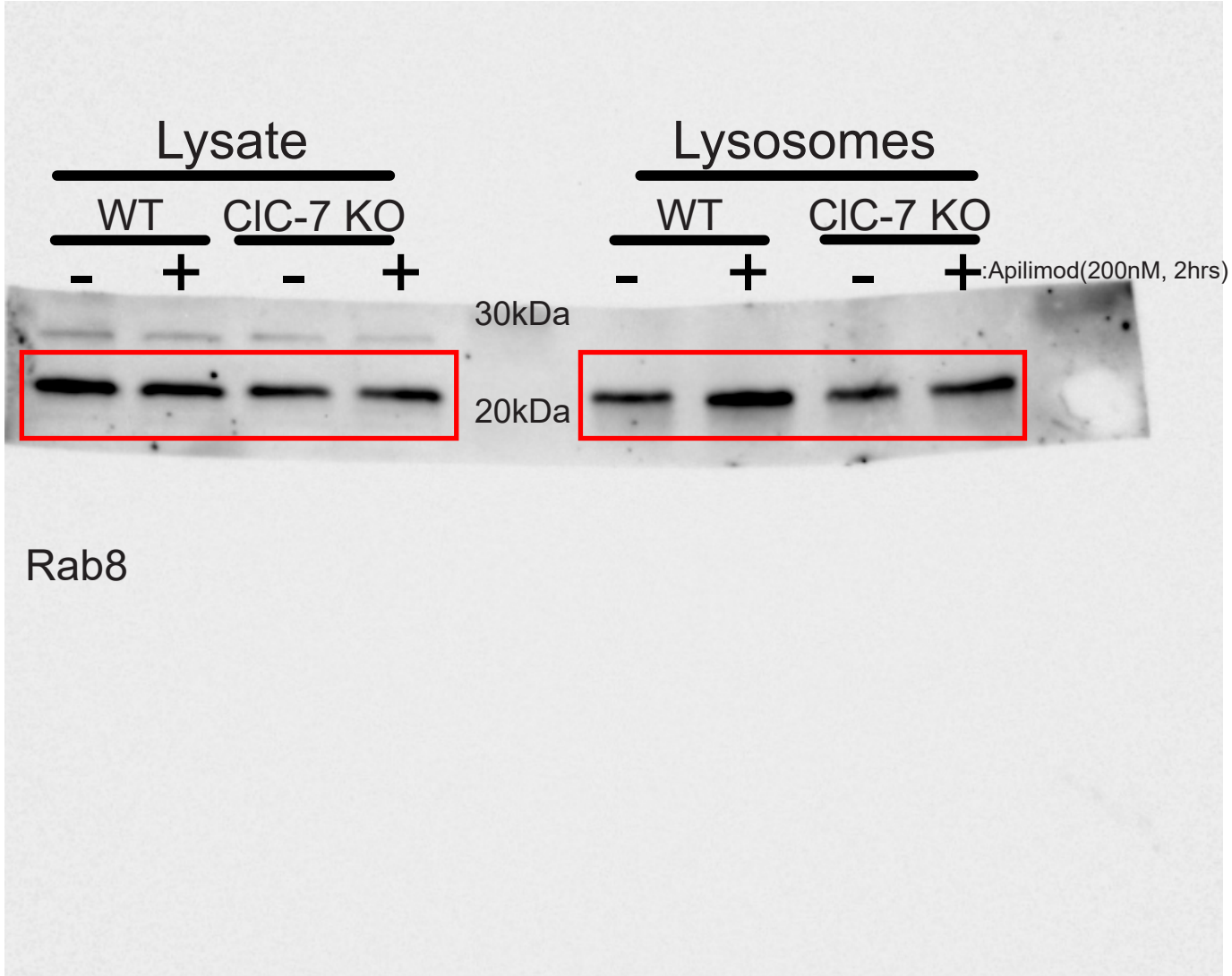
Lysate
Lysosomes
WT
ClC-7 KO
WT
ClC-7 KO
- + - +
- + - +
:Apilimod(200nM, 2hrs)
30kDa
20kDa
Rab8

5E.

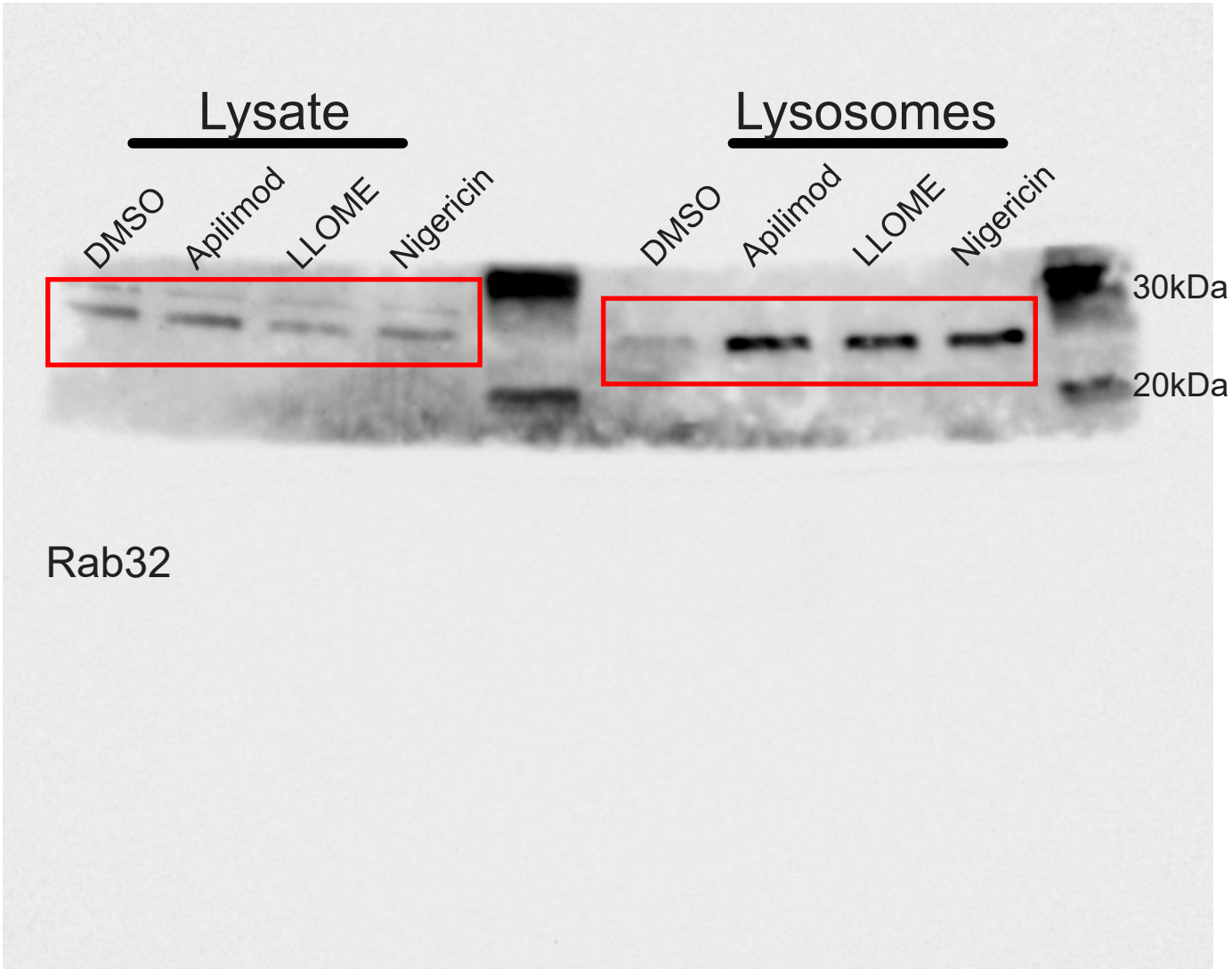
Lysate
Lysosomes
DMSO
Apilimod
LLOME
Nigericin
DMSO
Apilimod
LLOME
Nigericin
30kDa
20kDa
Rab32

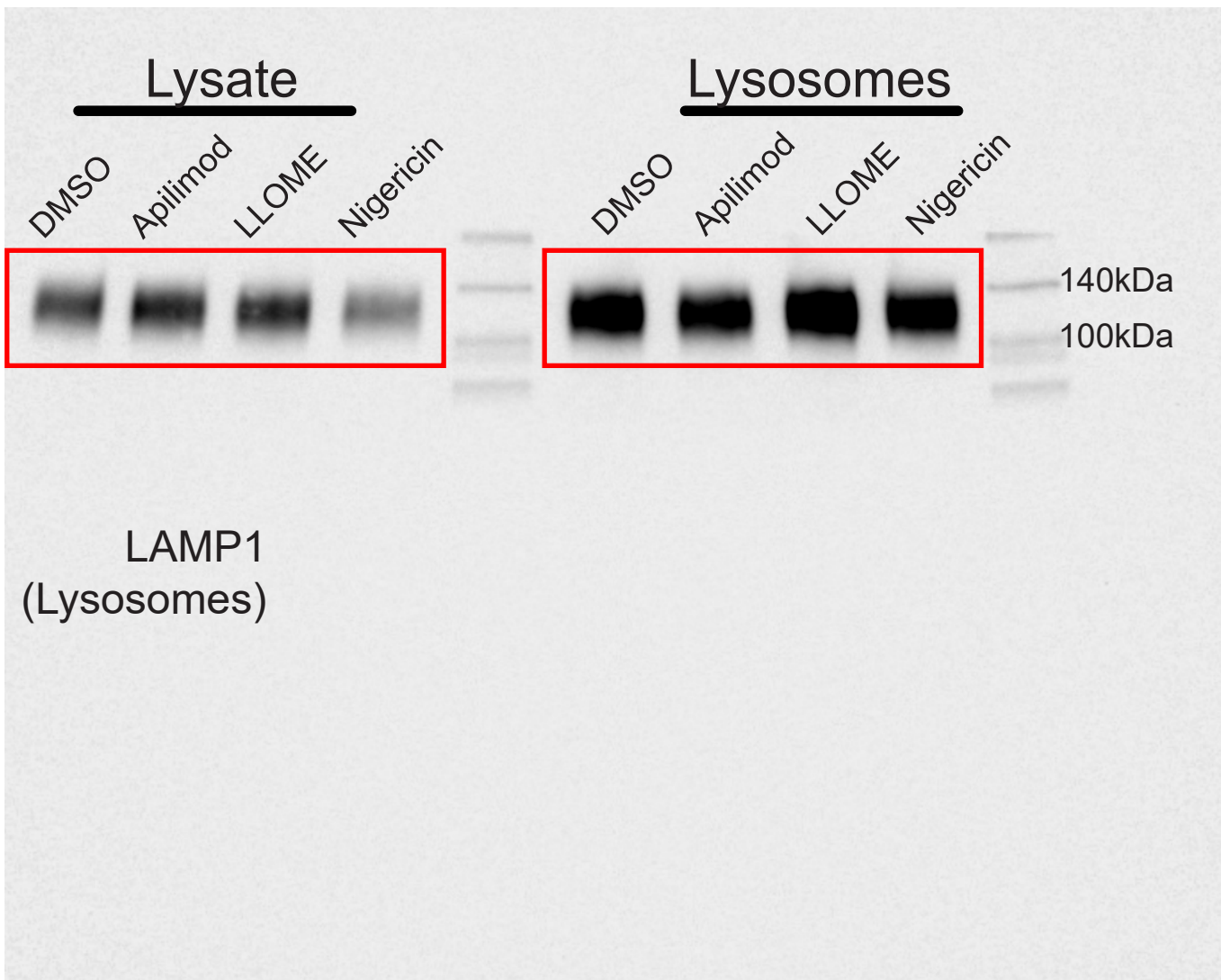
Lysate
Lysosomes
DMSO
Apilimod
LLOME
Nigericin
DMSO
Apilimod
LLOME
Nigericin
140kDa
100kDa
LAMP1
(Lysosomes)

5A.

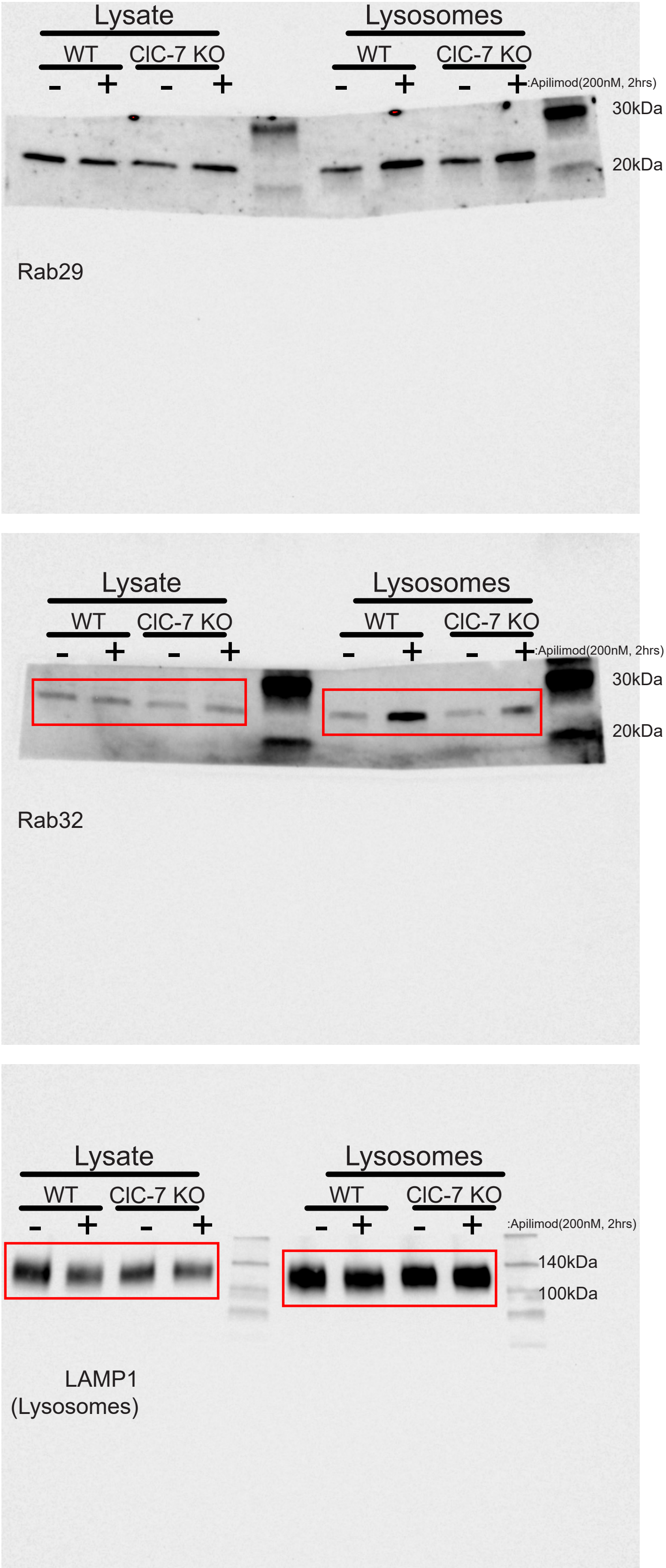

Lysate
Lysosomes
WT
ClC-7 KO
WT
ClC-7 KO
- + - +
- + - +
:Apilimod(200nM, 2hrs)
30kDa
20kDa
Rab29
Lysate
Lysosomes
WT
ClC-7 KO
WT
ClC-7 KO
- + - +
- + - +
:Apilimod(200nM, 2hrs)
30kDa
20kDa
Rab32
Lysate
Lysosomes
WT
ClC-7 KO
WT
ClC-7 KO
- + - +
- + - +
:Apilimod(200nM, 2hrs)
140kDa
100kDa
LAMP1
(Lysosomes)

5A.

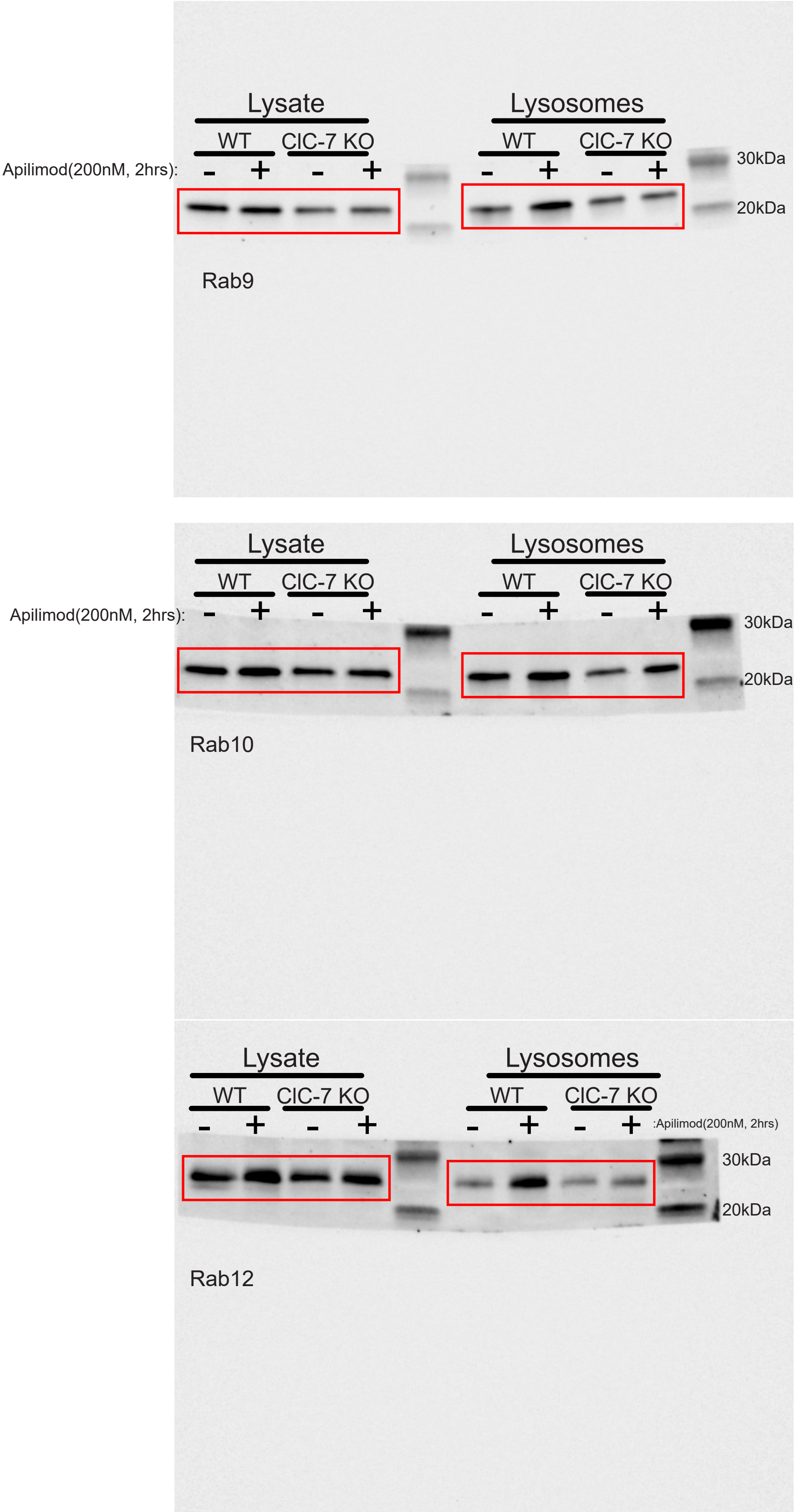

Lysate
WT
ClC-7 KO
Lysosomes
WT
ClC-7 KO
Apilimod(200nM, 2hrs):
- + - +
- + - +
30kDa
20kDa
Rab9
Lysate
WT
ClC-7 KO
Lysosomes
WT
ClC-7 KO
Apilimod(200nM, 2hrs):
- + - +
- + - +
30kDa
20kDa
Rab10
Lysate
WT
ClC-7 KO
Lysosomes
WT
ClC-7 KO
- + - +
- + - +
:Apilimod(200nM, 2hrs)
30kDa
20kDa
Rab12

5E.

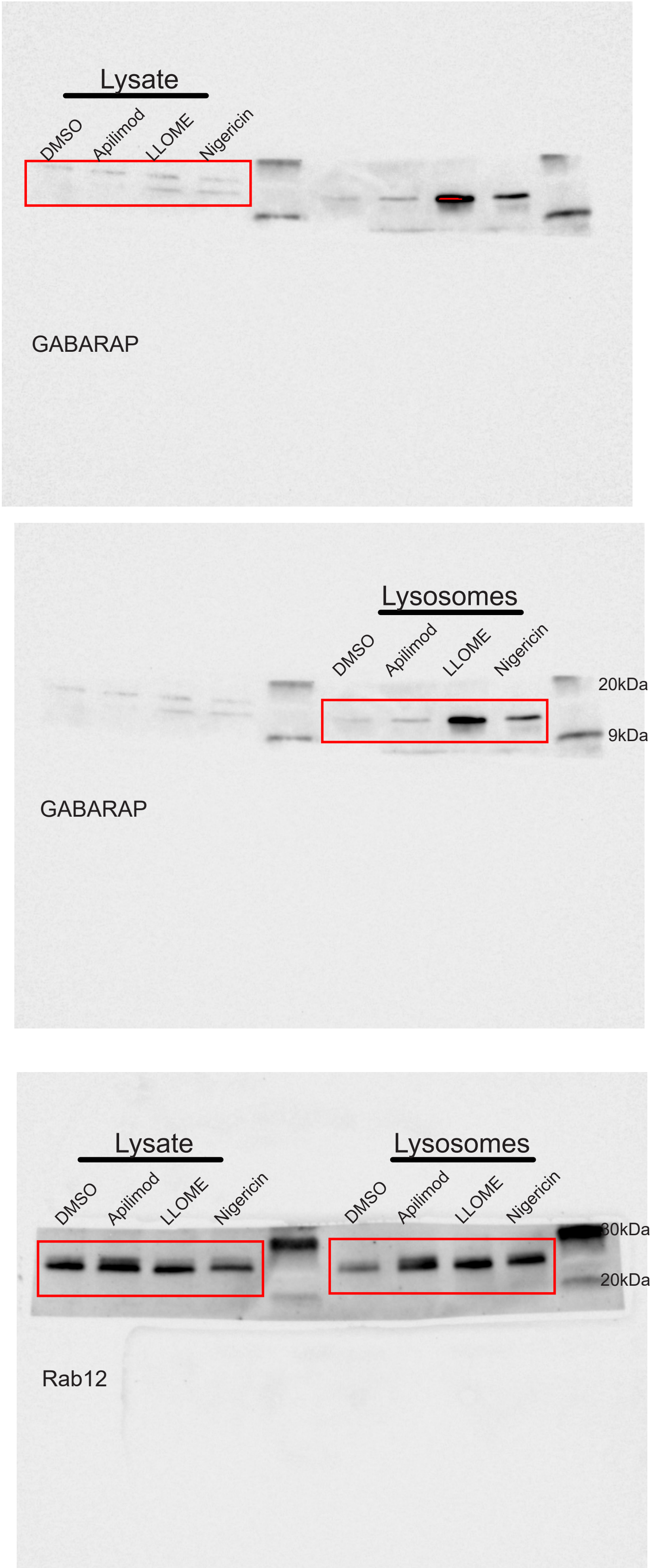

Lysate
DMSO
Apilimod
LLOME
Nigericin
GABARAP
Lysosomes
DMSO
Apilimod
LLOME
Nigericin
20kDa
9kDa
GABARAP
Lysate
DMSO
Apilimod
LLOME
Nigericin
Lysosomes
DMSO
Apilimod
LLOME
Nigericin
30kDa
20kDa
Rab12

5C.

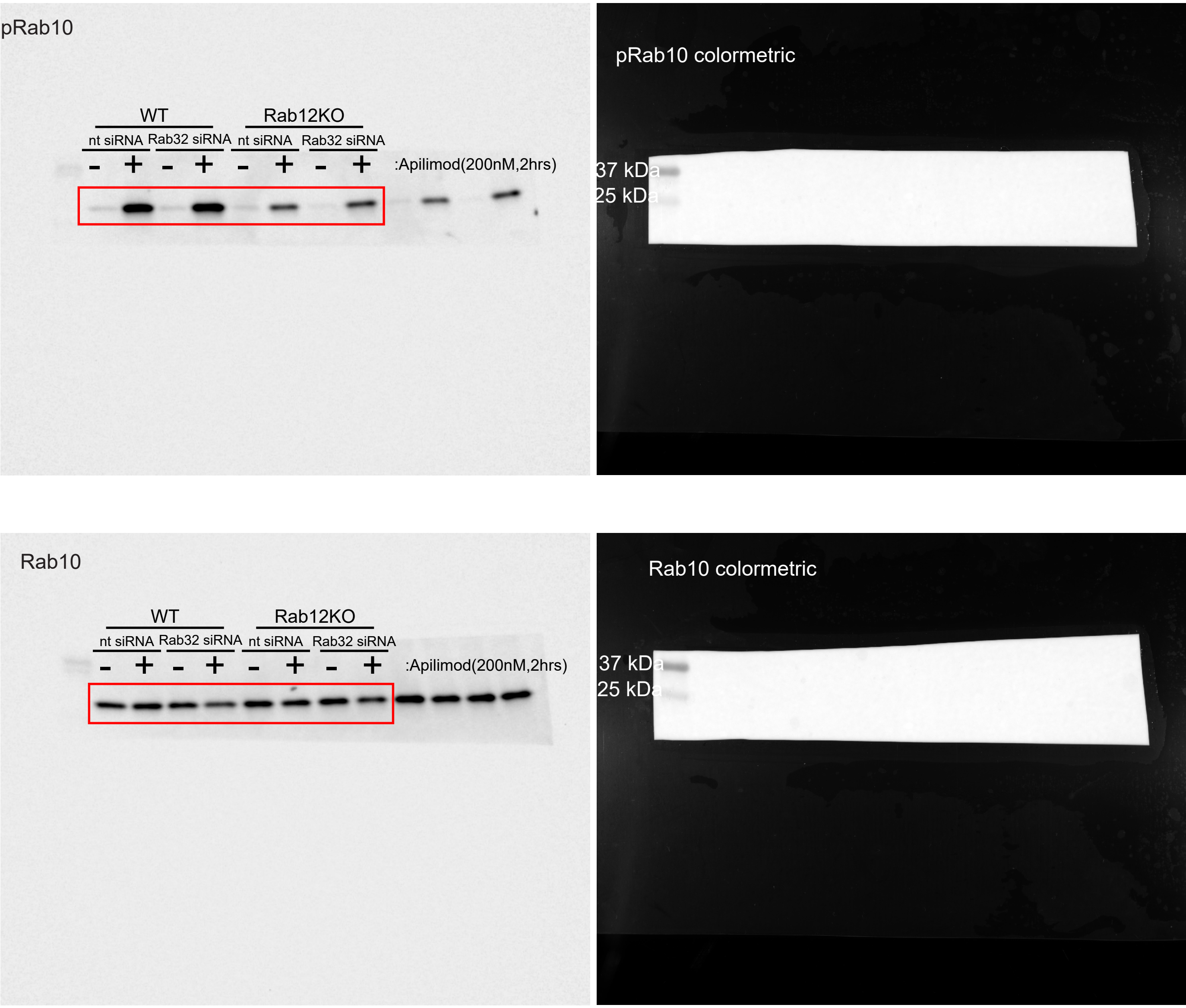

pRab10
WT
Rab12KO
nt siRNA
Rab32 siRNA
nt siRNA
Rab32 siRNA
- + - + - + - +
:Apilimod(200nM,2hrs)
pRab10 colormetric
37 kDa
25 kDa
Rab10
WT
Rab12KO
nt siRNA
Rab32 siRNA
nt siRNA
Rab32 siRNA
- + - + - + - +
:Apilimod(200nM,2hrs)
Rab10 colormetric
37 kDa
25 kDa

5C.

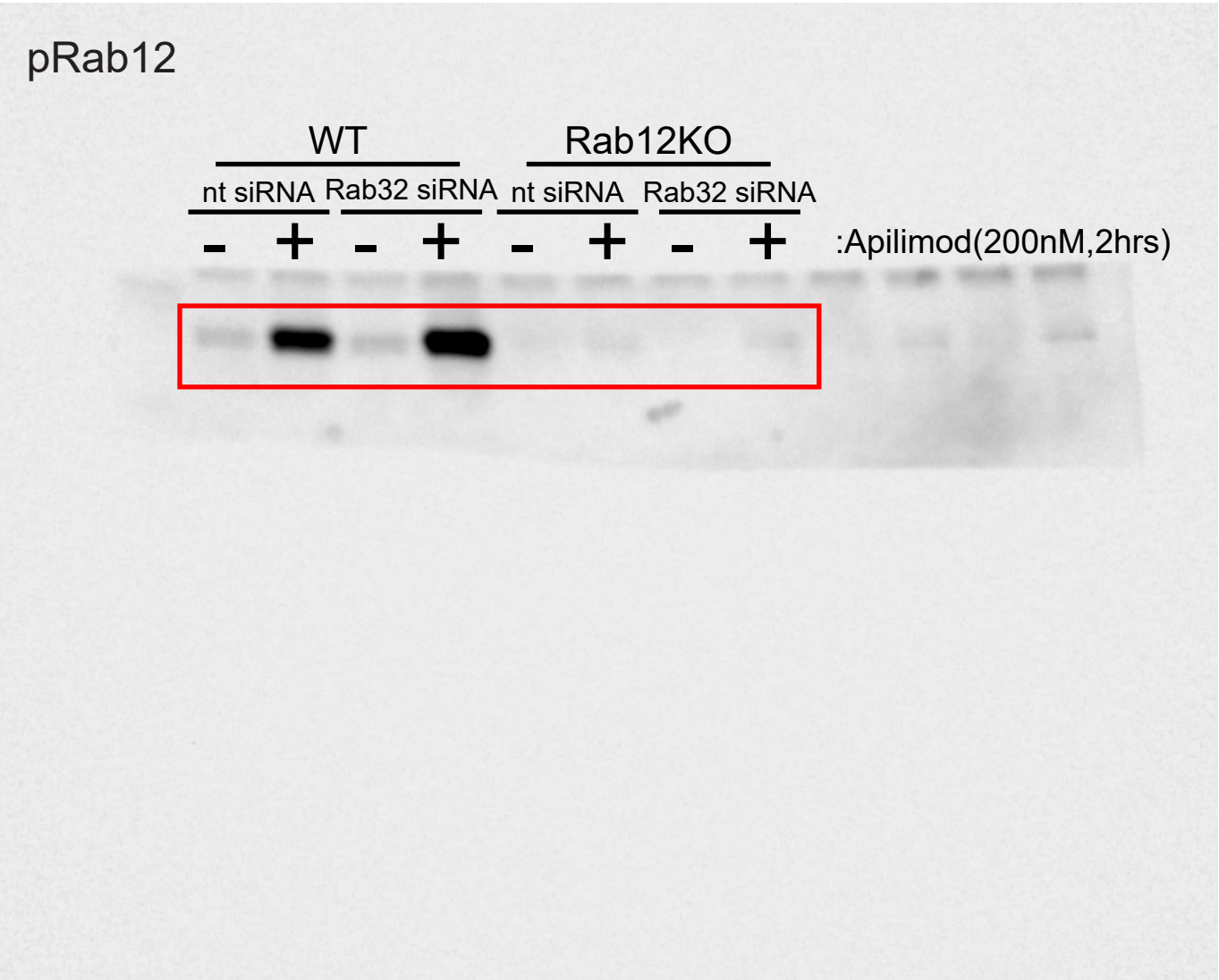
pRab12
WT
Rab12KO
nt siRNA
Rab32 siRNA
nt siRNA
Rab32 siRNA
- + - + - + - +
:Apilimod(200nM,2hrs)

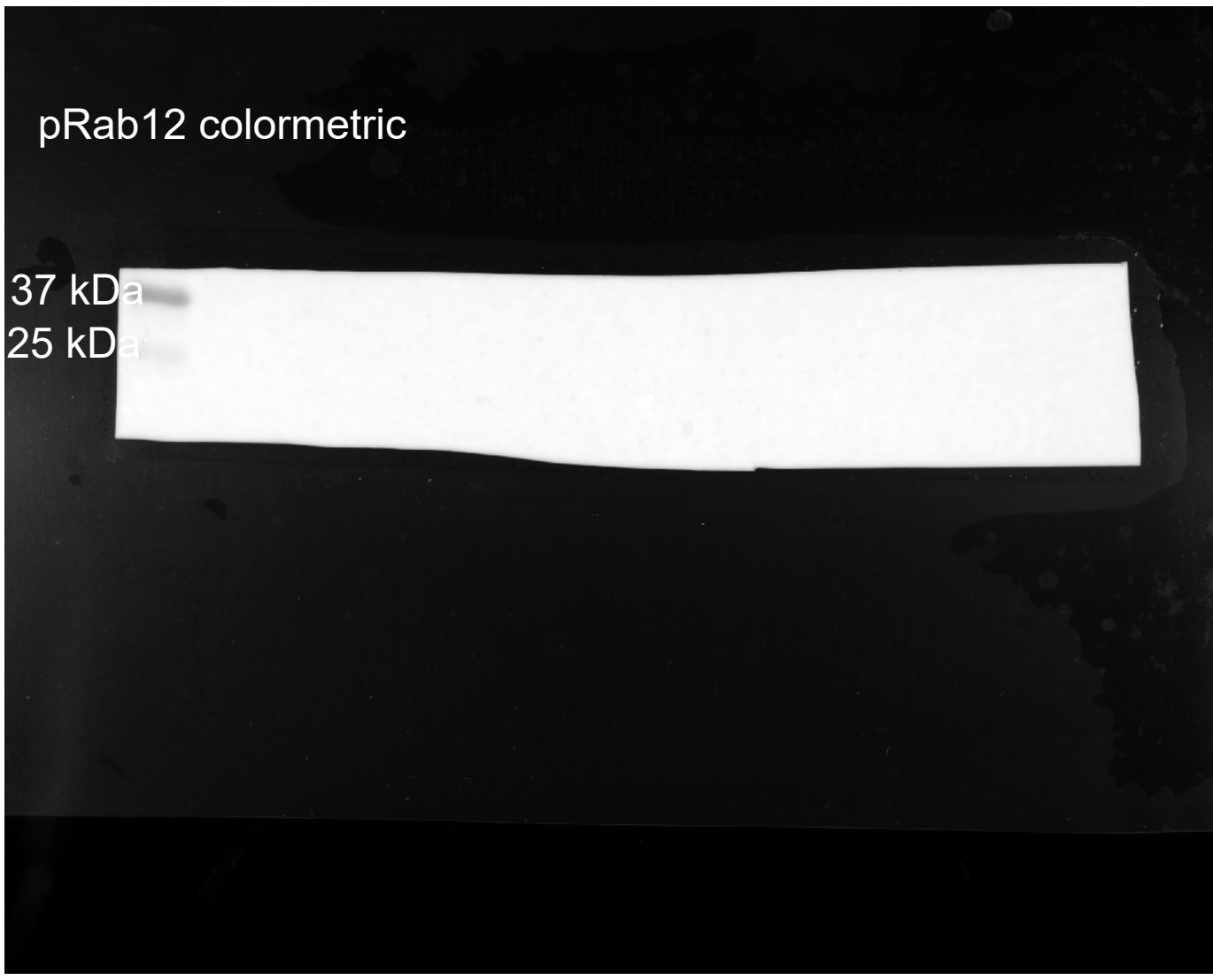
pRab12 colormetric
37 kDa
25 kDa

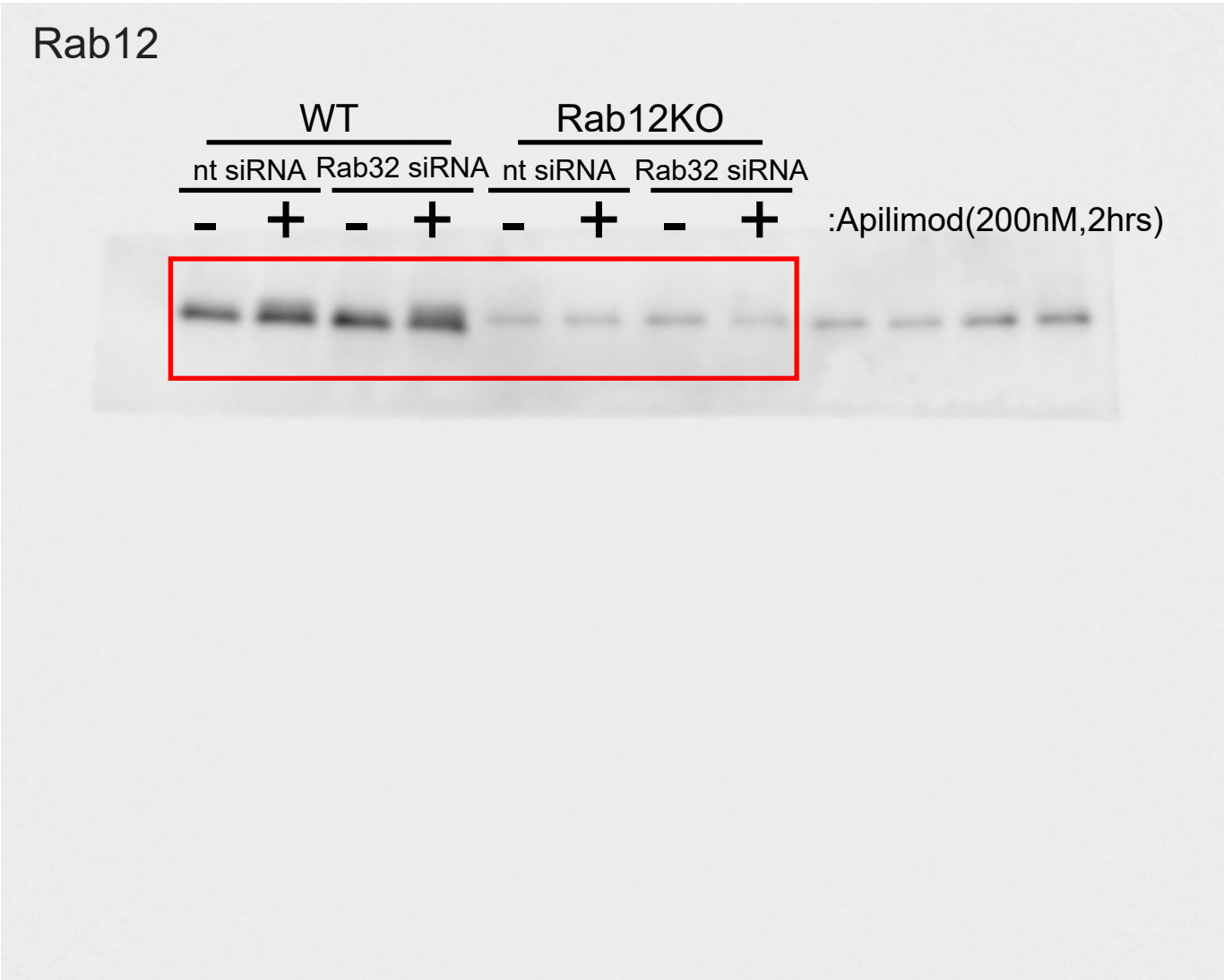
Rab12
WT
Rab12KO
nt siRNA
Rab32 siRNA
nt siRNA
Rab32 siRNA
- + - + - + - +
:Apilimod(200nM,2hrs)

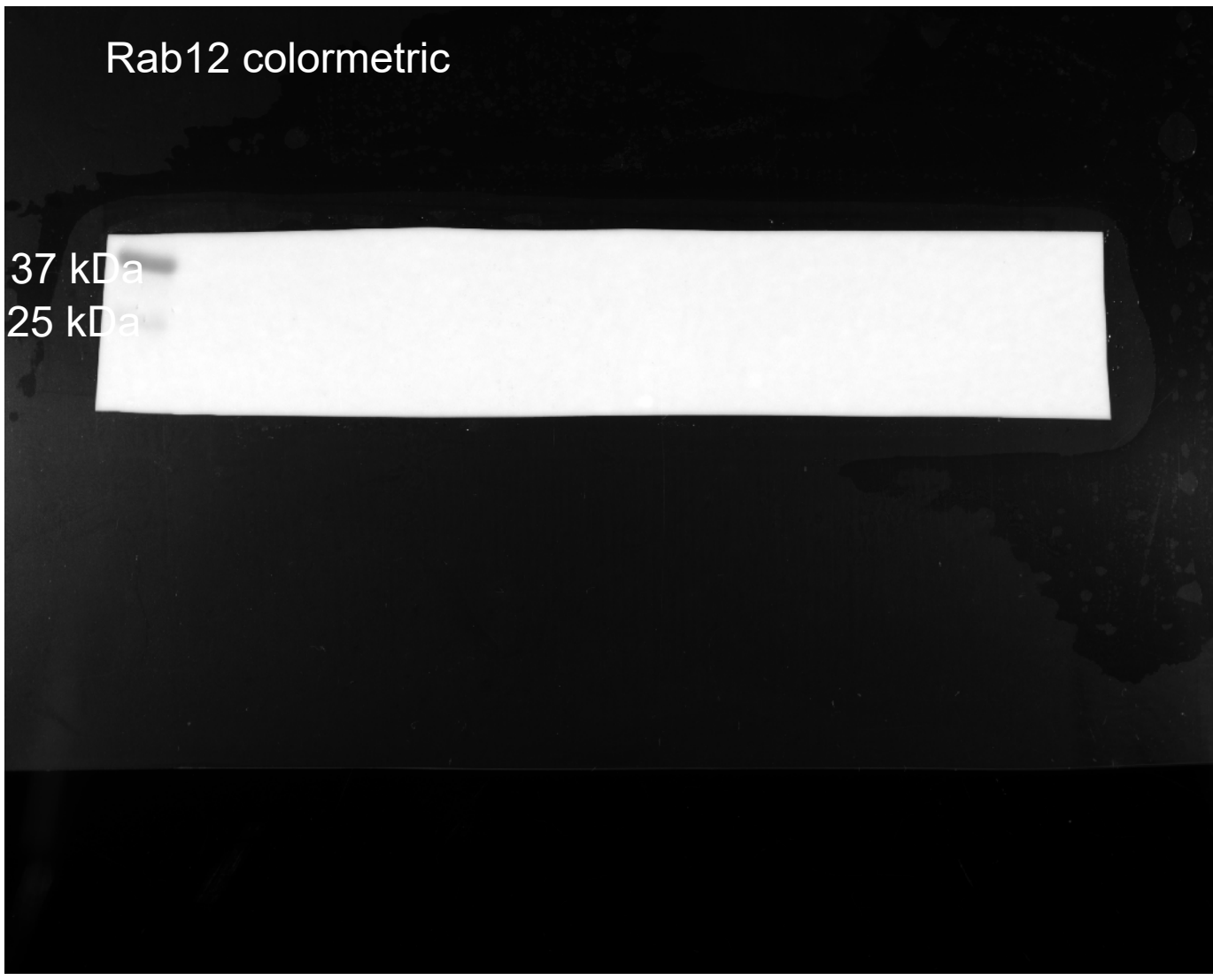
Rab12 colormetric
37 kDa
25 kDa

5C.

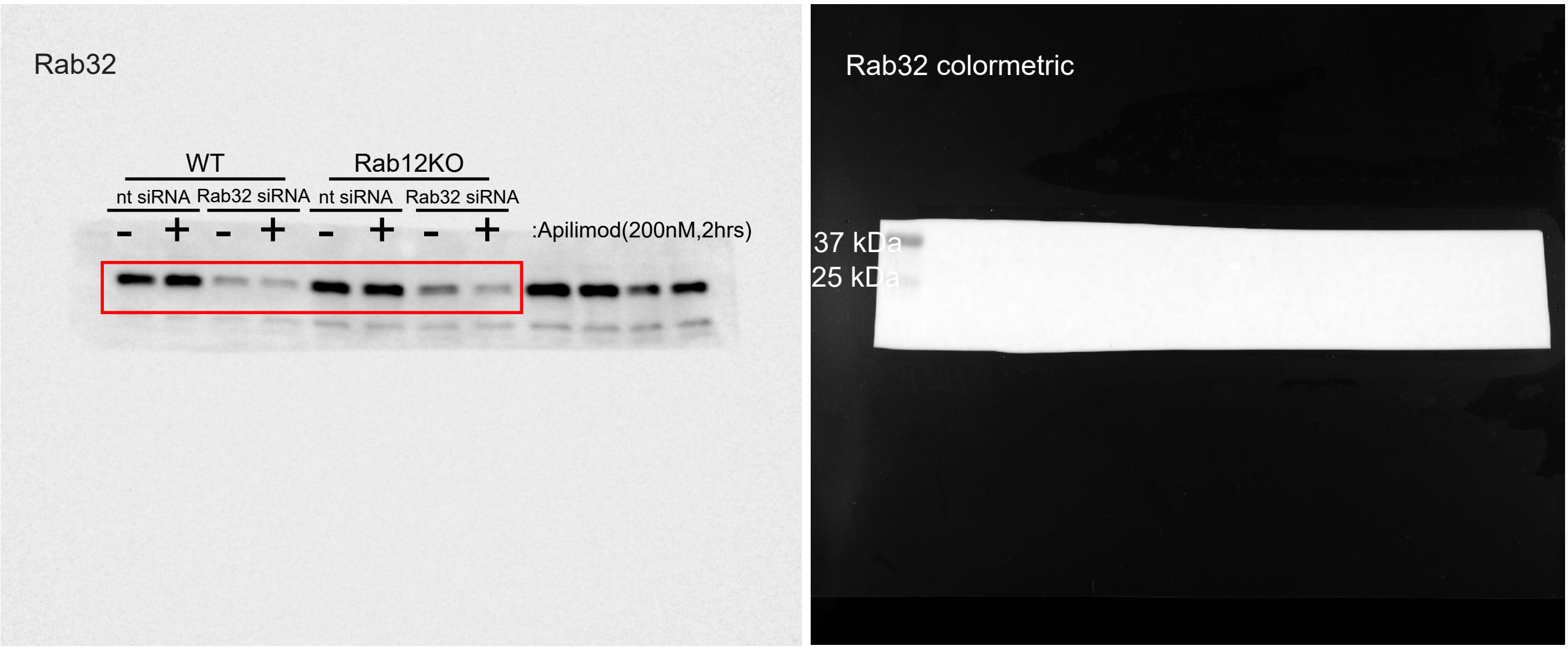
Rab32
WT
Rab12KO
nt siRNA
Rab32 siRNA
nt siRNA
Rab32 siRNA
- + - + - + - +
:Apilimod(200nM,2hrs)
Rab32 colormetric
37 kDa
25 kDa

5C.

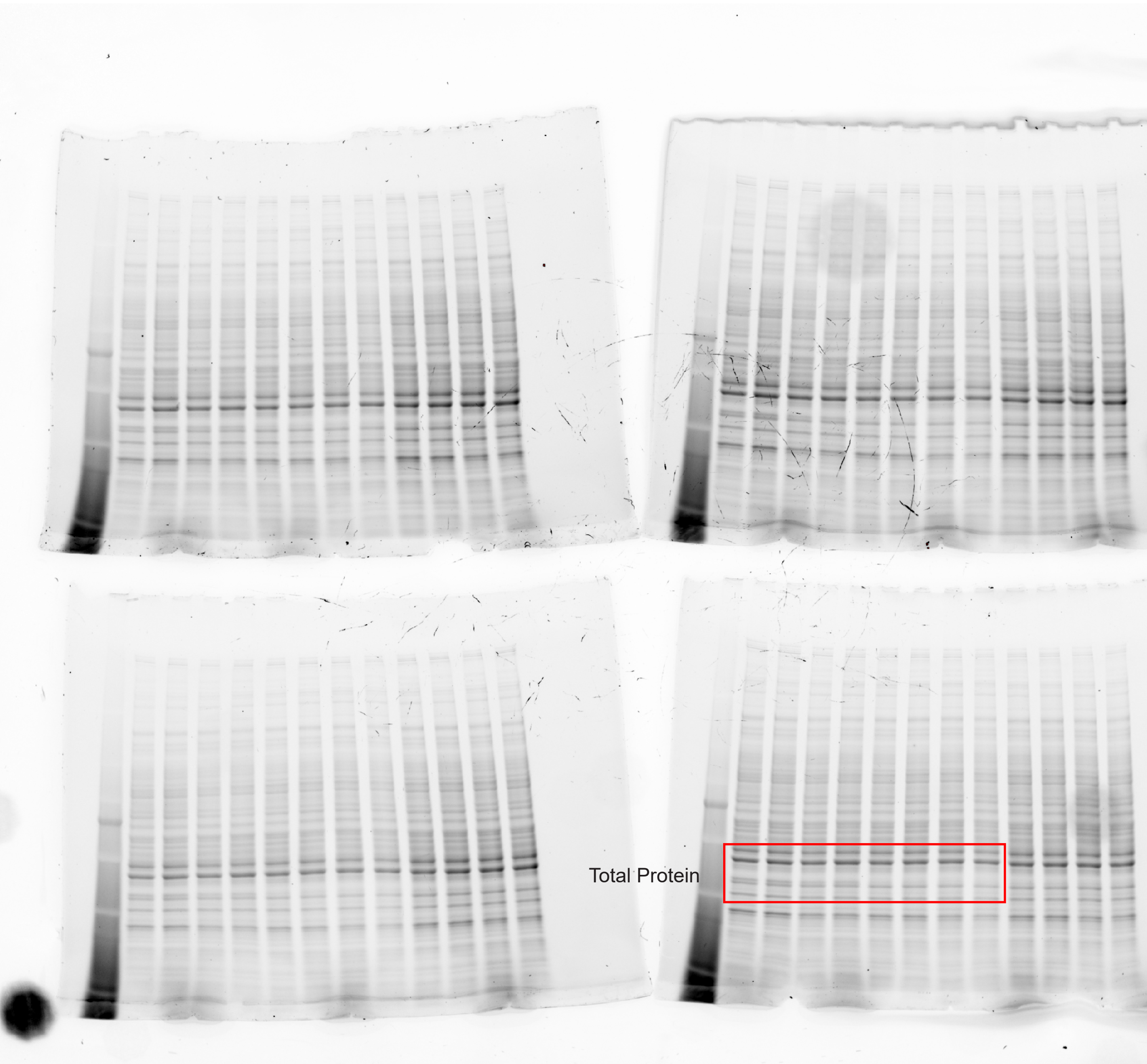

Supplement: Supplementary file 6 [file jcb_202511137_sourcedataf5.pdf]
