# Supplementary material for: ClC-7 links PIKfyve inhibition to Rab-dependent LRRK2 activity at lysosomes
Source: J Cell Biol. 2026 Oct 20;225(11):e202511137. doi: 10.1083/jcb.202511137 (PMC13682606; doi:10.1083/jcb.202511137)
Supplement: Supplementary file 7 [file jcb_202511137_sourcedatafs1.pdf]

S1A.

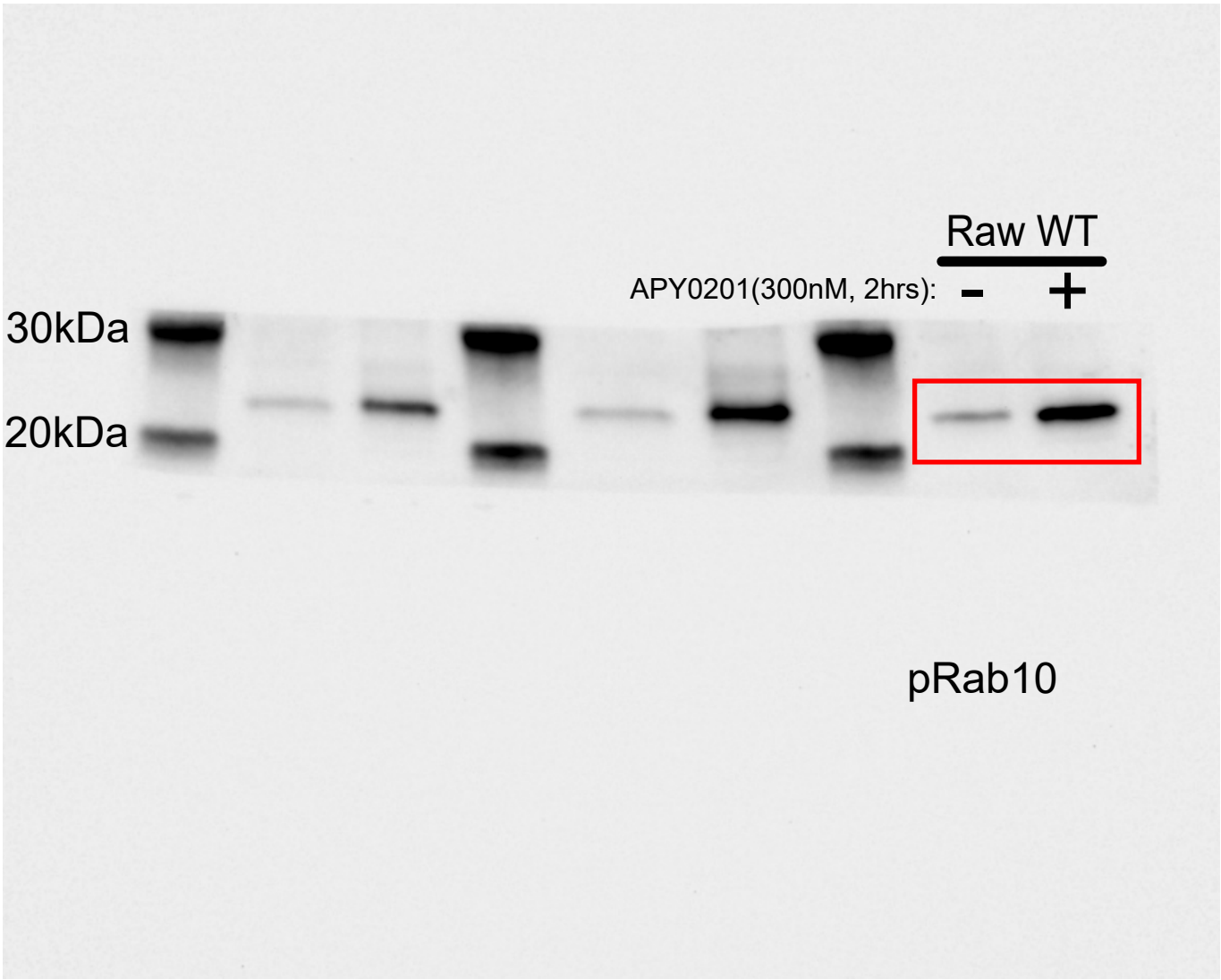
Raw WT
APY0201(300nM, 2hrs): - +
30kDa
20kDa
pRab10

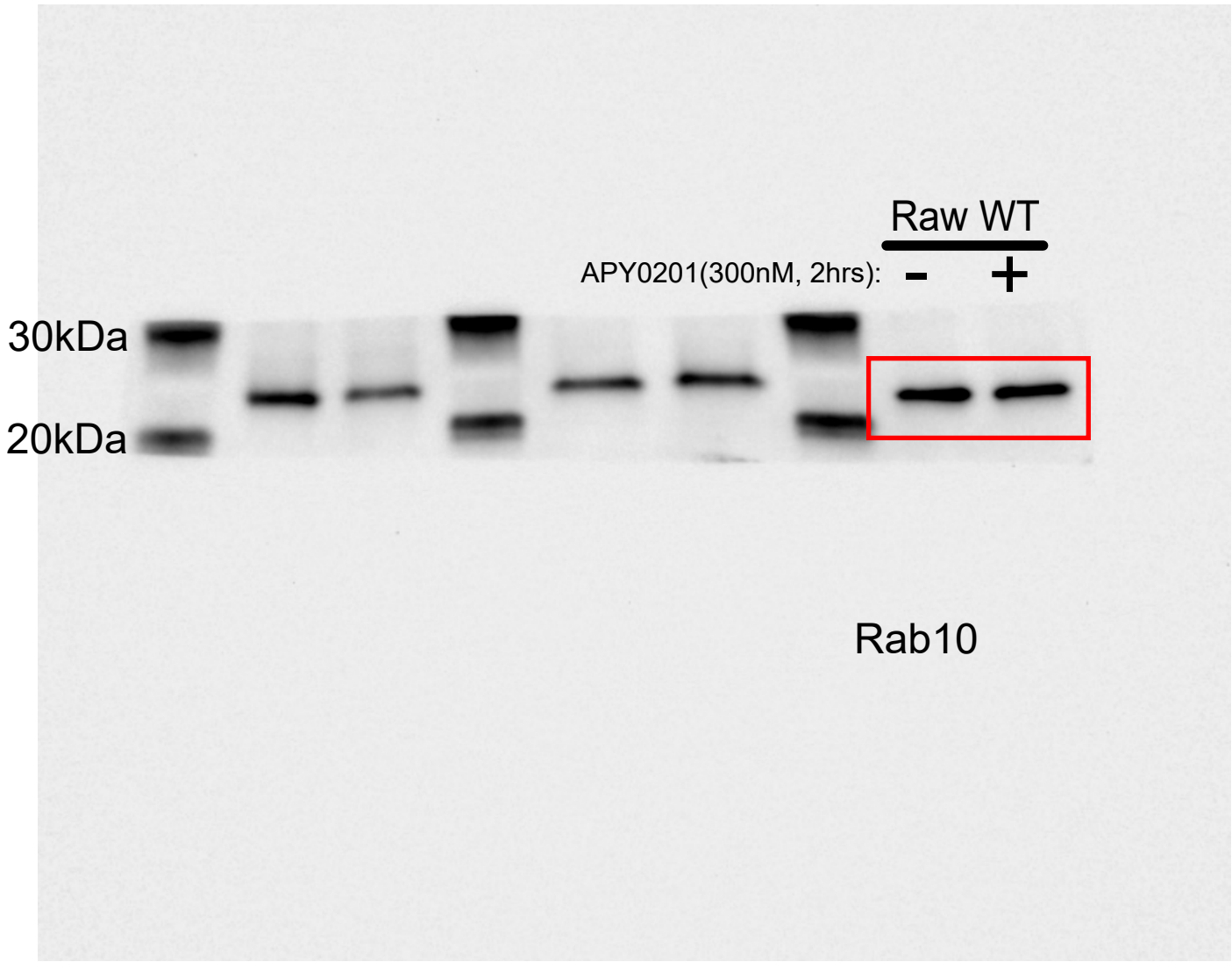
Raw WT
APY0201(300nM, 2hrs): - +
30kDa
20kDa
Rab10

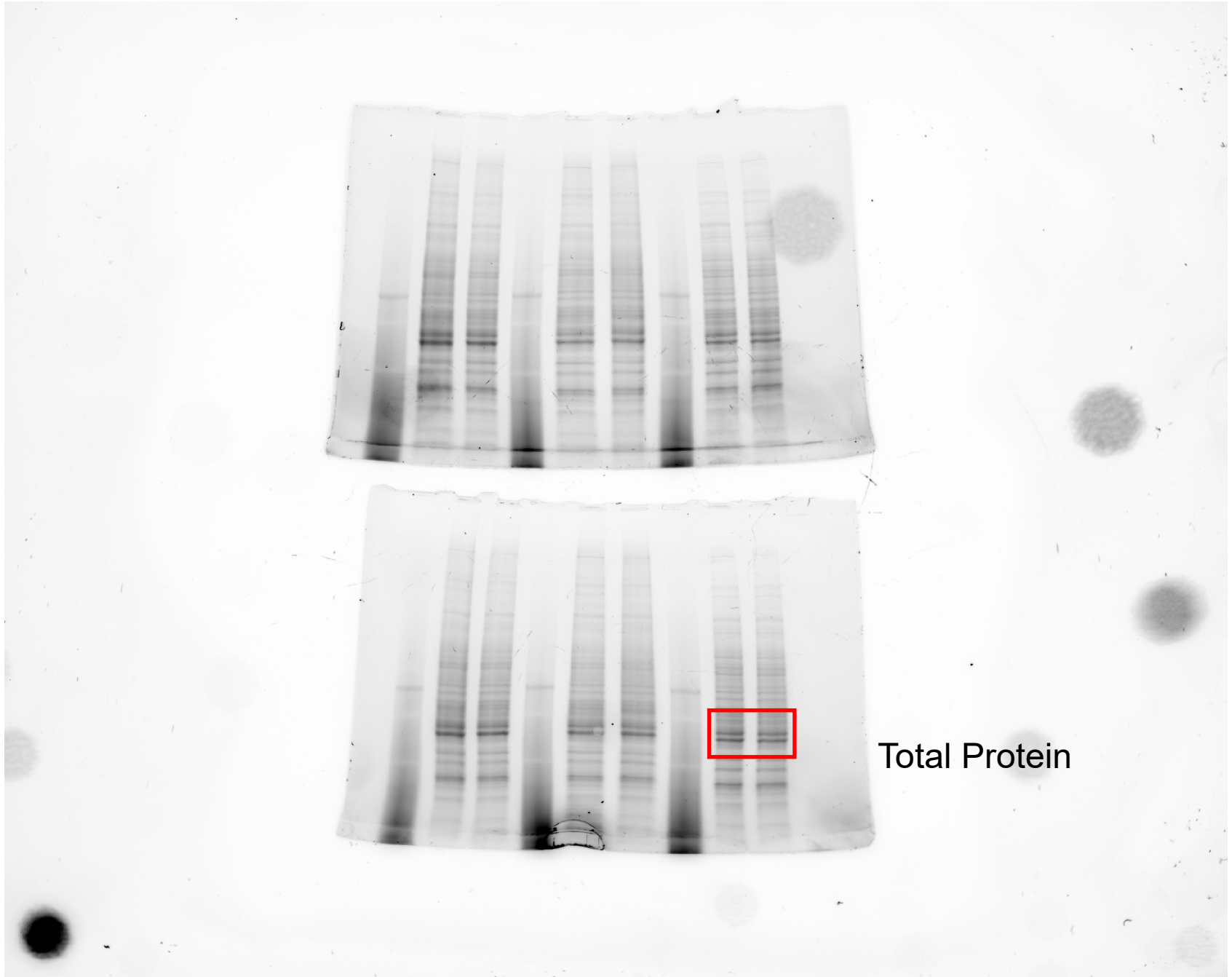
Total Protein

S1C.

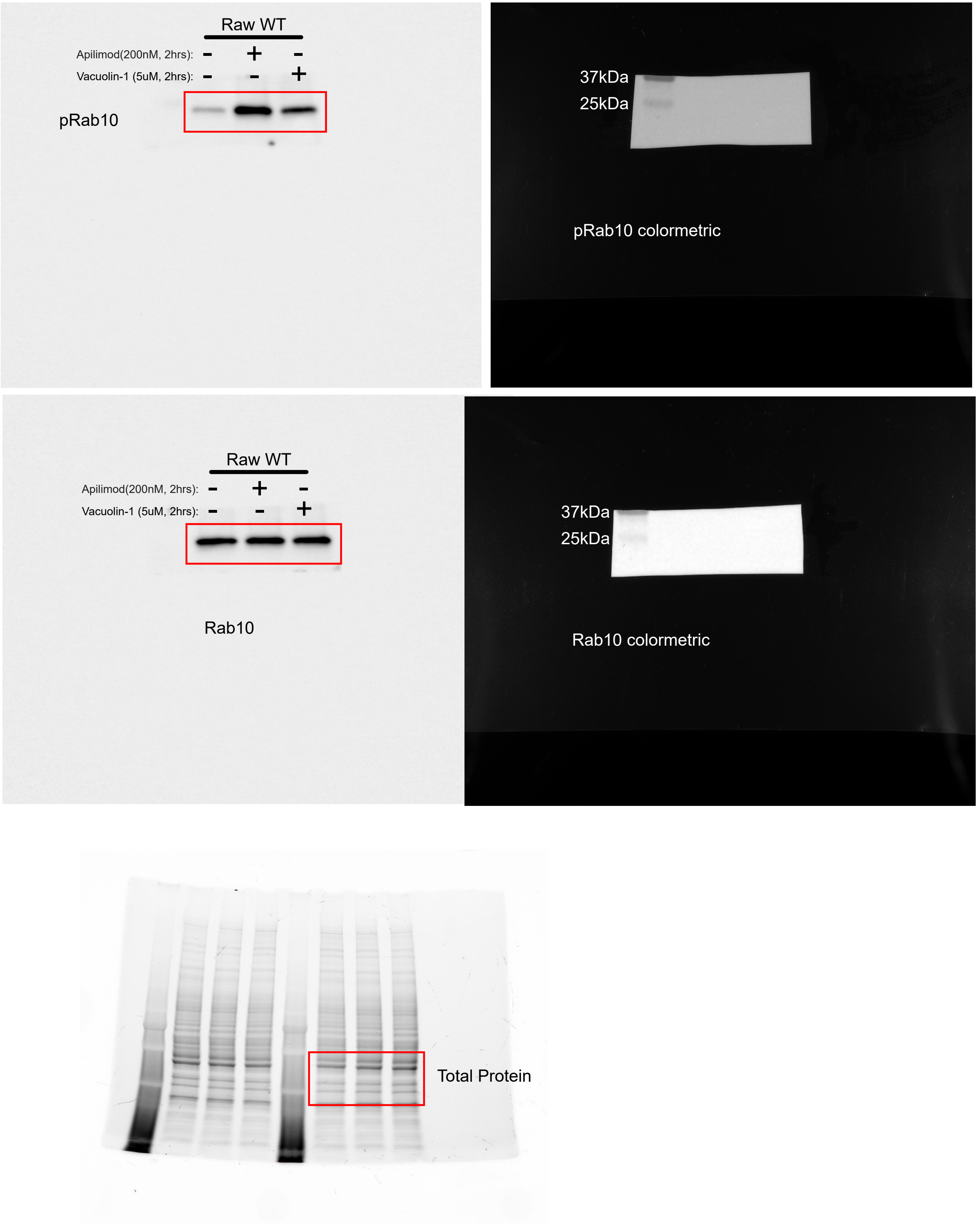
Raw WT
Apilimod(200nM, 2hrs): - + -
Vacuolin-1 (5uM, 2hrs): - - +
pRab10
37kDa
25kDa
pRab10 colormetric
Raw WT
Apilimod(200nM, 2hrs): - + -
Vacuolin-1 (5uM, 2hrs): - - +
Rab10
37kDa
25kDa
Rab10 colormetric
Total Protein

S1E.

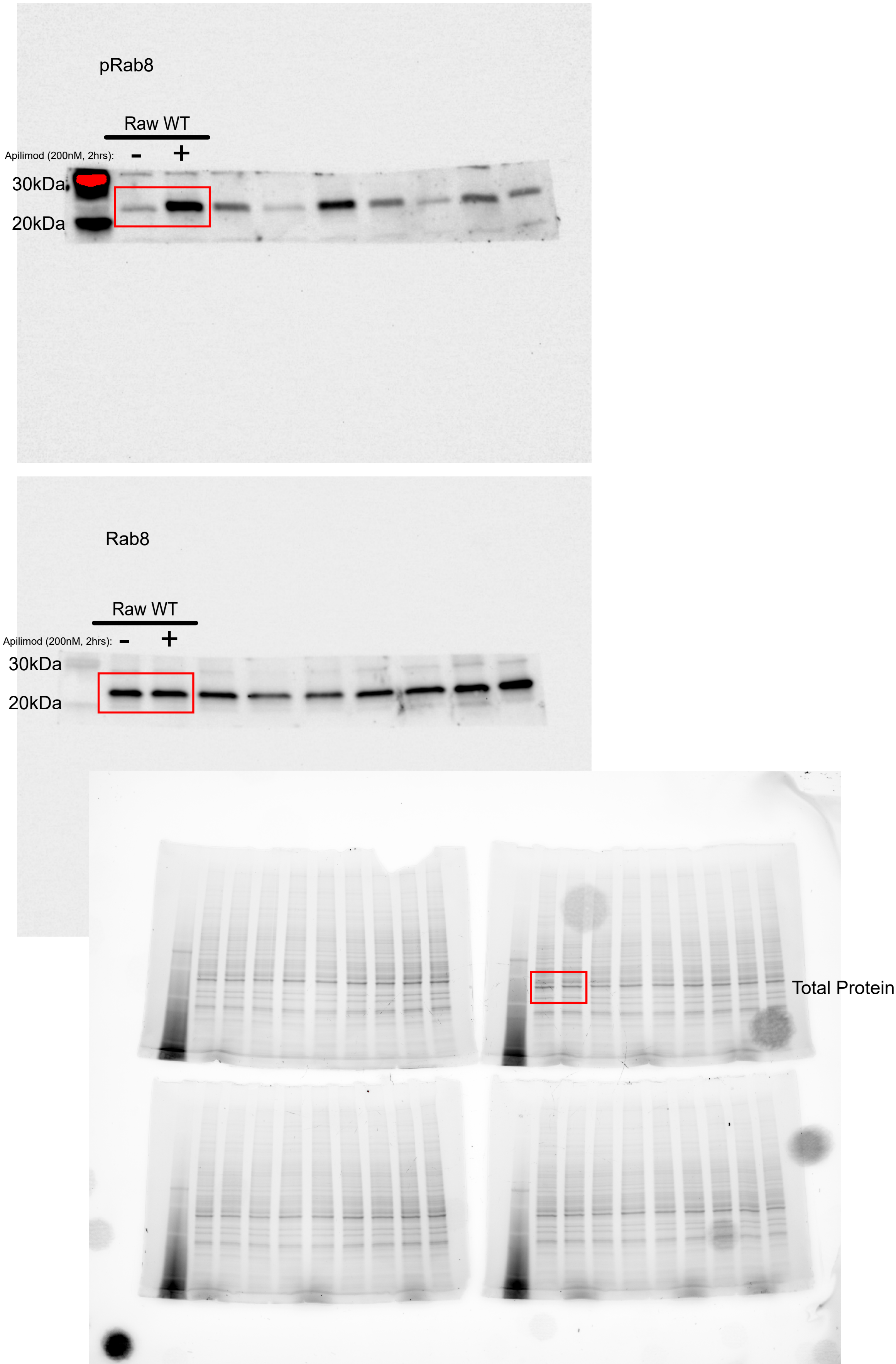
pRab8
Raw WT
Apilimod (200nM, 2hrs): - +
30kDa
20kDa
Rab8
Raw WT
Apilimod (200nM, 2hrs): - +
30kDa
20kDa
Total Protein

S1G.

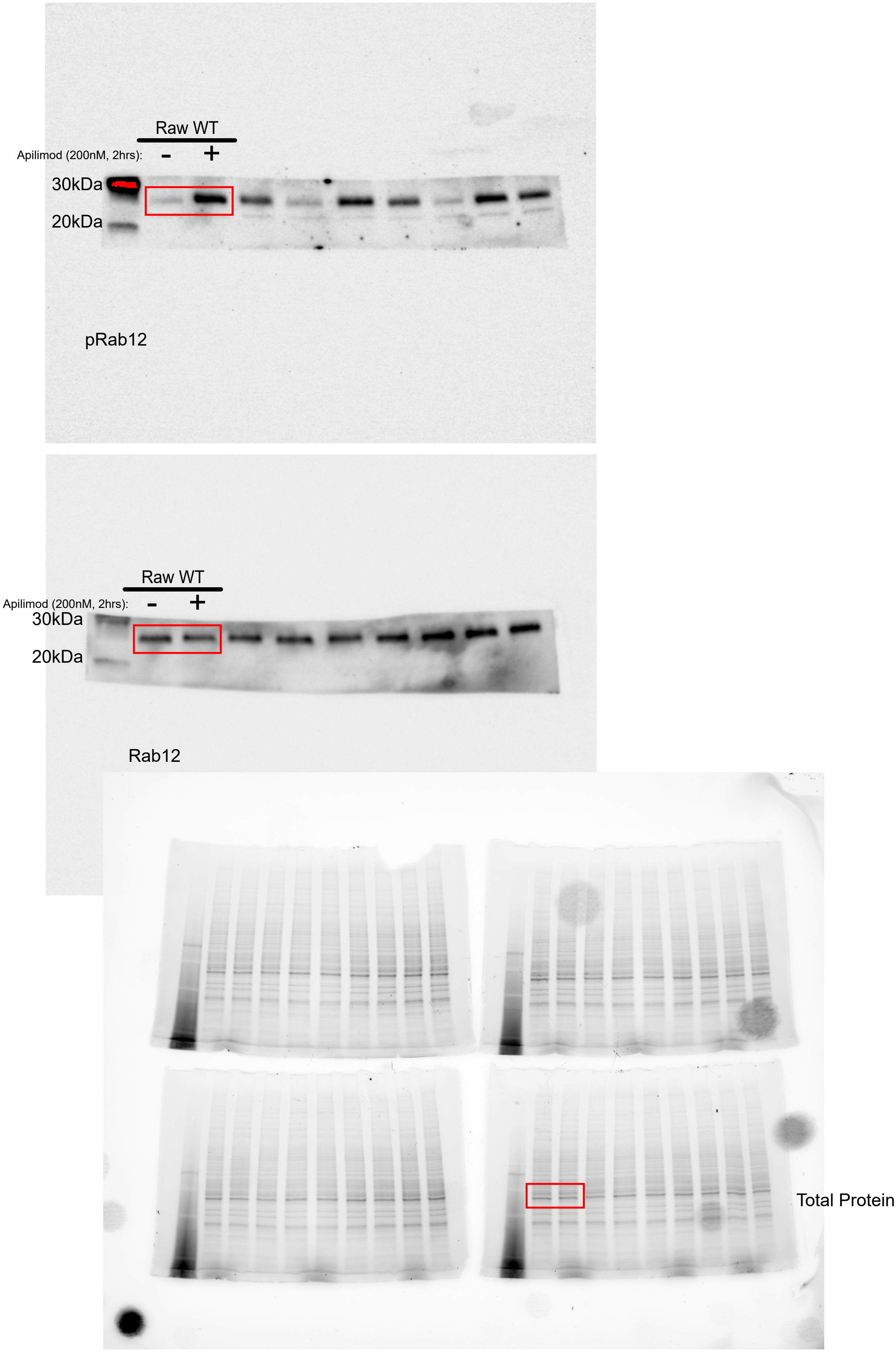
Raw WT
Apilimod (200nM, 2hrs): - +
30kDa
20kDa
pRab12
Raw WT
Apilimod (200nM, 2hrs): - +
30kDa
20kDa
Rab12
Total Protein

S1I.

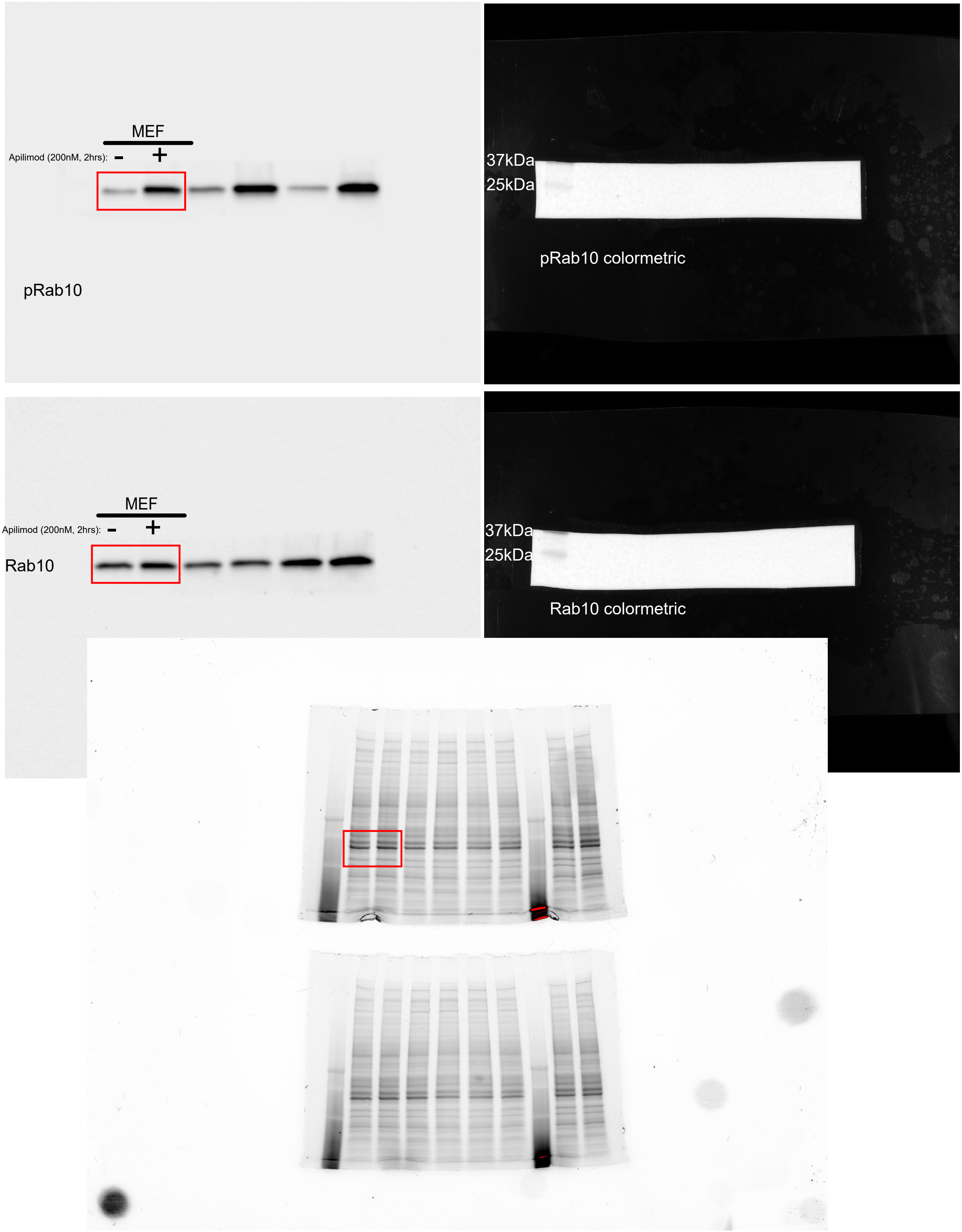
MEF
Apilimod (200nM, 2hrs): - +
pRab10
37kDa
25kDa
pRab10 colormetric
MEF
Apilimod (200nM, 2hrs): - +
Rab10
37kDa
25kDa
Rab10 colormetric

S1K.

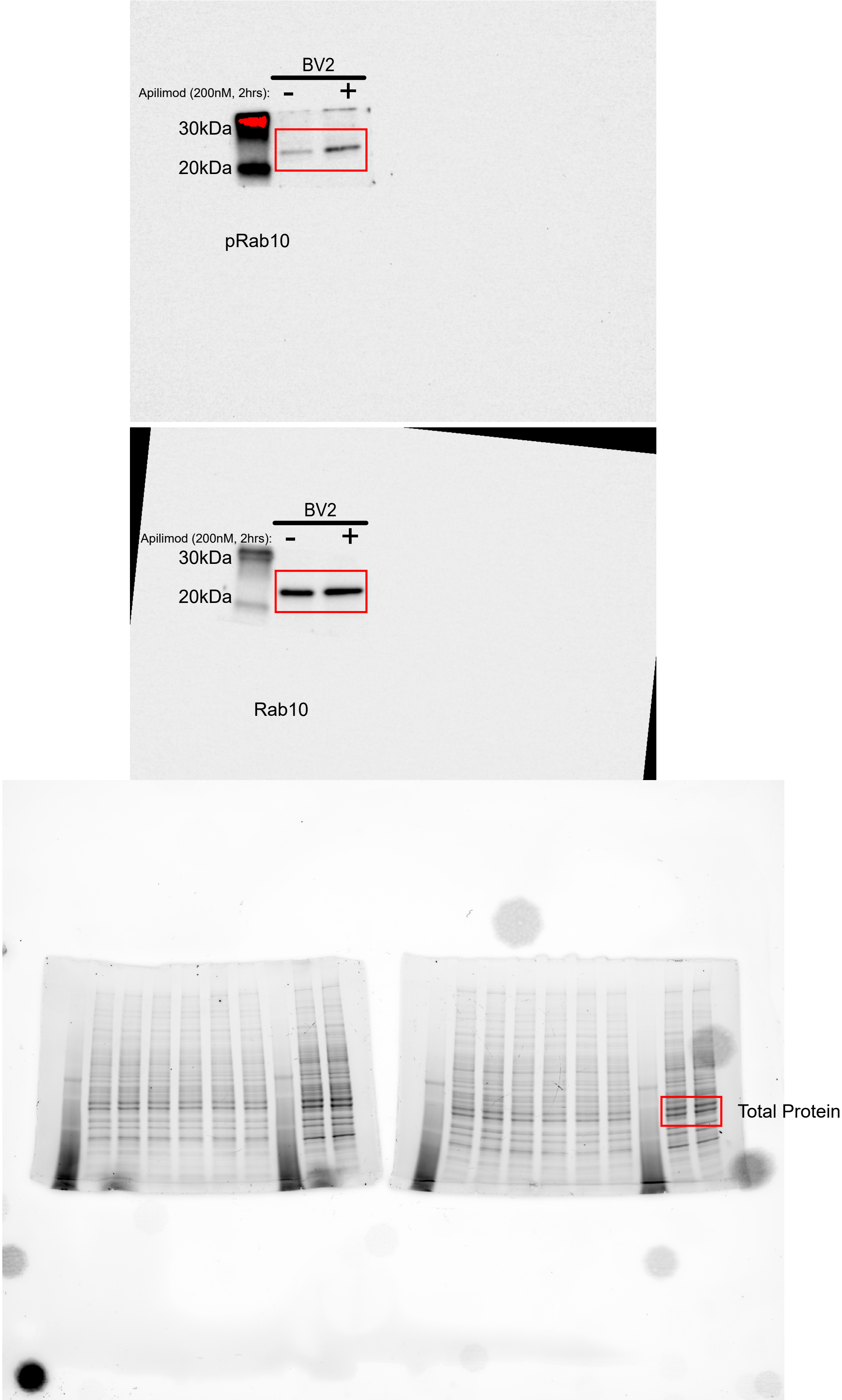
BV2
Apilimod (200nM, 2hrs): - +
30kDa
20kDa
pRab10
BV2
Apilimod (200nM, 2hrs): - +
30kDa
20kDa
Rab10
Total Protein
