# Supplementary material for: ClC-7 links PIKfyve inhibition to Rab-dependent LRRK2 activity at lysosomes
Source: J Cell Biol. 2026 Oct 20;225(11):e202511137. doi: 10.1083/jcb.202511137 (PMC13682606; doi:10.1083/jcb.202511137)
Supplement: Supplementary file 8 [file jcb_202511137_sourcedatafs2.pdf]

S2C

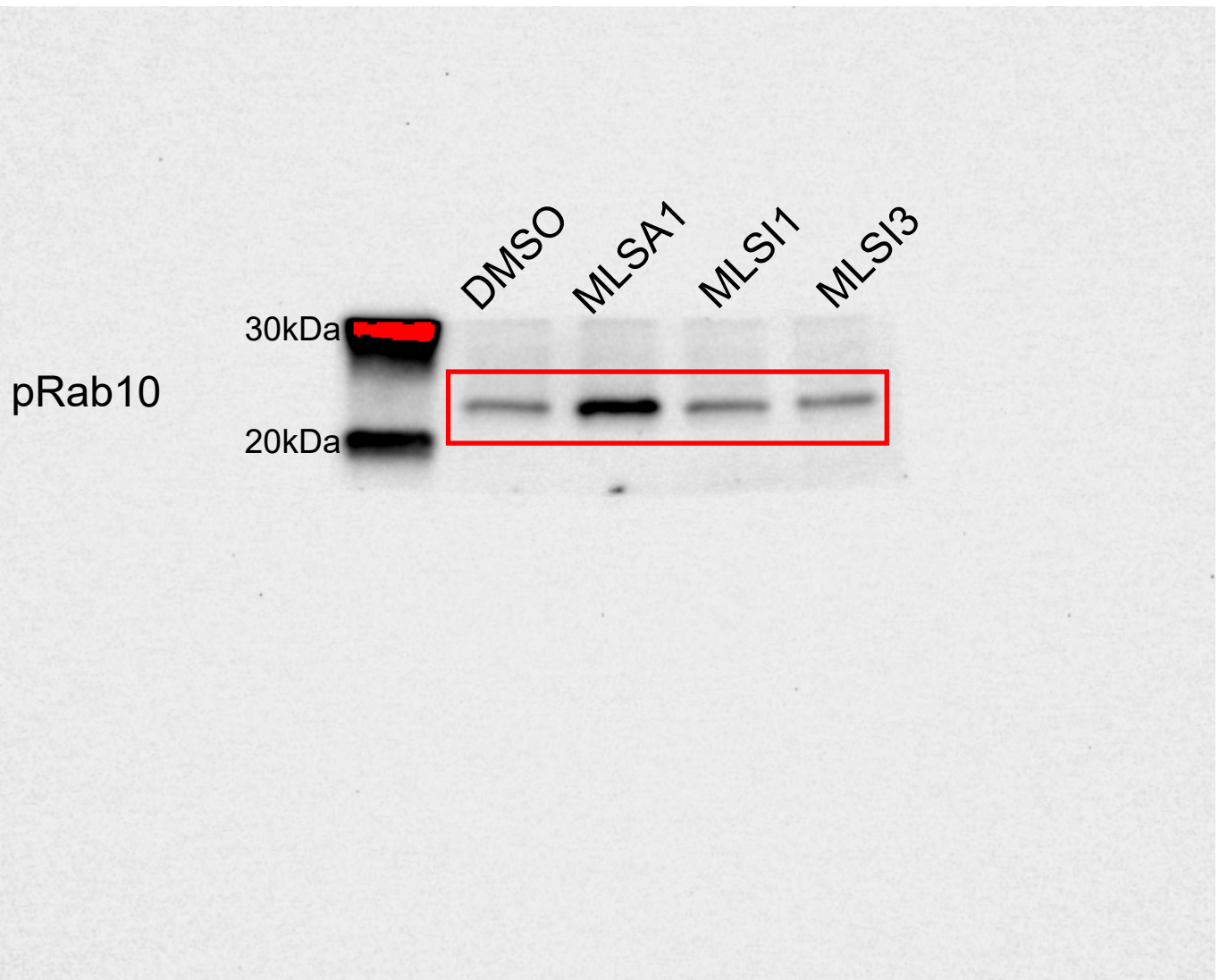
DMSO
MLSA1
MLSI1
MLSI3
30kDa
pRab10
20kDa

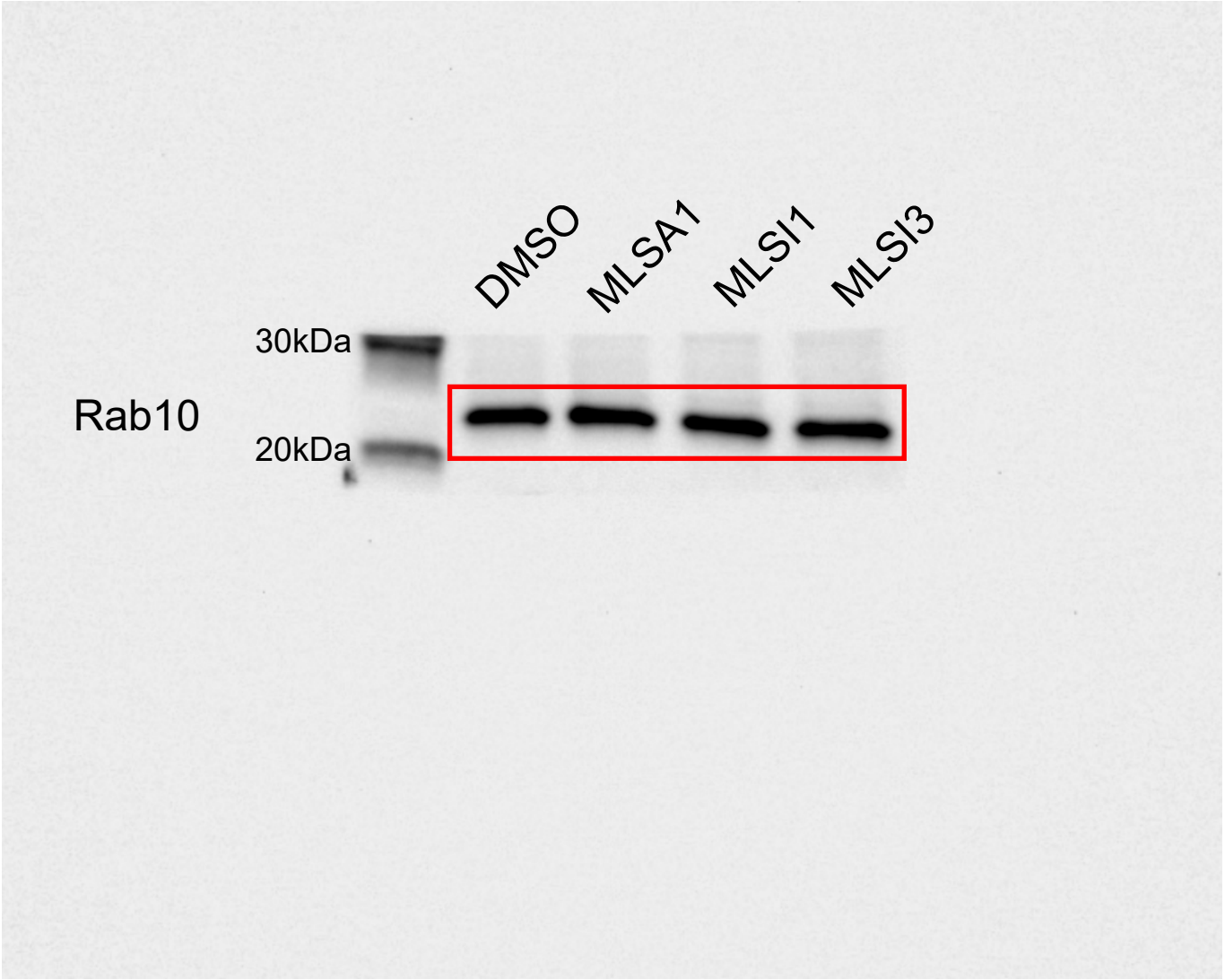
DMSO
MLSA1
MLSI1
MLSI3
30kDa
Rab10
20kDa

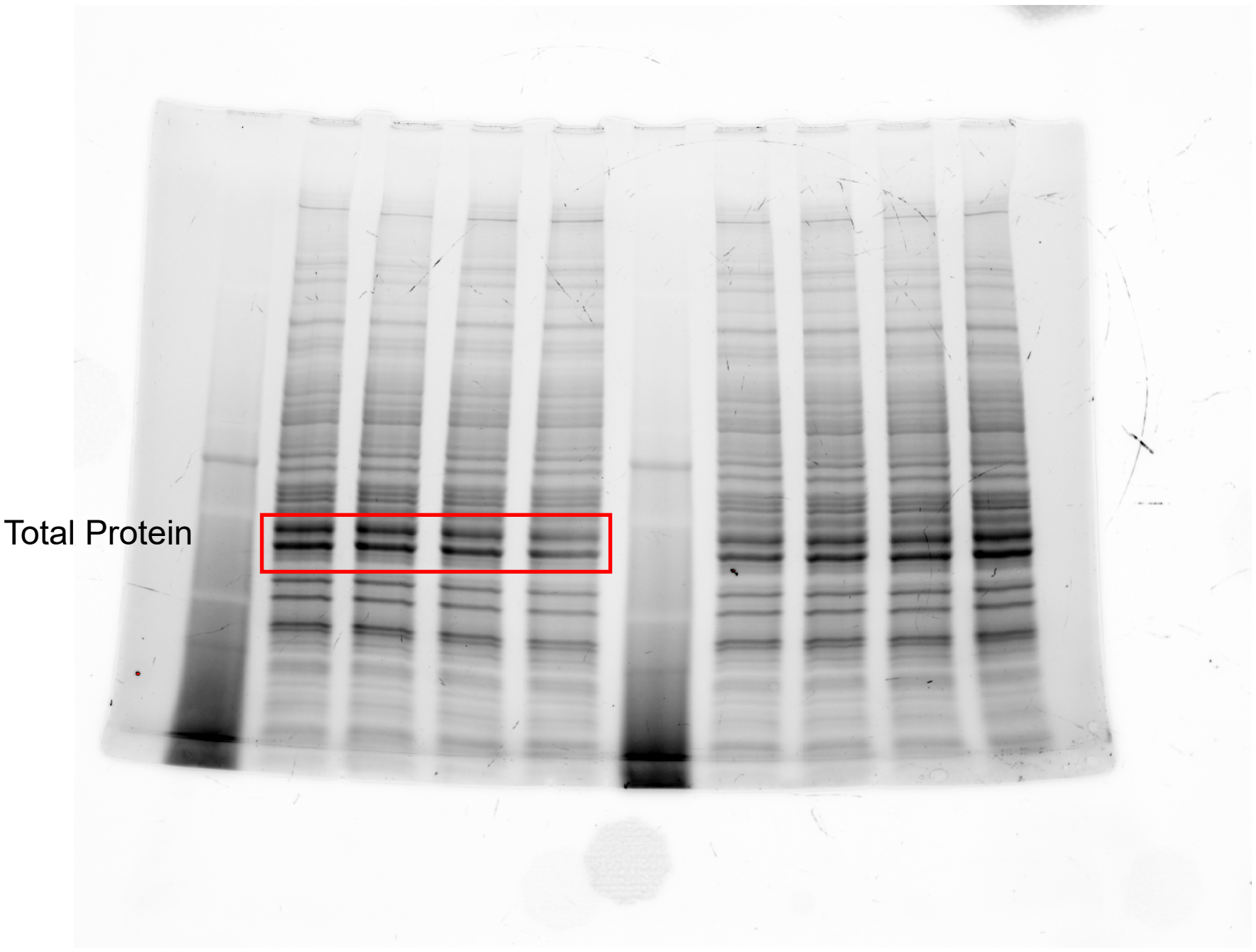
Total Protein

S2F

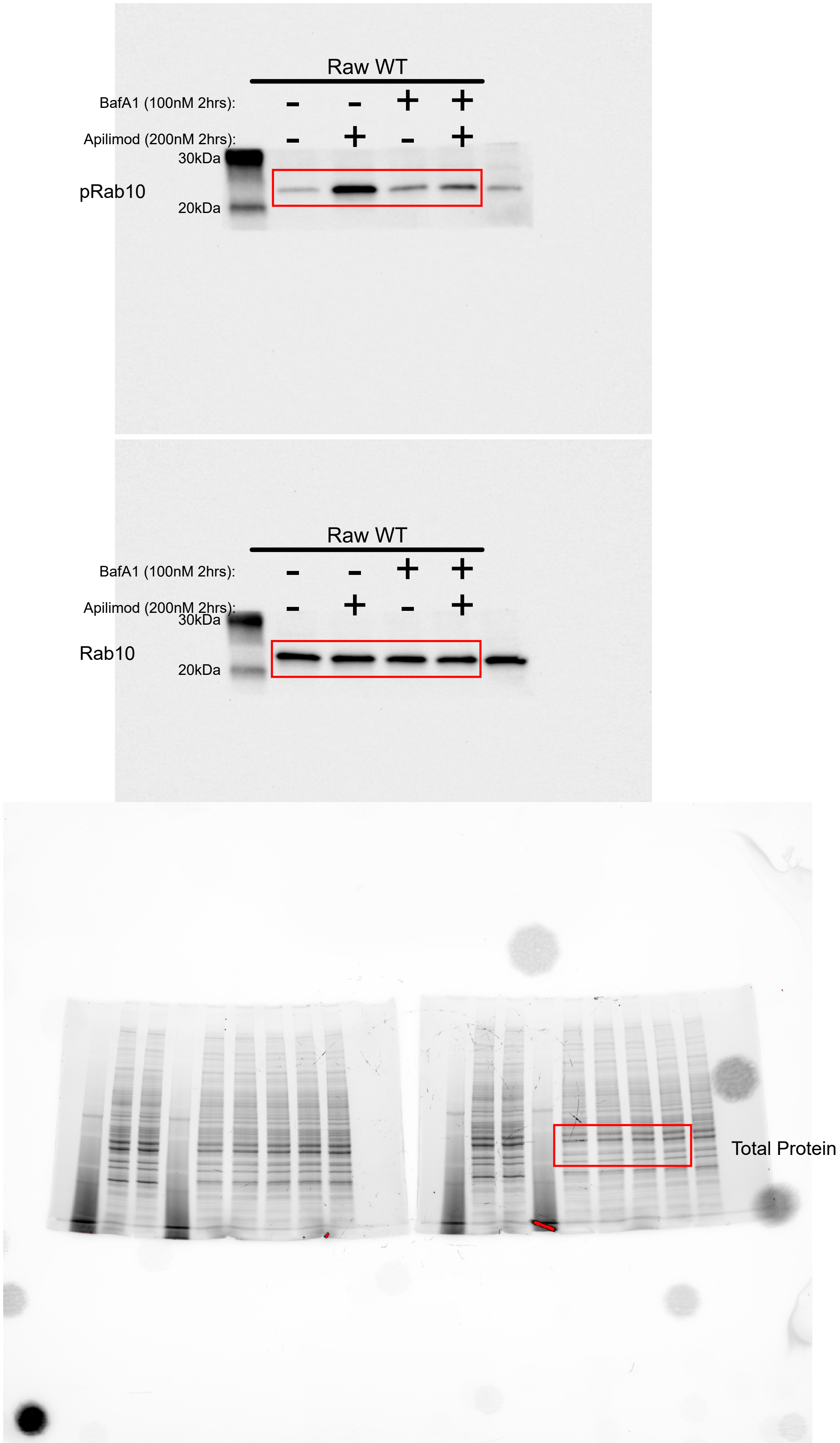
Raw WT
BafA1 (100nM 2hrs): - - + +
Apilimod (200nM 2hrs): - + - +
30kDa
pRab10
20kDa
Raw WT
BafA1 (100nM 2hrs): - - + +
Apilimod (200nM 2hrs): - + - +
30kDa
Rab10
20kDa
Total Protein

S2H

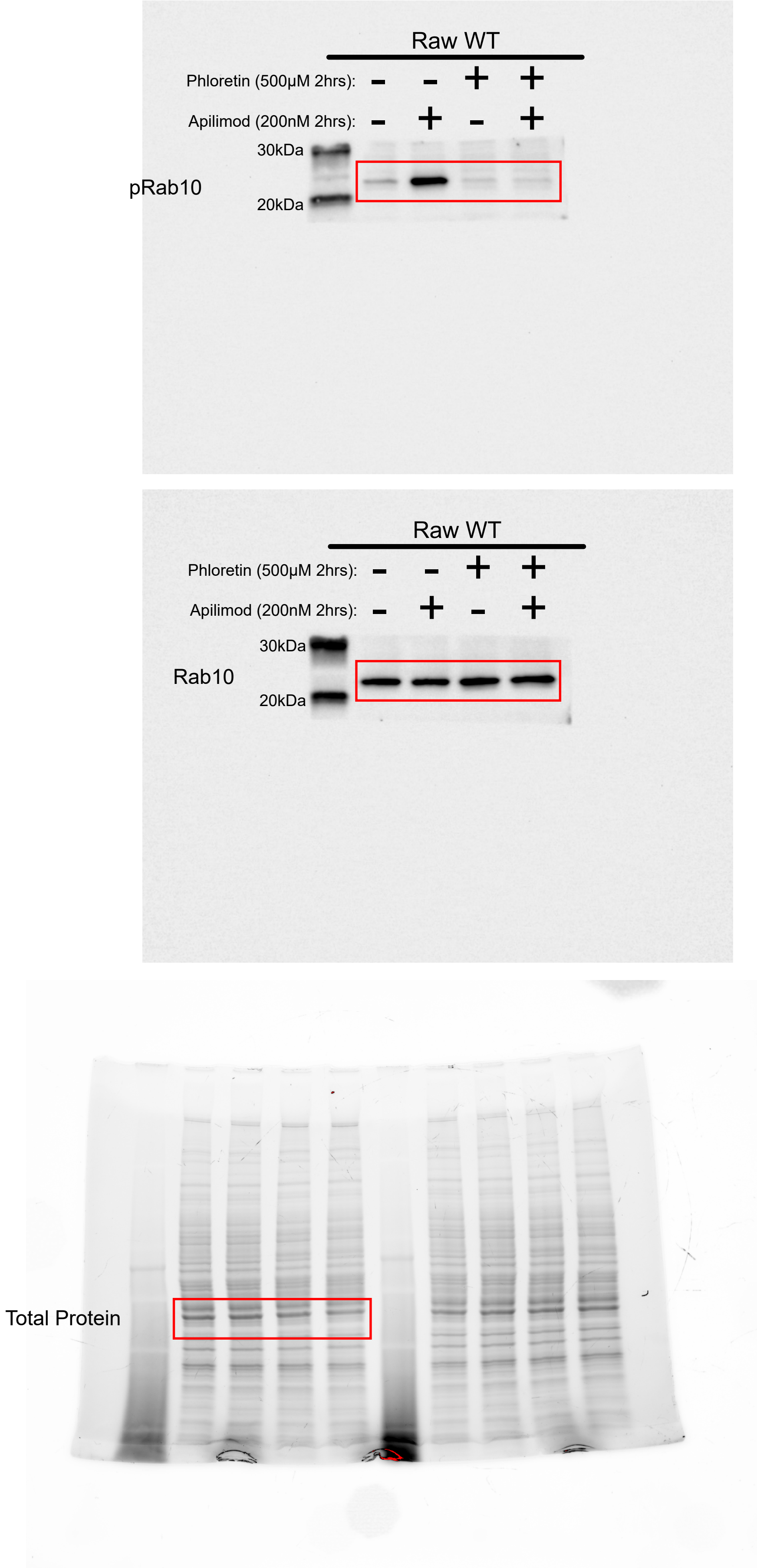

Raw WT
Phloretin (500μM 2hrs): – – + +
Apilimod (200nM 2hrs): – + – +
30kDa
pRab10
20kDa
Raw WT
Phloretin (500μM 2hrs): – – + +
Apilimod (200nM 2hrs): – + – +
30kDa
Rab10
20kDa
Total Protein
