# Supplementary material for: ClC-7 links PIKfyve inhibition to Rab-dependent LRRK2 activity at lysosomes
Source: J Cell Biol. 2026 Oct 20;225(11):e202511137. doi: 10.1083/jcb.202511137 (PMC13682606; doi:10.1083/jcb.202511137)
Supplement: Supplementary file 9 [file jcb_202511137_sourcedatafs3.pdf]

S3C

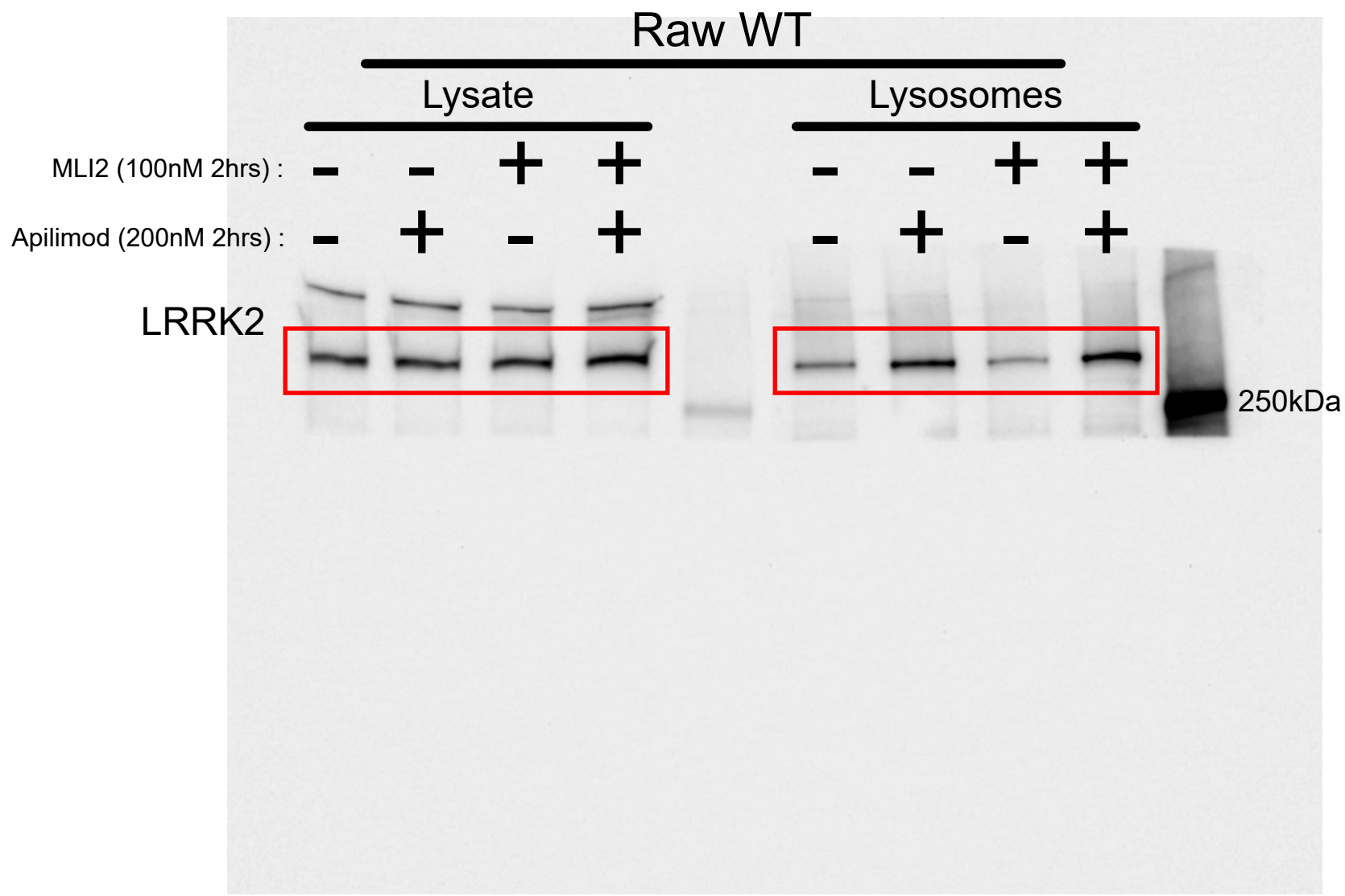
Raw WT
Lysate
Lysosomes
MLI2 (100nM 2hrs) :
Apilimod (200nM 2hrs) :
LRRK2
250kDa

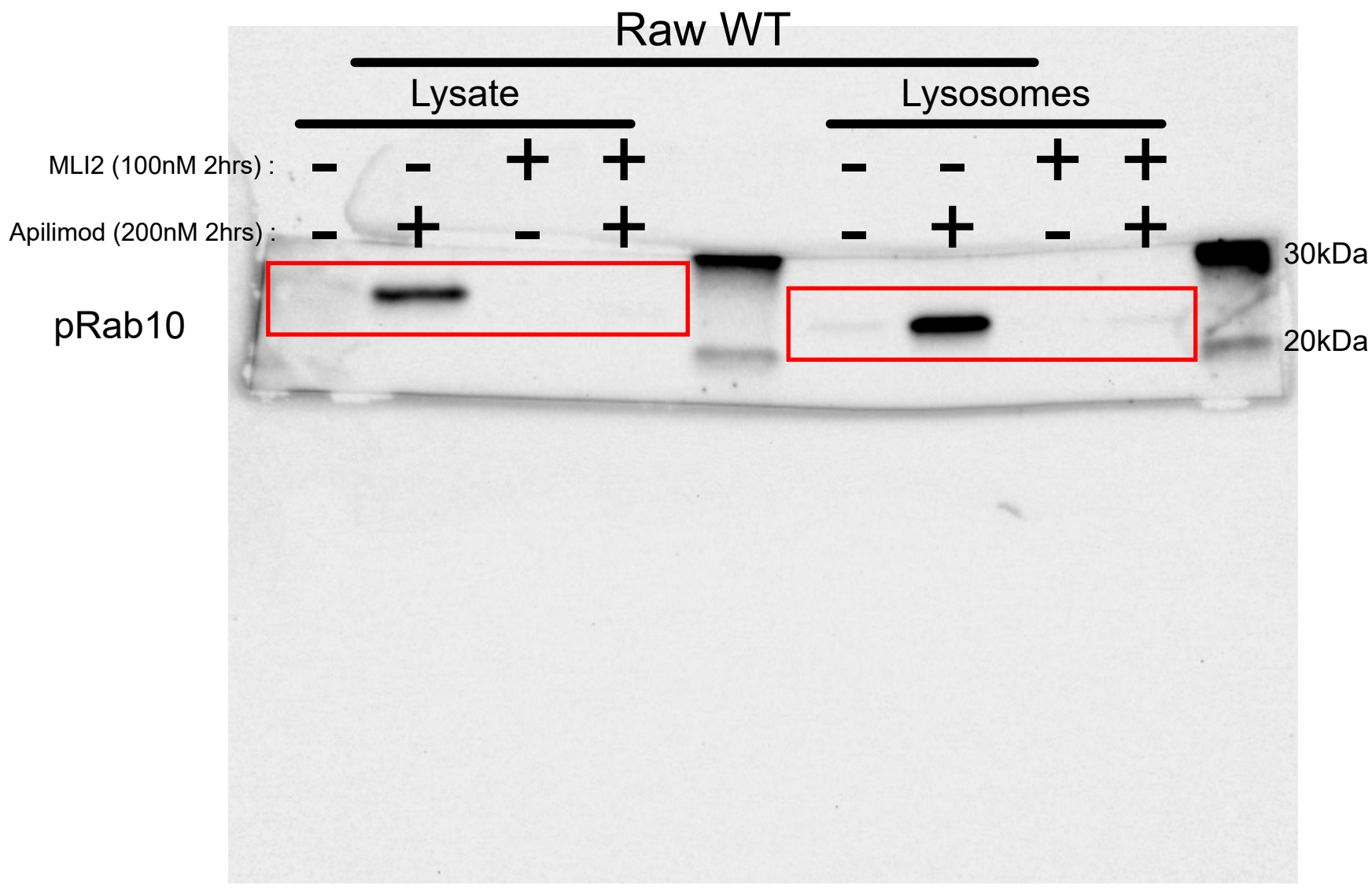
Raw WT
Lysate
Lysosomes
MLI2 (100nM 2hrs) :
Apilimod (200nM 2hrs) :
pRab10
30kDa
20kDa

S3C

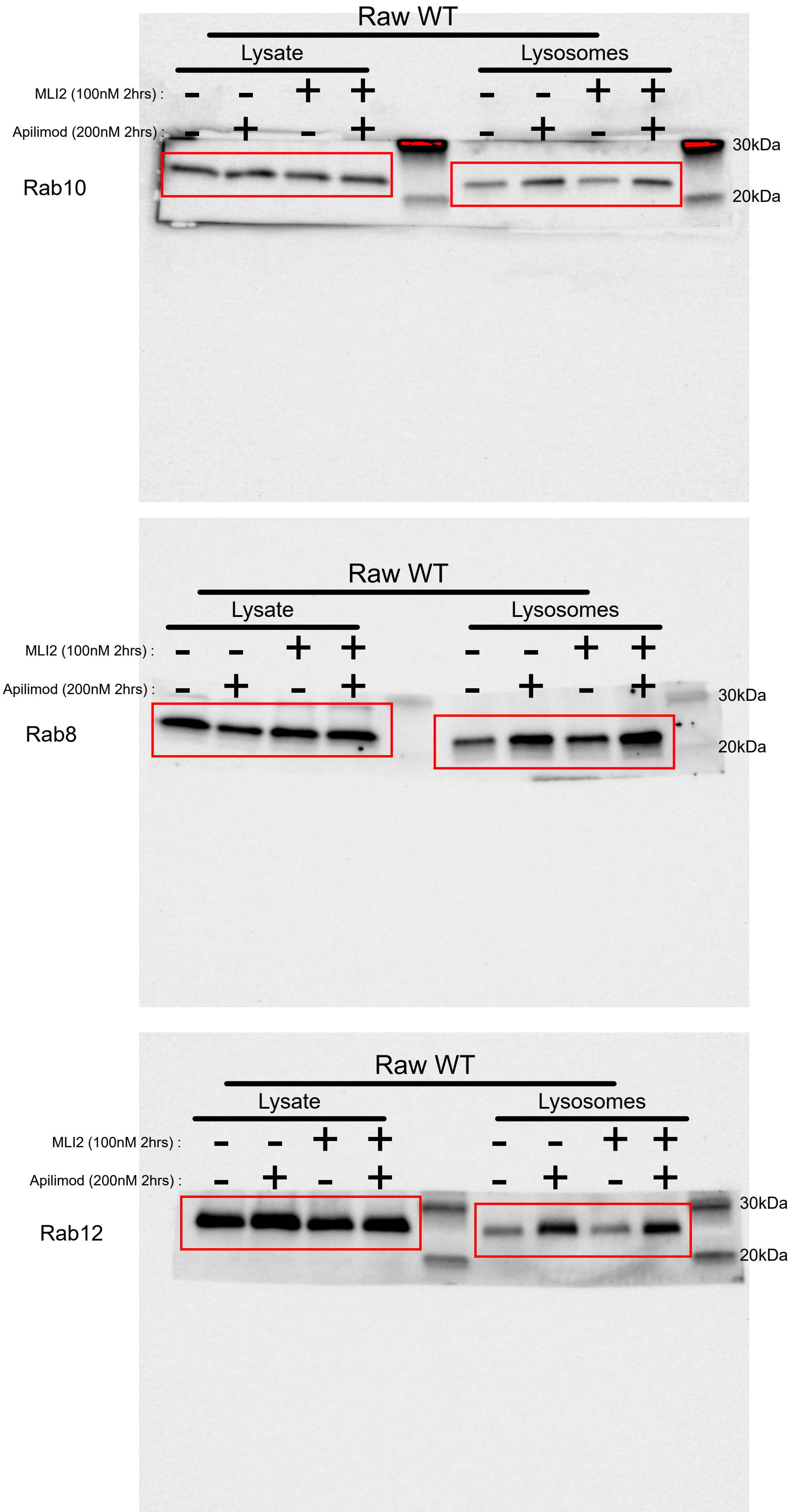

Raw WT
Lysate
Lysosomes
MLI2 (100nM 2hrs) :
Apilimod (200nM 2hrs) :
Rab10
30kDa
20kDa
Raw WT
Lysate
Lysosomes
MLI2 (100nM 2hrs) :
Apilimod (200nM 2hrs) :
Rab8
30kDa
20kDa
Raw WT
Lysate
Lysosomes
MLI2 (100nM 2hrs) :
Apilimod (200nM 2hrs) :
Rab12
30kDa
20kDa

S3C

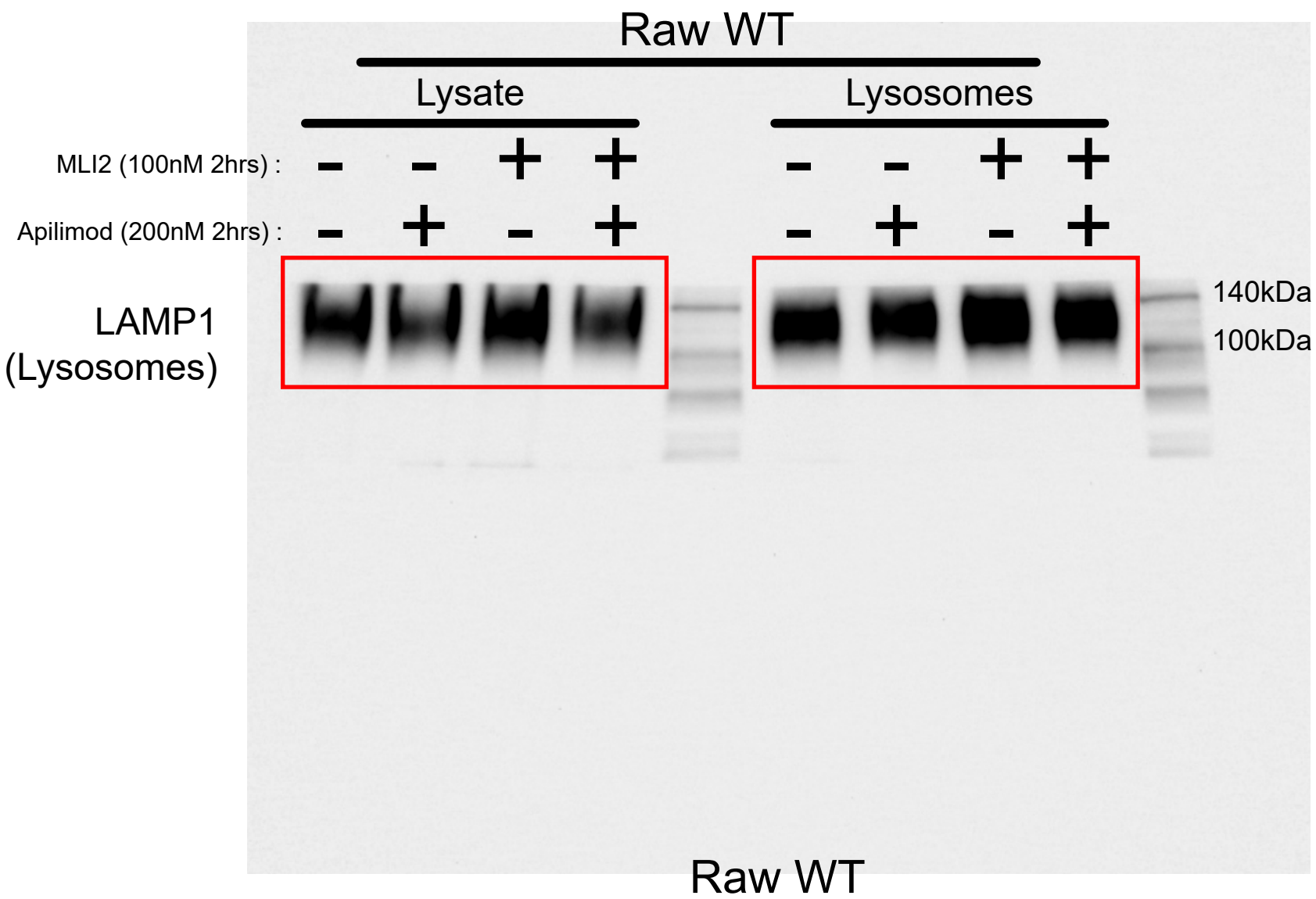

Raw WT
Lysate
Lysosomes
MLI2 (100nM 2hrs) :
Apilimod (200nM 2hrs) :
LAMP1
(Lysosomes)
140kDa
100kDa

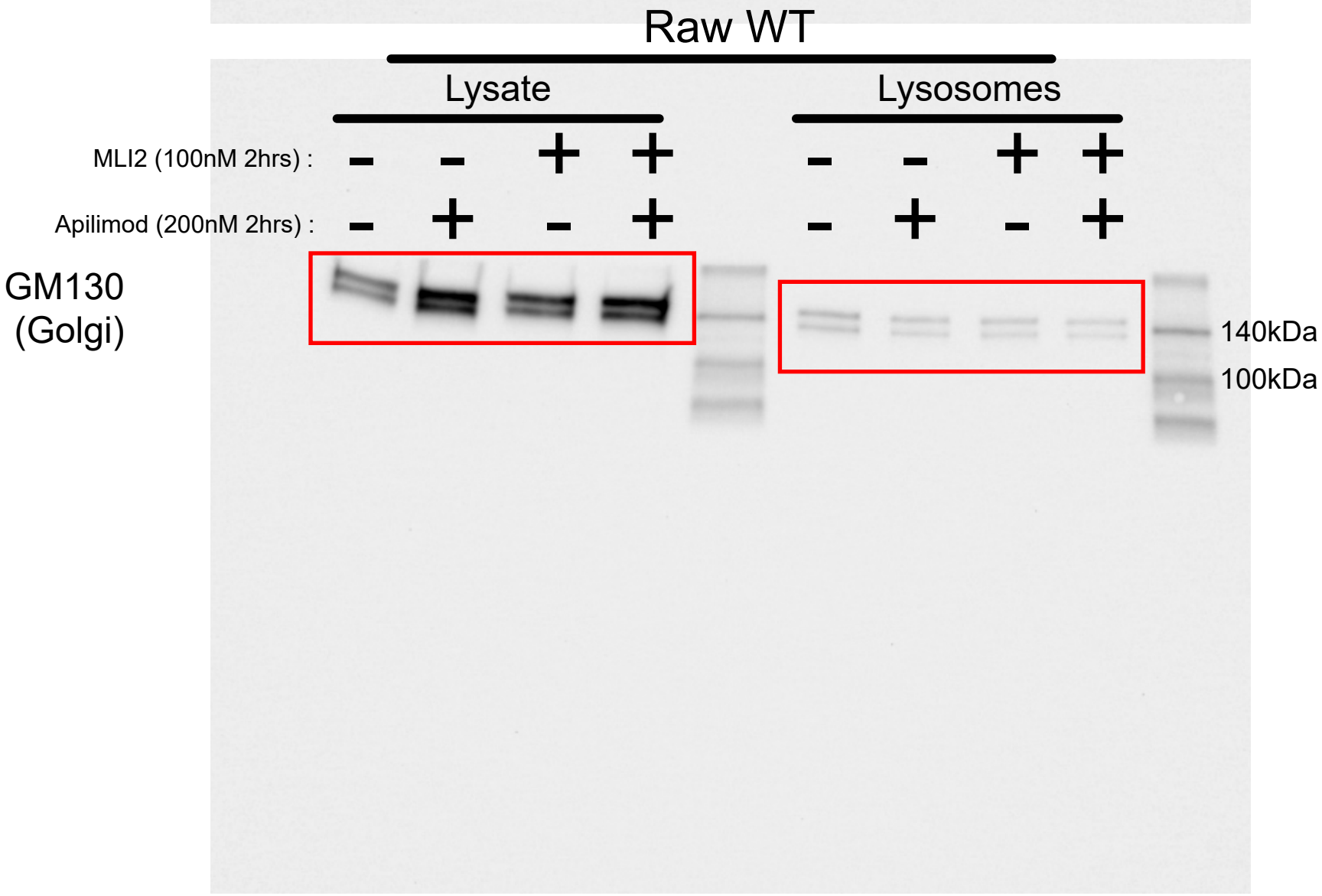

Raw WT
Lysate
Lysosomes
MLI2 (100nM 2hrs) :
Apilimod (200nM 2hrs) :
GM130
(Golgi)
140kDa
100kDa

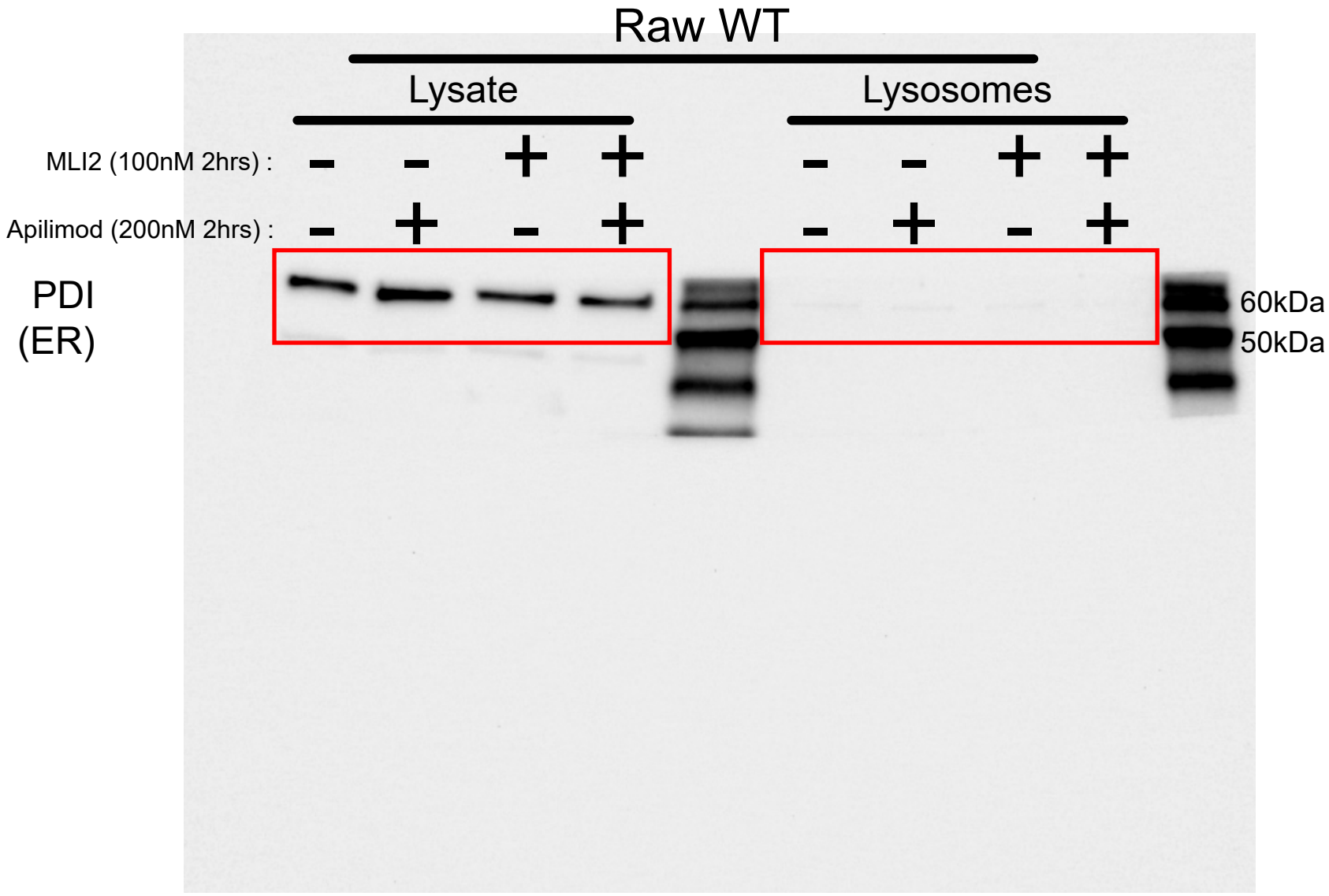

Raw WT
Lysate
Lysosomes
MLI2 (100nM 2hrs) :
Apilimod (200nM 2hrs) :
PDI
(ER)
60kDa
50kDa
